# m^6^A mRNA modifications are deposited in nascent pre-mRNA and are not required for splicing but do specify cytoplasmic turnover

**DOI:** 10.1101/gad.301036.117

**Published:** 2017-05-15

**Authors:** Shengdong Ke, Amy Pandya-Jones, Yuhki Saito, John J. Fak, Cathrine Broberg Vågbø, Shay Geula, Jacob H. Hanna, Douglas L. Black, James E. Darnell, Robert B. Darnell

**Affiliations:** 1 Laboratory of Molecular Neuro-Oncology, The Rockefeller University, New York, New York 10065, USA;; 2 Howard Hughes Medical Institute, The Rockefeller University, New York, New York 10065, USA;; 3 Department of Microbiology, Immunology, and Molecular Genetics, University of California at Los Angeles, Los Angeles, California 90095, USA;; 4 Proteomics and Metabolomics Core Facility, Department of Cancer Research and Molecular Medicine, Norwegian University of Science and Technology, 7489 Trondheim, Norway;; 5 The Department of Molecular Genetics, Weizmann Institute of Science, Rehovot 7610001, Israel;; 6 Laboratory of Molecular Cell Biology, The Rockefeller University, New York, New York 10065, USA

**Keywords:** m^6^A-CLIP, pre-mRNA, cell fractionation, mRNA turnover

## Abstract

Understanding the biologic role of *N*^6^-methyladenosine (m^6^A) RNA modifications in mRNA requires an understanding of when and where in the life of a pre-mRNA transcript the modifications are made. We found that HeLa cell chromatin-associated nascent pre-mRNA (CA-RNA) contains many unspliced introns and m^6^A in exons but very rarely in introns. The m^6^A methylation is essentially completed upon the release of mRNA into the nucleoplasm. Furthermore, the content and location of each m^6^A modification in steady-state cytoplasmic mRNA are largely indistinguishable from those in the newly synthesized CA-RNA or nucleoplasmic mRNA. This result suggests that quantitatively little methylation or demethylation occurs in cytoplasmic mRNA. In addition, only ∼10% of m^6^As in CA-RNA are within 50 nucleotides of 5′ or 3′ splice sites, and the vast majority of exons harboring m^6^A in wild-type mouse stem cells is spliced the same in cells lacking the major m^6^A methyltransferase Mettl3. Both HeLa and mouse embryonic stem cell mRNAs harboring m^6^As have shorter half-lives, and thousands of these mRNAs have increased half-lives (twofold or more) in Mettl3 knockout cells compared with wild type. In summary, m^6^A is added to exons before or soon after exon definition in nascent pre-mRNA, and while m^6^A is not required for most splicing, its addition in the nascent transcript is a determinant of cytoplasmic mRNA stability.

## Abstract (precis)

In this study, Ke et al. investigated the role of m^6^A RNA modifications in mRNA, including when and where in the life of a pre-mRNA transcript the modifications are made. They found that m^6^A is added to exons before or soon after exon definition in nascent pre-mRNA, and its addition in the nascent transcript is a determinant of cytoplasmic mRNA stability.

Studying nascent RNA synthesis in cultured cells using very brief pulse labeling with radioactive nucleosides allowed a number of advances in understanding pre-mRNA synthesis and processing in the era before rapid nucleic acid sequencing. Examples include polyA addition on pre-mRNA before completion of mRNA processing and cytoplasmic entry (Darnell et al. 1971; Edmonds et al. 1971) and locating the first known boundaries of eukaryotic polymerase II transcription units through studying labeled nascent adenovirus transcripts (Bachenheimer and Darnell 1975; Evans et al. 1977; Weber et al. 1977).

These early experiments were joined by a cell fractionation technique originated by Wuarin and Schibler (1994) that uses a 1 M urea solution to liberate a “chromatin” fraction from nuclei. This fraction provides a stringent purification of growing nascent pre-mRNA chains, isolated as a chromatin-associated RNA fraction (referred to as CA-RNA), along with RNA polymerase II plus all nuclear DNA and associated histones. Using specific labeled DNA probes, Wuarin and Schibler (1994) demonstrated removal in liver cell nuclei of some, but not all, introns from two specific nascent pre-mRNAs: a transcription factor pre-mRNA involved in circadian rhythm and the HMG coA reductase pre-mRNA. Recently, Pandya-Jones and Black (2009) adapted this procedure to study the extent and order of intron removal in cultured human carcinoma cell nuclei, again showing that many, but not all, introns are removed in CA-RNA.

Current sequencing techniques allow a complete analysis of the three cellular RNA fractions: CA-RNA, nucleoplasm, and cytoplasm. Bhatt et al. (2012) and Pandya-Jones et al. (2013) recently reported sequencing analyses of these three RNA fractions at various times after gene induction of toxin-responsive genes in macrophages. Major conclusions were that (1) many, but not all, introns are removed from the pre-mRNA of the induced genes in the CA-RNA during pre-mRNA processing and prior to transfer to the nucleoplasm, by which time essentially all introns had been removed, as observed for the cytoplasmic RNA. (2) Individual intron removal time varied greatly from a few to many minutes in different individual induced and constitutive mRNAs. The order of removal generally favored 5′-to-3′ removal, but this was definitely not universally true. (3) All analyzed chains had the 3′ polyA added before completing intron removal and departure from the chromatin fraction. (4) Within minutes of the appearance of these specific induced mRNAs in the nucleoplasm, they appeared in the cytoplasm. In an independent study, Khodor et al. (2011, 2012) also used this nascent chain technique coupled with sequencing in mouse cells and *Drosophila* tissue to demonstrate partial intron removal in CA-RNA.

Examination of RNA separated into these three fractions is vastly more informative concerning events in the synthesis and processing of pre-mRNA than the now almost universally performed extraction of total RNAs or examination of crude whole nuclear preparations.

## Methylation of N^6^ position in adenosine residues in mRNA

Methylation on the N^6^ position of adenosine in eukaryotic polyA^+^ mRNA from polysomes was discovered in cultured mammalian cells in 1974 (Perry and Kelley 1974) followed by many additional studies (Desrosiers et al. 1975; Furuichi et al. 1975; Wei et al. 1975), including demonstration of *N*^6^-methyladenosine (m^6^A) in high molecular nuclear DNA-like RNA (hnRNA) (Salditt-Georgieff et al. 1976), which contains pre-mRNA.

Since the development of a highly specific antibody to m^6^A (Bringmann and Luhrmann 1987), now commercially available, interest has returned recently to the study of this mRNA modification with considerable progress. Use of m^6^A antibody to precipitate oligonucleotides derived from total mammalian RNAs followed by sequencing (Dominissini et al. 2012; Meyer et al. 2012) revealed a general distribution of m^6^A in sequences that map to mRNAs and was reported as enriched around stop codons. Site-specific localization of m^6^A by m^6^A cross-linking immunoprecipitation (m^6^A-CLIP [Ke et al. 2015], modifying the CLIP technique [Ule et al. 2003]) demonstrated a precise enrichment of m^6^A in the start of 3′ exons but not around stop codons. Rather, m^6^A modification in last exons occurs more in terminal exons harboring 3′ untranslated regions (UTRs) than in coding sequences (CDSs) in both cultured human cells and mouse brains and livers, allowing possible 3′ UTR regulation, including alternative polyadenylation (Ke et al. 2015). Some recent experiments have begun to shed light on specific individual m^6^A additions to mRNAs with 5′ UTR that promote non-cap-dependent translation in the 5′ UTR (Meyer et al. 2015; Mitchell and Parker 2015; Zhou et al. 2015). This work deals with “stress” (heat shock) mRNAs and is thought to depend on the affinity of EIF3 for m^6^A. A separate clear finding shows that m^6^A_m_ on the first A residue in mRNAs inside the m^7^G of the cap structure at the 5′ end of mRNAs protects specific RNA from turnover (Mauer et al. 2017). Other studies have attempted to link m^6^A to generalized mRNA turnover (Wang et al. 2014a), translation efficiency (Wang et al. 2015), splicing (Dominissini et al. 2012), and alternative polyadenylation (Ke et al. 2015).

In this study, we first determined whether m^6^A is added to CA-RNA in the nucleus during pre-mRNA synthesis and processing and found that this methylation occurs in exonic but not intronic sequences in CA-RNA. We then compared the individual m^6^A present in HeLa cell pre-mRNA in CA-RNAs with the m^6^A content and location in the nucleoplasmic and cytoplasmic mRNAs and found it to be the same. One role of m^6^A in mRNA, first suggested in 1978 (Sommer et al. 1978) and recently suggested by Wang et al. (2014a), is to contribute to faster mRNA turnover. We used m^6^A-CLIP sequencing to quantitate the contribution to mRNA turnover in HeLa cells, normal mouse embryonic stem cells (ESCs), and Mettl3 knockout mouse ESCs. These results demonstrate that while m^6^A deposition occurs on nascent RNA in exons, it is not required for splicing but decreases cytoplasmic mRNA for those transcripts.

## Results

### Analysis of HeLa cell RNA from chromatin, nucleoplasm, and cytoplasm

**Figure 1.**
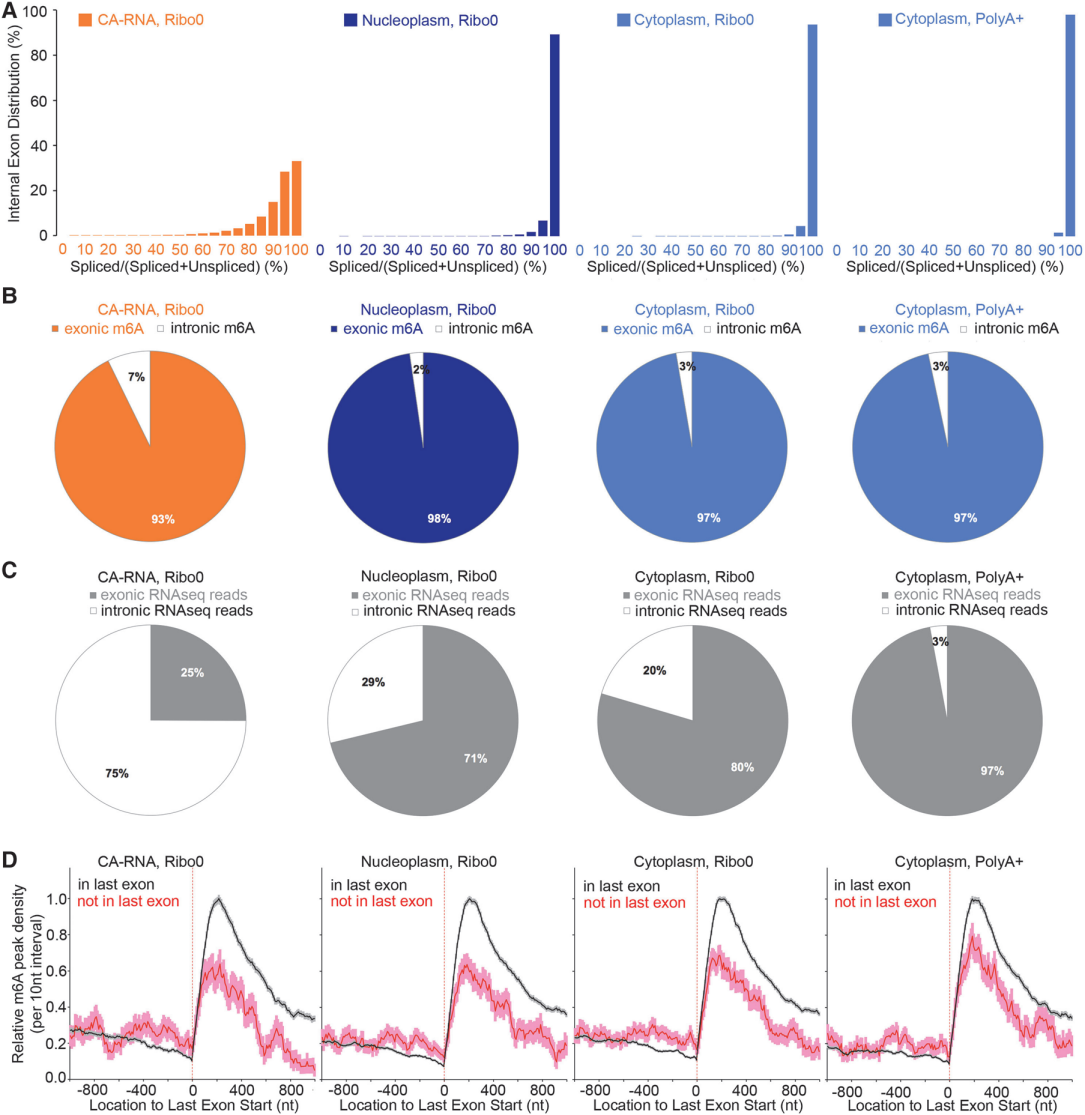
m^6^A modification occurs on chromatin (CA-RNA) in exons in nascent pre-mRNA. (*A*) Many exons are not yet spliced in CA-RNAs. Internal exons were obtained according to the gene structure annotation of GENCODE (version 19 of human hg19). PolyA^+^ RNAs were oligo dT-selected RNAs from total RNAs, and Ribo0 RNAs were total RNAs depleted of ribosome RNA by Ribo-Zero kit from Epicentre. (*B*) The great majority of m^6^As in pre-mRNA is in exons. Details of m^6^A peak calling are described in the Supplemental Material. In brief, for each m^6^A peak region, we enumerated reads of m^6^A immunoprecipitation and the input to evaluate the statistical significance (Fisher's exact test). Benjamini-Hochberg was implemented to adjust the *P*-value to the false discovery rate (FDR) for multiple testing. FDR <5%. (*C*) Chromatin pre-mRNAs that are partially spliced have more intronic RNA sequencing (RNA-seq) reads than exonic RNA-seq reads. (*D*) m^6^A peaks follow virtually the same distribution in RNAs from the three cell fractions. (Black line) mRNAs with a stop codon in last exon; (red line) mRNAs with a stop codon not in last exon; (gray and light-pink shaded regions) standard error of the mean (SEM). Not only is the distribution the same for m^6^A, but the m^6^A peak strength for each m^6^A also is mostly the same for each of the three cell fractions (see details in Fig. 2).

To compare m^6^A deposition in different cellular compartments, three RNA fractions—CA-RNA, nucleoplasm, and cytoplasm—were prepared from HeLa cells for sequence analysis, m^6^A location, and quantification (Fig. 1; Supplemental Fig. 1).

**Figure 2.**
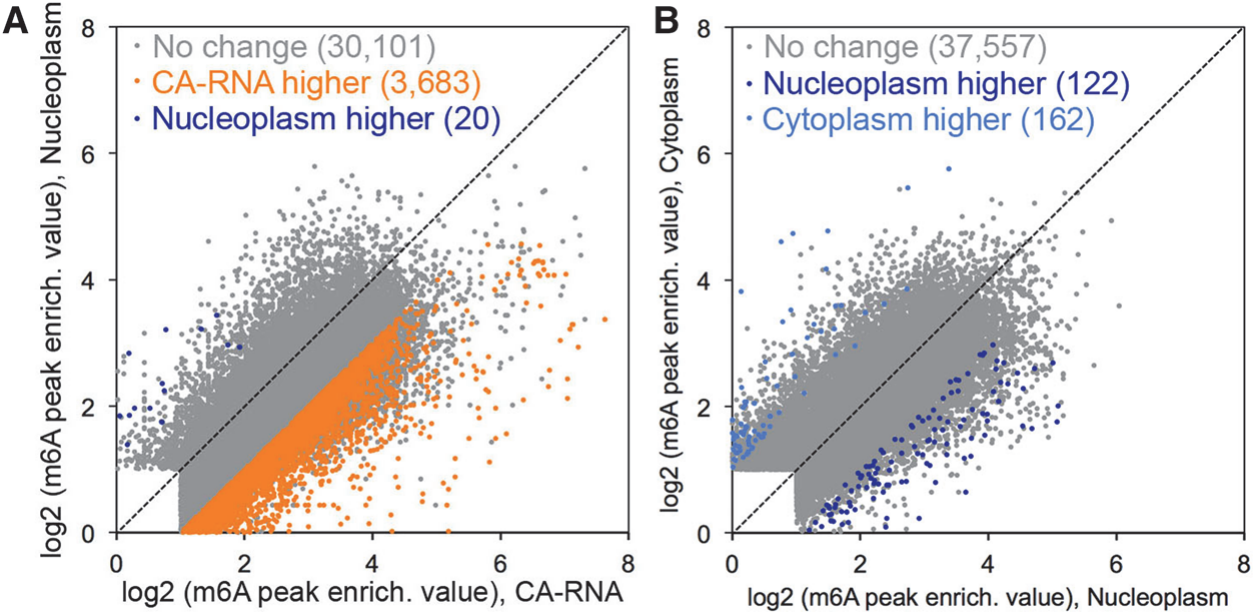
Each individual m^6^A modification is modified mostly with the same level for each of the three cell fractions. (*A*) Comparison of individual m^6^A peak signal strength in CA-RNA and nucleoplasmic RNA for the same m^6^A peaks (each dot is an individual m^6^A peak: no changes in gray, CA-RNA higher in orange, and nucleoplasm higher in dark blue). FDR <5%, Fisher's exact test. To determine m^6^A peaks that are higher in CA-RNA, for each m^6^A peak region, we enumerated reads of m^6^A immunoprecipitation and the input for CA-RNA and Nucleoplasm RNA, evaluating statistical significance with Fisher's exact test, using stringent FDR cutoffs to correct for multiple hypothesis testing. The determination that an m^6^A peak region was higher in CA-RNA required (1) the reads of mRNAs in m^6^A peak regions to be adequate for m^6^A peak region detection in both CA-RNA and nucleoplasmic mRNA (reads per kilobase per million mapped reads [RPKM] ≥1) and (2) FDR ≤0.05 and a twofold or higher of peak region enrichment in CA-RNA compared with nucleoplasmic mRNA. At a lower cutoff (e.g., ≥1.5-fold), the same conclusion held: that most m^6^A peaks are modified with the same level between CA-RNA and nucleoplasmic mRNA. (*B*) Comparison of individual m^6^A peak signal strength in nucleoplasmic RNA to cytoplasmic RNA for the same m^6^A peak. The same statistic criteria were used as in *A*. (Gray) No changes; (dark blue) nucleoplasm higher; (light blue) cytoplasm higher. FDR < 5%, Fisher's exact test. At a lower cutoff (e.g., ≥1.5-fold), again, the same conclusion held that most m^6^A peaks were modified at the same level in nucleoplasmic mRNA and cytoplasmic mRNA.

Sequence analysis of the three HeLa cell fractions (Fig. 1A) showed that ∼65% of the exons of nascent transcripts contained various numbers of introns in the CA-RNA fraction along with 35% of the exons of CA-RNA transcripts that were completely spliced. This agrees with earlier studies in other cells (Pandya-Jones and Black 2009; Bhatt et al. 2012; Khodor et al. 2012; Pandya-Jones et al. 2013). We proceeded to precisely map m^6^A using the m^6^A-CLIP method (Ke et al. 2015). Isolated RNA was fragmented to ∼80 nucleotides (nt) followed by precipitation of m^6^A-containing oligonucleotides by m^6^A-specific antibody, cross-linking by UV irradiation of the bound RNA to antibody, collection of the bound complex, and release of methylated RNA by protein digestion followed by cDNA library construction and RNA sequencing (RNA-seq) (Ule et al. 2003; Licatalosi et al. 2008). Reverse transcription of RNA containing an m^6^A cross-linked peptide/amino acid or even m^6^A modification itself (Ke et al. 2015) occasionally results in detectable diagnostic sequence errors at the m^6^A sites, including cross-linking-induced mutation sites (CIMS; such as single-base substitutions, deletions, and insertions) (Zhang and Darnell 2011), cross-linking-induced truncation sites (CITSs) (Konig et al. 2010), and m^6^A-induced truncation sites (MITSs) (Ke et al. 2015). These changes allow precise identification of the sites of m^6^A modification. The m^6^A antibody-enriched RNA regions are quantified as m^6^A peak regions or m^6^A peaks, within which the consensus m^6^A site at the mutational or truncation site precisely identifies m^6^A location (Ke et al. 2015). We included in the Supplemental Material a protocol for m^6^A-CLIP with a detailed day-to-day arrangement of experiments.

### m^6^A addition to CA-RNA greatly favors exons over introns

Given that HeLa cell CA-RNA retains many introns (Fig. 1A), a major initial result was that ∼93% of the m^6^As in the partially spliced CA-RNAs were in exons (Fig. 1B; Supplemental Fig. 2) despite the fact that there was about three times as much intronic as exonic sequence within the partially spliced CA-RNA (Fig. 1C). In both the nucleoplasm and cytoplasm, ∼98% of the m^6^As were identified in exons. Therefore, an A residue in an intron has a small chance of being methylated compared with an A residue in an exon.

### m^6^A distribution in the 3′-proximal RNA fractions of nascent pre-mRNA molecules

In our previous work (Ke et al. 2015), we found a large fraction (∼70%) of m^6^As in total steady-state mRNA in cultured cells and tissues to be in last exons, about two-thirds of which are in the 3′ UTR sequence before the 3′ polyA terminus of an mRNA.

Our finding that m^6^A is added mostly to exons, not introns, in CA-RNA prompted us to compare the previously noted peak distribution of m^6^A within the steady-state 3′-terminal exons in total cell RNA with that in each of the cell fractions. Both the specific sequence location (Fig. 1D; Supplemental Fig. 3) and the modification level (Fig. 2A,B, each dot is an individual m^6^A peak) of each m^6^A peak in CA-RNA were quantitatively very similar to that in the nucleoplasmic and cytoplasmic fractions. This result suggests that the methyltransferase complex (Liu et al. 2014; Ping et al. 2014) plus associated proteins (Bokar et al. 1994, 1997) must deposit m^6^A soon after chain synthesis regardless of whether splicing has occurred and that there is no frequent methylation/demethylation occurring past the pre-mRNA stage (∼90% of m^6^A peaks are unchanged when comparing CA-RNA and nucleoplasmic RNA [Fig. 2A], and >99% of 37,557 m^6^A peaks are unchanged when comparing nucleoplasmic and cytoplasmic mRNA peaks [Fig. 2B]).

We included an analysis of the ∼5% of primary transcripts that, when processed, do not have polyA added to the last coding exon but instead have it added to a 3′-terminal noncoding exon (Fig. 1D; Supplemental Fig. 3, red lines). The nascent chromosomal RNA of these particular transcripts also received a high level of methylation in the terminal exon, as did the great majority of conventional mRNAs (Fig. 1D; Supplemental Fig. 3, black line). This reconfirms that the actual enrichment of m^6^A in last exon (Ke et al. 2015) is established while pre-mRNA is still attached to chromatin.

In the experiments of Bhatt et al. (2012), it was established that cleavage and polyA addition were completed in most pre-mRNAs that are still associated with chromatin before splicing was completed. The present results show that m^6^A also is added to pre-mRNA before mRNA leaves the chromatin in what appears to be virtually all of the same modifications subsequently found in nucleoplasmic and cytoplasmic RNA.

### Frequency of m^6^A addition as a function of distance to the polyA site

To gain insight into m^6^A addition as a function of position of the polymerase along the gene, further analysis of the distribution in the CA-RNA was carried out (Supplemental Fig. 4). Internal exons (excluding first and last exons) on nascent chains were divided into four groups according to the distance of each exon to a polyA site: quartile 4 (Q4; the longest) and Q1 (the shortest). Splicing completion and the frequency of m^6^A in internal exons were assessed in each quartile (Supplemental Fig. 4). For example, Q1 contained nascent chains that were up to ∼6.4 kb, and Q4 contained the largest nascent chains, up to ∼1 Mb (Supplemental Fig. 4A). To calibrate the splicing completeness of internal exons on CA-RNAs, we calculated a splicing completion index for each internal exon in CA-RNA and nucleoplasmic mRNA, where the splicing completion index was defined as the percentage of spliced RNA molecules over the total RNA molecules, including both spliced and unspliced. The index difference for each exon between CA-RNA and nucleoplasmic mRNA measures the splicing completeness: The higher the difference, the less complete the splicing is. As described previously (Bhatt et al. 2012; Pandya-Jones et al. 2013), completion of splicing showed that the most 5′ –proximal internal exons were spliced more completely than the exons close to the polyA site (Supplemental Fig. 4A). Strikingly, exons in all four groups of nascent RNA had a similar frequency of exonic m^6^A residues (∼4% of exons in each group had at least one m^6^A) (Supplemental Fig. 4B). These results suggest that m^6^A addition can occur on exons in nascent chains regardless of whether splicing of the exons has occurred.

### Direct proof that m^6^A is added to exons in nascent pre-mRNA and that methylation can occur before splicing

**Figure 3.**
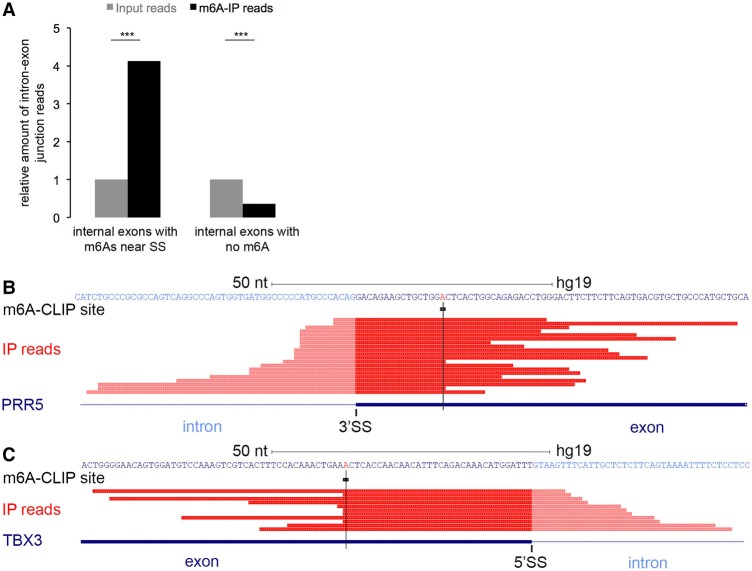
m^6^A can be added to exons before splicing. (*A*) Compared with input reads, m^6^A immunoprecipitation reads were enriched for pre-mRNA reads containing both intron and m^6^A-containing exon sequences (*left*) but depleted for exon–intron junction sequence fragments lacking m^6^A (*right*). (***) *P* < 10^−100^, Fisher's exact test. (*B*) An example of an internal exon in the PRR5 gene. “m^6^A-CLIP site” shows a precise m^6^A site (black box) identified by m^6^A-CLIP. “IP reads” lists the cDNA reads of RNA fragments that were precipitated by m^6^A-specific antibody and contain both the m^6^A site and the unspliced intronic region. This m^6^A site is near a 3′ splice site. (*C*) An internal exon in the TBX3 gene; this m^6^A site is near a 5′ splice site. More examples are in Supplemental Figure 5.

In the course of sample preparations to study m^6^A locations, RNA was reduced to fragments nominally 50–80 nt long, cross-linked to m^6^A antibody, reacted with m^6^A-specific antiserum, precipitated, and sequenced. If some of the m^6^A-containing fragments contained exon–intron junction sequences within the CA-RNA fraction, they should be greatly enriched by the m^6^A antibody precipitation. When we assessed this, we found that the m^6^A containing exon–intron junction sequence fragments were very uncommon but were significantly enriched after m^6^A antibody precipitation (Fig. 3A, left) compared with exon–intron junction sequence fragments lacking m^6^A (Fig. 3A, right). Two examples of junction sequences with an m^6^A still retaining exon–intron junctions are shown in Figure 3, B and C. The m^6^A is near a 5′ splice site in one and a 3′ splice site in the other. (It is important to recognize that this result does not comment on the density of m^6^A at splice sites; rather, it shows only that m^6^A addition has occurred before splicing is complete. The density of m^6^A around splice sites is discussed later.) Four more such examples are shown in Supplemental Figure 5. More than 200 cases were examined in which sequenced internal exons have m^6^A-containing exon–intron junction fragments (Supplemental Table 1); the m^6^A site in every examined exon–intron junction fragment from CA-RNA was within the exon, proving that methylation can (and apparently frequently does) occur before splicing is completed and reinforcing the conclusion that m^6^A addition is to exons but not introns. These observations strongly suggest that m^6^A is added to exons coincident with or soon after exon definition occurs in nascent pre-mRNA.

### The location of m^6^A residues relative to splice sites

With respect to a relation between m^6^A addition and splicing, two extreme possibilities exist: (1) m^6^A addition to an exon is independent of and not involved in intron removal or (2) intron removal depends on or is blocked by m^6^A sites in exons bordering introns.

As has been known for more than two decades, the favored methylation sequence for m^6^A modification in mRNA is RRACU or at least RAC (Harper et al. 1990). The short consensus at 5′ and 3′ splice sites (5′AG/GT --------NAG/GNN3′; no strict conservation) in higher eukaryotic RNA splicing seems to rule out any direct marking of splice sites by m^6^A in either directly locating splice sites or, alternatively, directly blocking splice sites.

**Figure 4.**
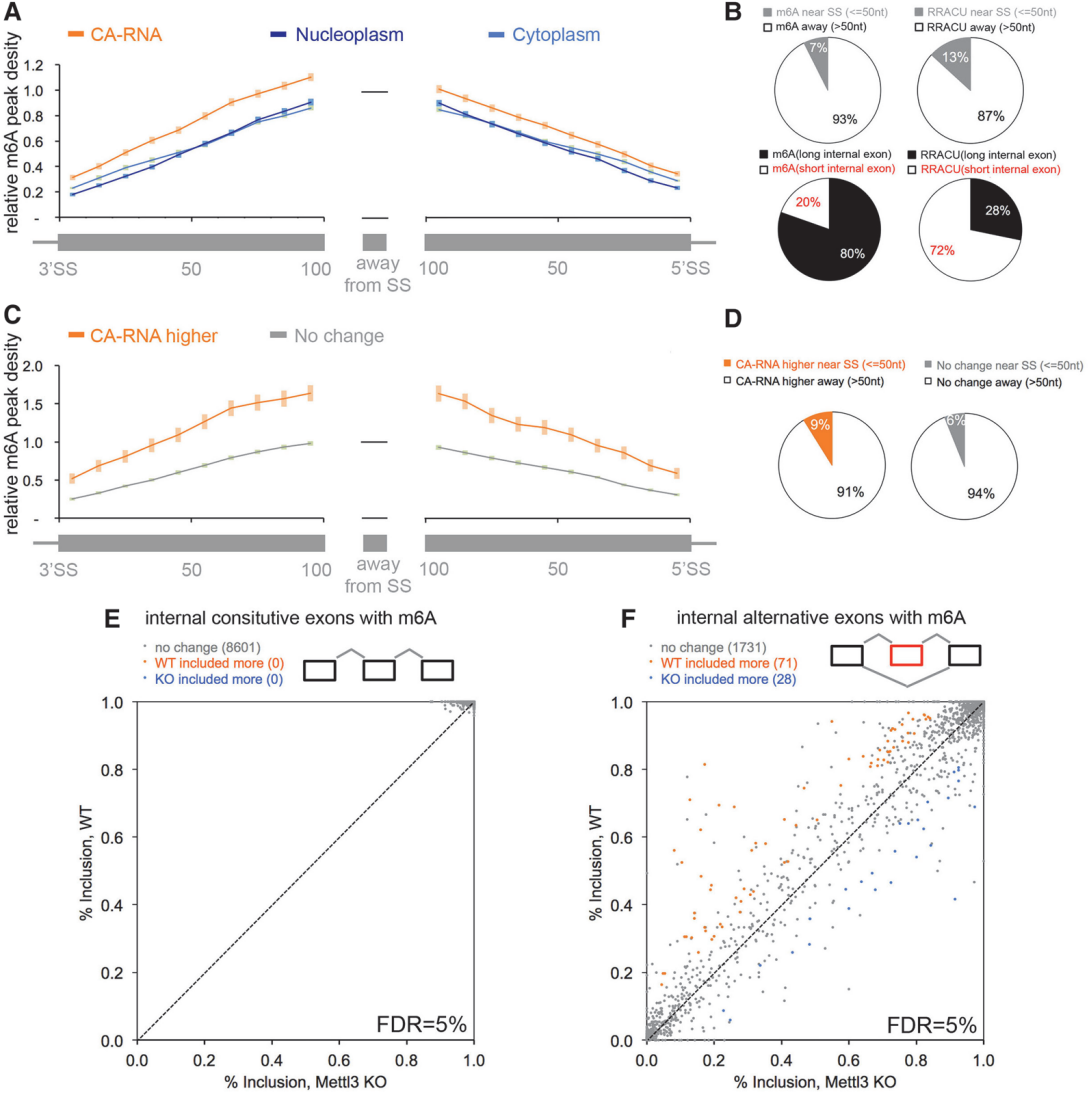
The majority of m^6^As is not located close to splice sites. (*A*) The density of m^6^A at increasing distances from 3′ or 5′ splice sites in CA-RNA (orange lines), the nucleoplasm (dark blue), and the cytoplasm (light blue). “Relative m^6^A peak density” for a fixed position from the splice site was calculated as the scaled m^6^A peak density at that position scaled proportional to the average m^6^A peak density in exonic regions >100 nt away from splice sites (black line). To clearly show the distribution of m^6^A peaks from the splice sites, we focused on internal exons with exon length at least 200 nt so that the 100-nt exon regions from the 5′ splice site (“SS”) and the 3′ splice site do not overlap. The internal exons 200 -nt long contain ∼80% of all internal exon m^6^As. The center exon is required to have m^6^A. Exon number = 3069. Error bar is the SEM. (*B*) Seven percent of exonic m^6^As are within 50 nt of splice sites for internal exons in *A*. (*Top left* panel) Few m^6^As locate close to splice sites. (*Top right* panel) More RRACUs locate close to splice sites. Total RRACU number is 5673 in the same exons. *P* < 1 × 10^−24^, Fisher's exact test. (*Bottom left* panel) Long internal exons (≥200 nt, the internal exons in *A*) have 80% of total internal exon m^6^As, while short ones (<200 nt) have only the remaining 20%. (*Bottom right* panel) Long internal exons have only 28% of the total internal exon RRACU motifs (total RRACU number is 46,492 for total internal exons; *P* < 1 × 10^−100^, Fisher's exact test), in contrast to short ones that have the majority (72%). Even if we consider all m^6^A-containing internal exons (both long and short), still, only 20% of total internal exonic m^6^As are within 50 nt of splice sites. (*C*) The CA-RNA m^6^A peaks that are higher showed higher density in exonic regions near 5′ or 3′ splice sites. “CA-RNA higher” refers to the m^6^A peaks in Figure 2A with higher m^6^A signal strength in CA-RNA (orange). “No change” refers to the frequency of m^6^A peaks with no difference between CA-RNAs and nucleoplasm RNAs in Figure 2A (gray). Error bars are SEM. (*D*) The majority (>90%) of transcripts in which m^6^As are higher in CA-RNA than nucleoplasmic RNA is at least 50 nt away from splice sites. A minority of CA-RNA transcripts does have a percentage greater density of m^6^A near splice sites (≤50 nt; 9% vs. 6%), but the majority of these modifications does not persist in nuceloplasmic RNA (Fig. 2A). (*E*) Internal constitutive exons containing m^6^A show no change in splicing between wild-type and Mettl3 knockout mouse ESCs. Internal constitutive exons with m^6^A are defined as the triexon structure, with constitutive exon being the center exon; at least one of the three exons should have m^6^A. All 8601 m^6^A-containing internal constitutive exons showed the same degree of exon inclusion in Mettl3 knockout and wild-type cells (significant changes are defined as ΔPSI [percent spiced in] ≥0.1; FDR <5%) despite a decrease in m^6^A level in mRNAs to 10% of wild type (Fig. 6A; Supplemental Fig. 11). (*F*) A minority of internal alternative cassette exons with m^6^A (defined as the triexon structure, with alternative cassette exon being the center exon; at least one of the three exons should have m^6^A) shows splicing changes in Mettl3 knockout versus wild-type cells. Approximately 5% of all 1830 m^6^A-containing internal alternative cassette exons changed splicing in Mettl3 knockout cells (significant changes are defined as ΔPSI ≥0.1; FDR <5%). We also examined the splicing for all constitutive exons and alternative exons regardless of whether they contain m^6^A or not (i.e., even considering the indirect effects of m^6^A on splicing). Again, all 67,706 constitutive exons spliced identically, and only a very minor proportion (∼3%) of all 11,715 alternative cassette exons changed splicing. Other alternative splicing types showed even fewer changes, including alternative 5′ splice site, alternative 3′ splice site, and intron retention. We also analyzed the raw RNA-seq data of previous publications that reported certain splicing changes upon comprising Mettl3 expression levels (Supplemental Fig. 9; Dominissini et al. 2012; Zhao et al. 2014; Geula et al. 2015; Liu et al. 2015) and found the same result: that exons splice mostly the same when their exonic m^6^As were lost.

Whether m^6^A might be located in an exonic splicing enhancer or exonic splicing silencer sequence cannot be determined directly from total sequence compilations (e.g., Supplemental Table 2), but the position of m^6^A residues with respect to intron–exon boundaries can be assayed. We therefore determined the proximity of m^6^As to splice sites. This positional assessment was carried out in all three RNA fractions: CA-RNA, nucleoplasmic RNA, and cytoplasmic RNA. Starting at either a 5′ or 3′ splice site and moving into the exon sequence, the m^6^A occurrence rises so that by ∼100 nt, the density equals the average density in all internal exonic regions (Fig. 4A). The occurrence in the first 50 nt away from the splice junction (3′ or 5′) is only half the average occurrence of that in internal exonic regions (Fig. 4A,B). Furthermore, only 7% of m^6^As are within 50 nt of splice sites in these internal exons (Fig. 4B, top left), although this proximal splice site region contains 13% of total RRACUs in the internal exons (i.e., ∼50% less of the RRm^6^ACU motif compared with the RRACU motif for the 50-nt region near the splice site region; *P* < 10^−24^, Fisher's exact test) (Fig. 4B, top right).

We also noted that certain internal exons show relative enrichment of m^6^As: Long exons (≥200 nt) contain 80% of all internal exon m^6^As while containing only 28% of total (modified + unmodified) internal exon RRACU motifs (i.e., >10-fold enrichment of RRm^6^ACU compared with the RRACU motif in exons >200 nt; *P* < 10^−100^, Fisher's exact test) (Fig. 4B, bottom two panels).

In the total CA-RNA, there was an average of ∼10%–15% more m^6^A in the 100 nt near both the 3′ or 5′ splice junctions than the same regions in either the nucleoplasmic or cytoplasmic RNA (Fig. 4A). Furthermore, we found that while ∼30,000 m^6^A peaks are present at the same frequency in chromatin and nucleoplasmic fractions, ∼4000 individual m^6^A peaks had a higher frequency in CA-RNA compared with nucleoplasmic RNA (Fig. 2A). The majority (91%) of the m^6^As that are higher in CA-RNA is at least 50 nt away from splice sites (Fig. 4D). These modifications are preferentially located in internal constitutive exons (Supplemental Fig. 6). Those m^6^A peaks that are higher in CA-RNA show a 50% higher frequency in both 5′ and 3′ junctions in exons (Fig. 4C). However, this m^6^A enrichment in exons near splice junctions in the CA-RNA of individual pre-mRNAs does not persist in nucleoplasmic or cytoplasmic RNA of these specific transcripts (Fig. 2A). It seems possible that these m^6^A residues are removed by demethylases prior to nuclear exit.

We also examined specifically the residues at the 5′ and 3′ borders of pre-mRNAs that harbor alternatively spliced exons. As was true for 5′ and 3′ splice sites, in general, there was no accumulation of m^6^A residues located at splice sites of these alternatively spliced exons (Supplemental Fig. 7A). We also examined sequences bordering alternatively spliced exons in mouse ESCs (discussed fully later; see Supplemental Fig. 7B) and likewise found no heightened collection of m^6^A residues at exon–intron junctions.

This is in sharp contrast to recent reports (Zhao et al. 2014; Xiao et al. 2016) claiming multiple distinct deposits of m^6^A at both 5′ and 3′ splice sites of thousands of mRNAs in HeLa and 3T3 cells. Hence, we reanalyzed raw sequencing data from different laboratories (Supplemental Fig. 8) and were unable to reproduce the findings of m^6^A heightened at splice sites (Zhao et al. 2014) but were able to reproduce results from two independent groups (the Regev group [Schwartz et al. 2014] and the Jaffrey group [Meyer et al. 2012]) that were consistent with ours. In summary, the data indicate that the bulk of m^6^A modifications is located internally in exons and that there is no enrichment of m^6^A at splice junctions, while there is no absolute prohibition of m^6^A sites within 50 nt of splice sites (see Fig. 4; Supplemental Figs. 7, 8).

### Comparison of mRNA splicing in wild-type and Mettl3 knockout ESCs

We next quantitatively examined several properties of mRNA from growing normal ESCs and the same cell type with a knockout of the Mettl3 gene (Geula et al. 2015). Mettl3 was the first cloned mRNA m^6^A methyltransferase (Bokar et al. 1997) and is the major methyltransferase in a larger methylation complex (Wang et al. 2016a,b). A recent study (Schwartz et al. 2014) established that the Mettl3/Mettl14/WTAP protein complex is necessary for the m^6^A methylation of mRNAs in A549 cells. Mettl3 knockout cells no longer differentiate upon stimulation but continue to grow in appropriate medium even though the mRNA m^6^A content is ∼10% of normal (see Fig. 6A, below). We first examined the effects on RNA profiles and splicing that might be caused by Mettl3 deletion. The profiles of the mRNAs in the two cell samples were largely overlapping, and, with a statistical cutoff commonly used in splicing research for reliable splicing detection (false discovery rate [FDR] <5% and ΔPSI [percent spliced in] ≥0.1), all m^6^A-containing constitutive exons (>8000 constitutive exons) were spliced quantitatively the same in wild-type and knockout cells (Fig. 4E).

We also examined ∼2000 alternately spliced cassette exons containing m^6^As in the presence or absence of Mettl3. In Mettl3 knockout cells, only 4% of these alternatively spliced cassette exons showed more frequent inclusion, and 1% showed less frequent inclusion of the alternative exon (where splicing changes were defined as FDR <5% and ΔPSI ≥0.1) (Fig. 4F). We also examined the splicing for all constitutive exons and alternative exons regardless of whether they contained m^6^A (i.e., even considering the indirect effects of m^6^A on splicing). Again, >67,000 constitutive exons were spliced identically, and only a very minor proportion (∼3%) of ∼12,000 alternative cassette exons changed splicing (see details in the legend for Fig. 4F). Thus, after the removal of >90% of m^6^A by the knockout of Mettl3, there is no detectable effect on constitutive exons and a minor effect on mRNA splicing of alternative exons containing m^6^A. Furthermore, we downloaded the raw RNA-seq data from previous publications (Dominissini et al. 2012; Zhao et al. 2014; Liu et al. 2015) that reported certain splicing changes after Mettl3 knockdown, performed the same splicing analysis commonly used in splicing research for reliable splicing detection, and observed essentially the same result as these published data with our data: Loss of m^6^A has no detectable effect on constitutive splicing and a minor effect on the splicing of alternative exons with m^6^A modification (Supplemental Fig. 9).

### m^6^A deposition correlates with rapid mRNA turnover

Mammalian cell mRNAs were shown decades ago to have half-lives (*T*_1/2_s) varying from ∼1 h or less (Puckett et al. 1975; Puckett and Darnell 1977) to ≥12 h (Murphy and Attardi 1973; Singer and Penman 1973; Perry et al. 1974). Furthermore, the most rapidly labeled mRNAs are enriched in short-lived mRNAs compared with mRNAs labeled for 24 h (Harpold et al. 1981). Labeled nascent RNA reflects “rate of synthesis”; stable RNAs in cytoplasm will certainly be labeled longer than unstable RNAs that are labeled briefly in steady state (Harpold et al. 1981). The briefly labeled mRNAs were also found to be enriched 2.5-fold in m^6^As compared with stable mRNAs (Sommer et al. 1978). Recent reports using sequence analysis have now also suggested a link between m^6^A content and mRNA *T*_1/2_ (Batista et al. 2014; Schwartz et al. 2014; Wang et al. 2014a,b; Geula et al. 2015). Extensive *T*_1/2_ measurements for individual sequenced mammalian mRNAs that are available after actinomycin D treatment of cultured cells suggest a wide spectrum of *T*_1/2_s, from as little as ≤1 h to ≥10 h (Yang et al. 2003; Sharova et al. 2009; Tani et al. 2012).

**Figure 5.**
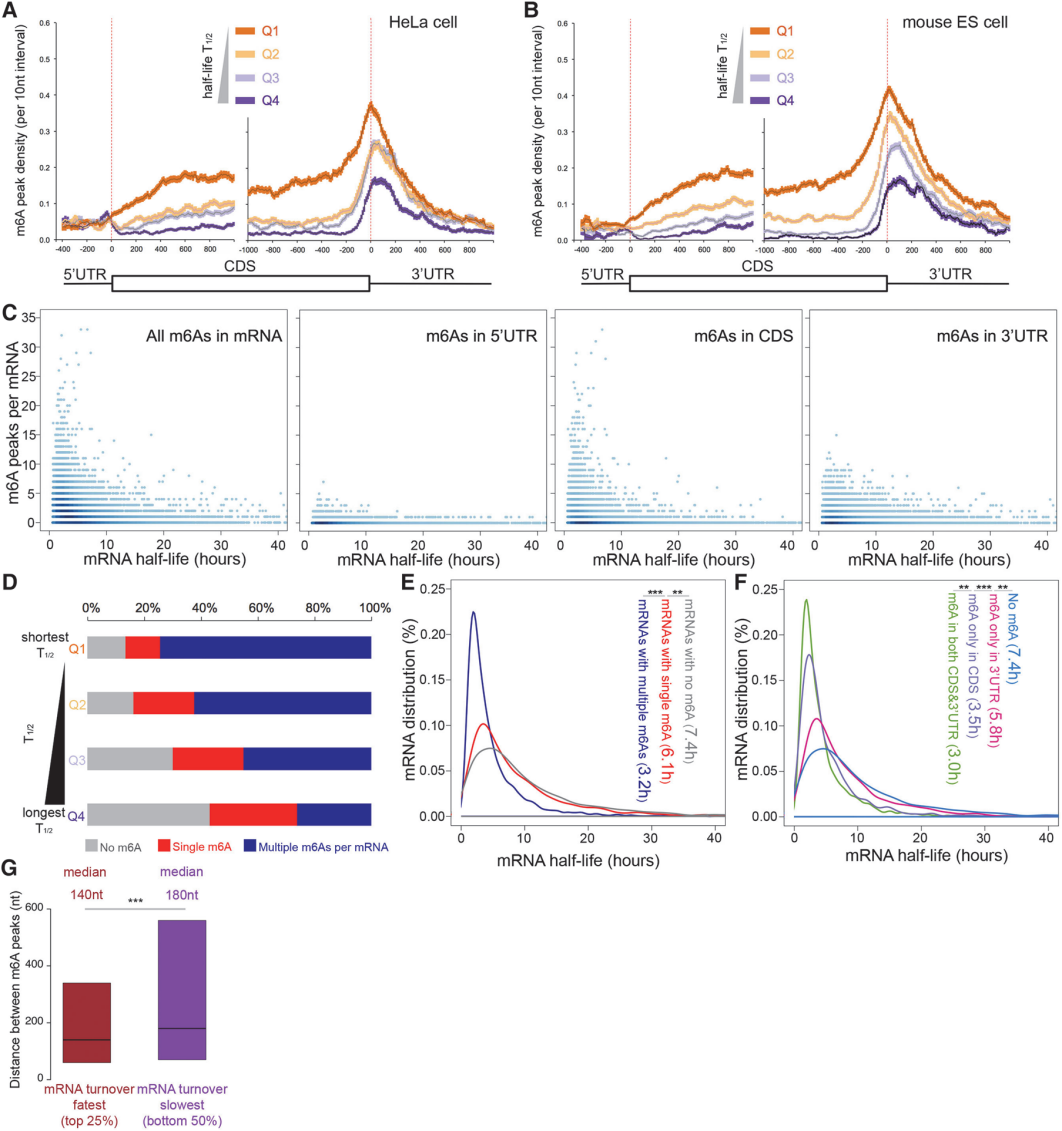
mRNAs with short *T*_1/2_s are enriched for multiple m^6^As. (*A*) mRNAs with shorter *T*_1/2_s have higher m^6^A density in HeLa cells. Tani et al. (2012) and our data on m^6^A location within the mRNAs were used to determine any correlation between *T*_1/2_ and m^6^A content. (*B*) mRNAs with shorter *T*_1/2_s have higher m^6^A density in mouse ESCs. (*C*) The scatter density plot of m^6^A peak numbers for mRNA *T*_1/2_s for individual mRNAs (dots). (*D*) Proportion of mRNAs with no, one, or multiple m^6^As in four quartiles of mRNAs with different *T*_1/2_s (a range of 0.6–40 h). (*E*) The amount of mRNA versus mRNA *T*_1/2_, grouped by m^6^A numbers per mRNA. The *T*_1/2_ distribution of mRNAs is plotted as a function of m^6^A content. (**) *P* < 10^−9^; (***) *P* < 10^−100^, Wilcox ranked test. (*F*) The effect of position of m^6^A on *T*_1/2_ within mRNA. (**) *P* < 10^−6^; (***) *P* < 10^−38^, Wilcox ranked test. There is little correlation between internal exon m^6^A (mostly CDS) and last exon m^6^A (mostly 3′ UTR). (*G*) m^6^A peaks on mRNAs with short *T*_1/2_s that tend to be more closely spaced on mRNAs with short *T*_1/2_s are labeled in brown; mRNAs with long *T*_1/2_s are labeled in purple. (***) *P* < 10^−20^, Wilcox ranked test. We observed the same result even when matching the m^6^A numbers in both groups of mRNAs.

We examined available *T*_1/2_ data for ∼8000 individual HeLa cell mRNAs (Tani et al. 2012). The mRNAs were divided into four quartiles (Q1–Q4; fastest to slowest turnover). m^6^A content of individual mRNAs in each quartile was then determined and plotted. Figure 5A shows m^6^A content in each quartile plotted on the *Y*-axis by position in the mRNA chains, anchored at start and stop codons. The density of m^6^A, normalized for length, among all exons was greatest in the coding region and the 3′ UTR of those mRNAs (anchoring at last exon start yields the same result) (Fig. 5A; Supplemental Fig. 10A). These most unstable human cell RNAs (Q1) have approximately two to four times more m^6^A than the most stable (Q4) HeLa cell mRNAs in exons of both the coding region and the 3′ UTR. Similar associations of more m^6^As in mRNAs with shorter *T*_1/2_s were obtained using the earlier *T*_1/2_ data of our group from human hepatoma cell (Supplemental Fig. 10B; Yang et al. 2003). We checked whether there is a correlation between the occurrence of m^6^A in the CDS versus the 3′ UTR of the same RNA and found none (*R*^2^ = 0.01 on a transcript basis); thus, m^6^A in both the CDS and the 3′ UTR specifies mRNA turnover independently.

### Comparison of mRNA *T*_1/2_s in wild-type and Mettl3 knockout ESCs

We next analyzed the location of m^6^A in transcripts showing changes in *T*_1/2_s in wild-type versus Mettl3 knockout ESCs. We first determined the *T*_1/2_s of individual polyA^+^ mRNAs in wild-type and Mettl3 knockout ESCs by sequencing polyA^+^ mRNA at various times after actinomycin D treatment. The *T*_1/2_s of individual polyA^+^ mRNAs were reproducible in biological triplicates, and the integrated data of individual replicates were used in the following analyses. Knowing the *T*_1/2_s of many mRNAs, we then determined the m^6^A content of all of these mRNAs. As in HeLa cells, the mRNAs with the shortest *T*_1/2_s had the highest m^6^A density (Fig. 5B; Supplemental Fig. 10C). Also, as in HeLa cells, the highest frequency of m^6^A is in the last exons, the CDSs, and the 3′ UTRs (Fig. 5B; Supplemental Fig. 10C).

With the *T*_1/2_ data and the m^6^A information of individual mRNAs, it was possible to directly relate the two (Fig. 5C). A plot of the number of m^6^As as a function of mRNA *T*_1/2_ in individual mRNAs emphasized strongly the high correlation between m^6^A content and shorter *T*_1/2_s (Fig. 5C, left panel). Many mRNAs with a *T*_1/2_ of 5 h or less had 10 m^6^As and some had even more. mRNAs with *T*_1/2_s of 10–20 h or >20 h had a progressively smaller number of m^6^A peaks.

The distribution of individual mRNAs with no, one, or multiple m^6^A peaks is shown in Figure 5, D and E. The quartile of mRNAs with the shortest *T*_1/2_ had only ∼10%–12% mRNAs with no m^6^A, whereas the quartile with the longest *T*_1/2_ had almost 40% of individual mRNAs with no m^6^A (Fig. 5D). Furthermore, the mRNAs with the shortest *T*_1/2_ had ∼75% of individual mRNAs with two or more m^6^As compared with 25% in mRNAs with the longest *T*_1/2_. The mRNA *T*_1/2_ distribution showed that mRNAs with multiple m^6^As have a median *T*_1/2_ less than half that of mRNAs with no m^6^A (Fig. 5E).

However, it is important to point out that many mRNAs lacking m^6^A still have a *T*_1/2_ of ≤5 h, and some of these mRNAs have a *T*_1/2_ of only 1 or 2 h (Fig. 5E). Clearly, there are triggers for turnover other than m^6^A. Compared with mRNAs with no m^6^As, mRNAs with m^6^As in the 3′ UTR have a faster median *T*_1/2_ (Fig. 5F), and the *T*_1/2_ is even faster for mRNAs with m^6^A in the CDS. The group of mRNAs with m^6^As in both the CDS and the 3′ UTR have the fastest median *T*_1/2_, almost double that of mRNAs with no m^6^A (Fig. 5F). Finally, the distance between individual m^6^A peaks in the 25% of mRNAs with the shortest *T*_1/2_ was less than the 50% of mRNAs with the longest *T*_1/2_ (Fig. 5G). This might mean it will eventually be found that clustered m^6^As function in initiating rapid mRNA turnover.

### Further effects on methylation in Mettl3 knockout cells

**Figure 6.**
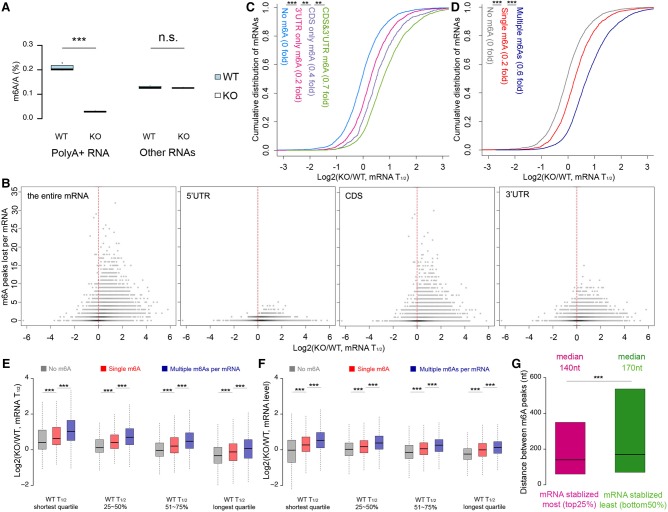
Data analysis of mouse ESC m^6^A residues in mRNA. (*A*) Global reduction of m^6^A after Mettl3 knockout. (***) *P* < 10^−5^; (n.s.) not significant, two-sample *t*-test. (*B*) Scatter density plot of the number of m^6^A peaks lost per mRNA versus mRNA *T*_1/2_ changes. “m^6^A peaks lost” refers to m^6^A peaks that were detected in wild-type mRNA but not in knockout mRNA (red dots in Supplemental Fig. 11). (*C*) Cumulative distribution plot of mRNAs versus mRNA *T*_1/2_ changes upon global m^6^A loss, grouped by the different regions of mRNA where m^6^A loss occurred. (**) *P* < 10^−10^; (***) *P* < 10^−100^, Kolmogorov-Smirnov test. (*D*) Cumulative distribution plot of mRNA versus mRNA *T*_1/2_ changes upon global m^6^A loss, grouped by the number of m^6^A peak loss per mRNA. (***) *P* < 10^−40^, Kolmogorov-Smirnov test. (*E*) The effect of m^6^A loss on mRNA *T*_1/2_ changes in different m^6^A content and different *T*_1/2_s in mouse ESCs. (Gray) No m^6^A per mRNA; (red) single m^6^A per mRNA; (blue) multiple m^6^As per mRNA. (***) *P* < 10^−5^; Wilcox ranked test. (*F*) Same mRNAs as in *E* but comparison is of steady-state mRNA levels from groups with different *T*_1/2_s. (***) *P* < 10^−5^, Wilcox ranked test. (*G*) m^6^As on mRNAs with *T*_1/2_s increased most upon global m^6^A loss tend to be clustered. The largest *T*_1/2_ effect upon global m^6^A loss is labeled in pink, and the average and least *T*_1/2_ effect are labeled in green. (***) *P* < 10^−15^; Wilcox ranked test. We observed the same result even when matching the m^6^A numbers in both groups of mRNAs.

The Mettl3 knockout in ESCs resulted in a great reduction in m^6^A in the polyA^+^ mRNA (90%) but had no detectable effect on methylation of other cellular RNAs (i.e., polyA^−^ RNA, most of which is rRNA) (Fig. 6A; Supplemental Fig. 11). The effects of Mettl3 knockout showed a fourfold to eightfold increase in *T*_1/2_ in many of the individual mRNAs regardless of whether the m^6^A peak loss was for the total mRNA or in the CDS or 3′ UTR (Fig. 6B). The quantitative effect was slightly greater when the m^6^A was in the CDS compared with the 3′ UTR, and simultaneous m^6^A losses in both the CDS and the 3′ UTR gave the maximum effect in increasing *T*_1/2_ (Fig. 6C). This longer *T*_1/2_ in the knockout cells was observed for mRNAs over the whole range of RNA *T*_1/2_s (Fig. 6B,E) and correlated with the m^6^A content; that is, multiple m^6^A loss increased *T*_1/2_ the greatest (Fig. 6B,E). The increased *T*_1/2_ related to m^6^A loss resulted in increased steady-state levels of the target mRNAs (Fig. 6F). We also noted that mRNAs with the greatest increase in *T*_1/2_s had more closely spaced m^6^A peaks than the m^6^A peaks in mRNAs with a smaller effect on the *T*_1/2_ of the knockout (Fig. 6G).

Although these results with both HeLa cells and mouse ESCs show a definite qualitative and quantitative contribution of m^6^A to mRNA turnover, every single m^6^A is not an obligatory signal for rapid turnover.

### Ontology of mRNAs with different *T*_1/2_s

**Figure 7.**
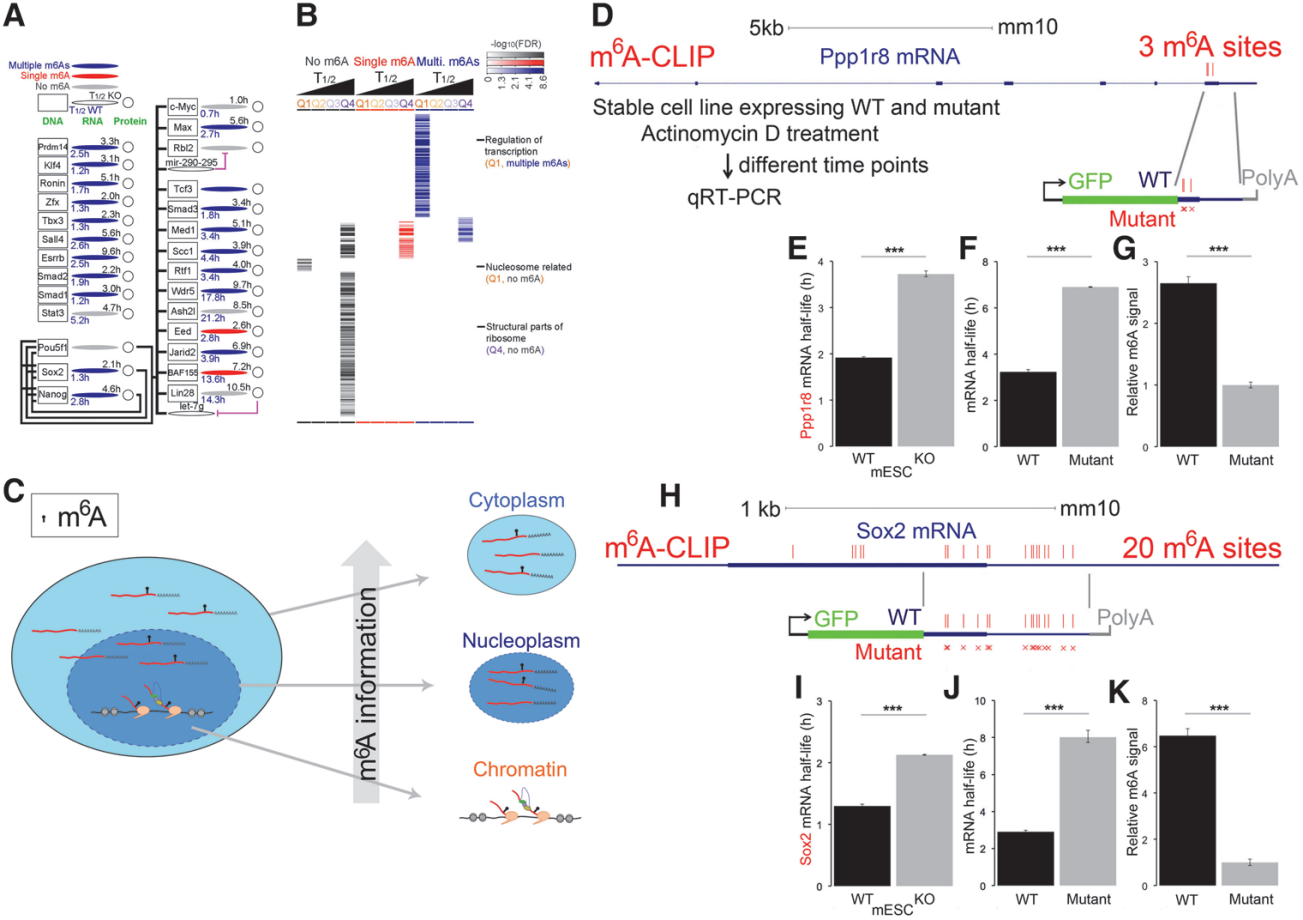
mRNAs with or without m^6^As are associated with important biological functions, and minigene mutational validation for m^6^A specifies mRNA turnover. (*A*) Most mRNAs of key genes controlling the ESC state (data modified from Young 2011; the detailed transcriptional regulation information and connection between terms are described fully in Young 2011) have multiple m^6^A peaks. mRNAs are symbolized as ellipse shapes. (Gray) No m^6^A; (red) single m^6^A; (dark blue) multiple m^6^A peaks; (*T*_1/2 WT_) the mRNA *T*_1/2_ in wild-type mouse ESCs; (*T*_1/2 KO_) the mRNA *T*_1/2_ in mouse ESCs with Mettl3 knockout (details in Fig. 6). The thick black lines connecting genes symbolize transcriptional regulation. (*B*) GO analysis of 12 groups of mRNAs (12 = 3 × 4: combinations of four *T*_1/2_ groups [Q1, Q2, Q3, and Q4] and three m^6^A groups [no m^6^A, single m^6^A, or multiple m^6^A peaks per mRNA]). The values of −log_10_(FDR) are illustrated as a heat map. None of mRNA groups in Q2 and Q3 *T*_1/2_ groups showed enrichment in any GOs. A specific GO example was provided for each of the three mRNAs groups: Q1 and multiple m^6^As, Q1 and no m^6^A, and Q4 and no m^6^A. (*C*) The m^6^A information flow from chromatin, nucleoplasm, and cytoplasm as an important implication. It is based on the fact that m^6^A modification is the same in the newly formed pre-mRNA as in the nucleoplasmic and steady-state cytoplasmic mRNAs (Fig. 2A,B). (*D*) Illustration of the minigene experiment, using Ppp1r8 as an example. The CDS of Ppp1r8 mRNA contains three precise m^6^A sites identified by m^6^A-CLIP (three vertical lines). This m^6^A-containing region was cloned to a minigene vector in-frame with GFP. Three synonymous point mutations (three red crosses) abolished the three precise m^6^A sites without altering the underlying amino acid sequence (detailed sequences for both the wild type and mutant are in the Supplemental Material). (*E*) Upon global loss of m^6^A in Mettl3 knockout mouse ESCs, which completely abolished the m^6^A peaks in Ppp1r8 mRNA, the mRNA *T*_1/2_ increased from 2 to 3.8 h. (***) *P* < 0.001, *t*-test. (*F*) Three synonymous mutations in the minigene increase the minigene mRNA *T*_1/2_ from ∼3 to ∼7 h. (***) *P* < 0.001, *t*-test. (*G*) Three synonymous mutations in the minigene decreased m^6^A signal in wild-type mRNA to approximately one-third in knockout mRNA. (***) *P* < 0.001, *t*-test. (*H*) Illustration of the minigene experiment in Sox2. The CDS of Sox2 mRNA contains 20 precise m^6^A sites identified by m^6^A-CLIP (20 vertical lines). A mRNA region containing 15 precise m^6^A sites was cloned to a minigene vector in-frame with GFP. Fifteen point mutations (illustrated by 15 red crosses: four synonymous mutations in the coding region and 11 point mutations in the 3′ UTR that mutate the “A” in RAC motif to “T”) abolished the 15 precise m^6^A sites without altering the underlying amino acid sequence (detailed sequences for both the wild type and mutant are in the Supplemental Material). (*I*) Upon global loss of m^6^A in Mettl3 knockout mouse ESCs, which completely abolished the m^6^A peaks in Sox2 mRNA, the mRNA *T*_1/2_ increased from 1.3 to 2.2 h. (***) *P* < 0.001, *t*-test. (*J*) Point mutations in the minigene increase the minigene mRNA *T*_1/2_ from ∼3 to ∼8 h. (***) *P* < 0.001, *t*-test. (*K*) Point mutations in the minigene decreased m^6^A signal in wild-type mRNA to approximately one-sixth in knockout mRNA. (***) *P* < 0.001, *t*-test.

Most mRNAs that encode key proteins in controlling the ESC state have multiple m^6^As and short *T*_1/2_s (Fig. 7A). As has been reported with fewer examples (Yang et al. 2003), gene ontology (GO) analysis revealed that *T*_1/2_ mRNAs with faster turnover and multiple m^6^As are associated with regulatory functions, such as transcriptional regulation (Fig. 7A,B), while more stable mRNAs with no m^6^As are associated with housekeeping functions, as illustrated by structural parts of the ribosome (Fig. 7B; Supplemental Tables 3, 4). Interestingly, mRNAs with a short *T*_1/2_ and no m^6^A are associated with nucleosome-related functions, such as nucleosome-related proteins (Fig. 7B; Supplemental Table 5).

## Discussion

The earlier conclusion that mRNA released into the nucleoplasmic fraction from CA-RNA is completely processed with respect to splicing and polyA addition (Pandya-Jones and Black 2009; Bhatt et al. 2012; Pandya-Jones et al. 2013) is corroborated and extended in the present work. Here we found that virtually all m^6^A methylation occurs at the pre-mRNA level during transcription and mRNA processing and is completed by the time an mRNA is released from chromatin into the nucleoplasmic RNA. Perhaps the most far-reaching new finding is that the vast majority of m^6^A addition occurs in exon sequences within CA-RNA—demonstrably, in many cases, before splicing has occurred—and not in introns even though the introns have many more total m^6^A target sequences (RAC or RRACU). Thanks to the precise identification of m^6^A sites afforded by m^6^A-CLIP (Ke et al. 2015), we conclude that the same m^6^A modifications (in both position [Fig. 1D] and robustness [Fig. 2A,B] for each m^6^A) in pre-mRNA are in the nucleoplasm and, finally, the cytoplasm. Thus, m^6^A modifications in pre-mRNA (CA-RNA) are not necessary for splicing but determine the subsequent role of m^6^A in cytoplasmic mRNA *T*_1/2_.

Several important conclusions follow from the results above. First, the proteins in the 800-kDa complex originally identified as necessary for in vitro methylation of mRNA (Rottman et al. 1994; Bokar et al. 1997) plus the m^6^A enzyme complex itself (Ping et al. 2014; Wang et al. 2016a,b) somehow distinguish between exon and intron as the nascent chain is being synthesized and before a great many introns are removed.

A similar conclusion was described in ^3^H uridine- and ^3^H methyl methionine-labeling experiments of late adenovirus mRNA formation. There, ∼14 different mRNAs are fashioned from a single 28-kb primary transcript (for review, see Nevins and Chen-Kiang 1981) that is always completed (Fraser et al. 1979) even though processing (polyA addition) begins before chain completion (Nevins and Darnell 1978). A kinetic analysis showed a greater conservation of ^3^H methyl label in pre-mRNA in the formation of adenoviral mRNA compared with the lesser conservation of ^3^H uridine (Nevins and Darnell 1978; Chen-Kiang et al. 1979). This early result implied that m^6^A is added only to that portion of the 28-kb primary transcript destined to be retained in one of the ∼14 different 2- to 4-kb mRNAs.

Thus, a crucial open question begs an answer: How does the m^6^A methylation complex know which newly formed pre-mRNA sequences will survive processing into mRNA? Put another way, how does m^6^A modification in pre-mRNAs occur with respect to “exon definition”? Recent experiments on the regulation of splicing have used a fractionation procedure that produced a high-molecular-weight (HMW) nuclear fraction that was released from chromatin and preserved some protein–protein interactions (Damianov et al. 2016). We tested whether antibody precipitation of the proteins in the HMW fraction would connect m^6^A methyltransferases and U1 snRNP and U2AF proteins, since exon definition is often hypothesized to depend on the initial U1 RNA binding at the 5′ splice site and U2AF at the 3′ splice site. In our hands, methyltransferase antibodies (Mettl3 or Mettl14) did not precipitate U1 snRNP or U2AF proteins, and U1 snRNP and U2AF protein antibodies did not precipitate Mettl3 or Mettl14 (Supplemental Fig. 12). A previous study reported a similar finding that U1 or U2 snRNP precipitation by antisense oligos could not pull down either Mettl3 or Mettl14 (Chu et al. 2015).

Another important implication arises from the fact that m^6^A modification is the same in the newly formed pre-mRNA as in the nucleoplasmic and steady-state cytoplasmic mRNAs (Fig. 7C, the m^6^A information flow from chromatin to nucleoplasm and cytoplasm). This result argues strongly against a primary role for “dynamic” regulation of methylation and demethylation that has been proposed (Fu et al. 2014).

With respect to splicing, the present work showed that ∼90% of the m^6^As in the internal coding exons are not within 50 nt of splice sites, and Mettl3 knockout cells have very few changes in either constitutive or alternative splicing. Furthermore, the Mettl3 knockout mouse embryonic cells make and splice mostly the same mRNAs as wild-type cells. Therefore, m^6^A is not obligatory for most splicing. It remains possible that a minority of m^6^As is involved in splicing choices.

### Conditions for chromosomal mRNA release

Is the completion of splicing the limiting factor for release of a completed mRNA molecule from the chromatin? Since many introns are removed from nascent chains before as well as after a polyA tail has been added (Pandya-Jones and Black 2009) and both polyadenylation (Pandya-Jones et al. 2013) and m^6^A methylation are apparently completed on CA-RNA, it would appear that completion of splicing could be the limiting step in “release.” Early work in mouse erythroleukemia cells showed that β-globin chains mutant in either splice sites or polyA sites never leave the chromatin (Custódio et al. 1999). Thus, effective processing is required to release RNA from the site of transcription.

At least one other potentially time-consuming step to assure mRNA quality control and *T*_1/2_ in the cytoplasm is binding of exon junction complexes (EJCs) (Le Hir et al. 2000, 2001; Maquat and Carmichael 2001). An antibody study of the presence or absence of EJCs on CA-RNA in comparison with nucleoplasmic RNA would be most helpful in this regard. A related idea is that the results of m^6^A addition point to the possibility that recruitment of the 800-kDa complex (Bokar et al. 1994) and methyltransferases to nascent pre-mRNA is similar to the proposed recruitment of splicing machinery and exon definition within pre-mRNA. This possibility must be assessed biochemically.

### m^6^A and mRNA *T*_1/2_

Our present experiments comparing normal mouse embryonic cells and those with no Mettl3 reveal a clear picture that at least many of the site-specific m^6^As in the CDS and the 3′ UTR function in governing mRNA *T*_1/2_. Identification of specific m^6^As that trigger specific mRNA turnover is the key to studying at least the biochemistry of proteins/protein complexes involved in the turnover of specific m^6^A-containing mRNAs. To take one evident case: mRNAs with short *T*_1/2_s can have easily ≥10 m^6^A peaks (clusters). Do these multiple m^6^As act in concert? Furthermore, since ∼20% of mRNAs with no m^6^As do still turn over more rapidly than others without m^6^A, what is the trigger for their turnover?

To begin to analyze specific m^6^As and mRNA *T*_1/2_, we linked the CDS (GFP) in-frame to mouse ESC segments of two different mRNA regions rich in m^6^A. We used both wild-type and mutated sites that removed the normally methylated A. This deletion produced composite mRNAs with slower *T*_1/2_s in the mutants than constructs containing the wild-type sequences (Fig. 7D–K). It was true, however, that the *T*_1/2_ difference in the mutant construct did not affect *T*_1/2_ equally with the same wild-type sequences (Fig. 7D–K).

In other recent experiments, m^6^A-directed RNA binding to specific protein domains of the YTHDF family, which do bind m^6^A-containing oligonucleotides, was at first thought to be involved in mRNA functions such as translation or splicing (Zhang et al. 2010). The m^6^A methylated RNA binding to a motif of YTHDF2 was first used by Dominissini et al. (2012). The YTHDF2 RNA-binding protein was then used to collect m^6^A-containing mRNAs for study (Wang et al. 2014a). An siRNA knockdown of this protein resulted in ∼20%–30% slower turnover for the RNAs that bound most avidly to YTHDF2. A following study also concluded, based on knockdown experiments of the YTHDF1 protein, that “translation efficiency” was dependent on m^6^As in mRNA (Wang et al. 2015). On the basis of these results, summary reviews were published proposing that general roles for m^6^A had been established in mRNA turnover and mRNA translational regulation (Wang and He 2014) and that addition and removal of m^6^A residues were “dynamic” (Fu et al. 2014). A very recent work reiterated the idea that m^6^A in mRNA is subject to a “dynamic” cycle (Cao et al. 2016). Such a conclusion implies removal and addition in the cytoplasm of m^6^A at specific sites. However, as noted above, our results present a strong challenge to this idea of a dynamic cycle that involves a large fraction of the mRNAs in the cells that we examined. Without a doubt, there is a physiologic effect of demethylases (Zheng et al. 2013), but the notion of easy frequent reversibility of m^6^A addition/subtraction, at least in steady-state tissue culture cells, is no longer logical. The m^6^A methylation occurs to nascent pre-mRNA, and the present data argue that it is largely stable in the cytoplasm until the mRNA turnover. If it were to occur, our data indicate that cycles of addition and turnover would have to be targeted to the same sequences within the mature mRNA, for which we found no evidence. Furthermore, it remains possible that methylation removal by demethylases might occur only within the nucleus on CA-RNA transitioning into the nucleoplasm (see Fig. 2A, where specific m^6^A residues in CA-RNA were removed during the release to the nucleoplasm before exit to the cytoplasm.).

### Other specific functions of m^6^A in mRNA

We recently found that m^6^A density is maximal early in the 3′ UTR, and at least some of these m^6^As appear to block use of more upstream polyA sites, thereby resulting in utilization of downstream polyA sites (Ke et al. 2015). In all likelihood, the large number of m^6^A sites in the CDSs and 3′ UTRs might affect a variety of functions, including mRNA stability.

A recent breakthrough has clearly established a function of m^6^A addition to some specific sites near the 5′ end of the mRNA. Eukaryotic initiation factor EIF3 can bind m^6^A that has been added in the 5′ UTRs of mRNA and thereby promote non-cap-mediated translation initiation (Meyer et al. 2015; Mitchell and Parker 2015; Zhou et al. 2015). Evidence of whether this 5′ UTR m^6^A addition on “stress” mRNAs occurs in the nucleus on pre-mRNA on the way to produce a new mRNA or whether the methylation occurs in the cytoplasm to pre-existing mRNA was not provided. The examination of m^6^As in nascent CA-RNA for these particular mRNAs under stress conditions is now strongly suggested.

Finally, the existence of m^6^A in A residues in the 5′ cap of mRNA (m^6^A_m_) was first reported in the 1970s (Salditt-Georgieff et al. 1976; Keith et al. 1978). When the first nucleotide in a pre-mRNA is an A residue, many such A residues acquire m^6^A_m_ modification. A recent finding indicates that such mRNAs are generally long-lived and are demethylated by the FTO demethylase (Mauer et al. 2017). Overexpression of FTO causes a faster turnover of these mRNAs, and an FTO knockout stabilizes these same mRNAs. As capping occurs shortly after pre-mRNA initiation by enzymes known to act in the nucleus (Venkatesan and Moss 1982), these results almost certainly mean that m^6^A_m_ modification occurs at least initially in CA-RNA. These results also provide a connection between m^6^A_m_ modification and subsequent effects on mRNA *T*_1/2_ but suggest that a different mechanism is at play when the 5′ end of the transcript is capped and methylated compared with internal m^6^A modification.

In summary, use of the widely available highly specific m^6^A antisera plus the availability of low-cost sequencing and now the application of an updated cell fractionation protocol have quickly advanced our knowledge of a decades-old problem of m^6^A—the site-specific location and the function of m^6^A in mRNA. We now know that the specific sites found in mRNA are methylated on the nascent pre-mRNA and can link this methylation to at least several specific functions of some of the m^6^A residues in eukaryotic mRNA.

## Materials and methods

### Fractionation into CA-RNA, nucleoplasm, and cytoplasm RNA groups

HeLa cells were grown to 90% confluency in DMEM (Life Technologies) supplemented with 10% fetal bovine serum (Omega), 2 mM L-glutamine (Life Technologies), 1 U/mL penicillin/streptomycin (Life Technologies), 1× nonessential amino acids (Life Technologies), and 0.1 mM β-mercaptoethanol (Sigma). Fractionations were performed as described previously (Pandya-Jones and Black 2009). Briefly, cell pellets from a 90% confluent 10-cm plate were gently scraped and collected in PBS at room temperature (4000 rpm for 1 min to pellet). Pellets were lysed in 200 µL of ice-cold cytoplasmic lysis buffer for 5 min on ice. Cellular lysates were passed through 500 µL of an ice-cold sucrose cushion at 10,000 rpm for 10 min at 4°C. The ∼700-µL cytoplasmic fraction was removed and used for protein analysis or further processed to obtain RNA samples (Pandya-Jones and Black 2009). Pelleted nuclei were rinsed gently with 200 µL of ice-cold PBS and then resuspended in 100 µL of ice-cold glycerol buffer prior to being lysed with 100 µL of ice-cold nuclear lysis buffer. After vortexing twice for 2 sec, nuclear lysates were incubated on ice for 2 min and then pelleted at 14,000 rpm for 2 min at 4°C. The soluble nuclear fraction was removed and used for protein analysis or further processed to obtain RNA samples (Pandya-Jones and Black 2009). The chromatin pellet was rinsed gently with 200 µL of ice-cold PBS and then digested in 50 µL of 1× DNase I buffer with 2 U of Turbo DNase (Thermo Fisher) for 10 min at 37°C. Protein samples were removed before dissolving the digested chromatin in 1 mL of TRIzol (Life Technologies) for RNA isolation. All centrifugations were conducted in Eppendorf 5424 centrifuges at either room temperature or 4°C. All solutions were supplemented with 1× Complete protease inhibitors (Roche Life Sciences). In total, we prepared three biological replicates of cell fractionation into chromatin, nucleoplasm, and cytoplasm. Both RNA-seq and m^6^A mapping analysis are highly reproducible among biological replicates, and Figure 1 integrates the result of three biological replicates.

We also included in the Supplemental Material a protocol for m^6^A-CLIP (Ke et al. 2015) with a detailed day-to-day arrangement of experiments.

### Determination of m^6^A peak regions in exon vs. intron

To rigorously classify m^6^A peaks into exonic versus intronic regions, we used the complete set of transcript annotations according to GENCODE (version 19 of human hg19). If an m^6^A peak was located at any known exonic region, it was classified as an exonic m^6^A peak. If it was located only at an intronic region of transcripts, it was classified as an intronic m^6^A peak. Using alternative annotations (e.g., Ensembl 85 version 25 for human GRCh38) generated essentially the same result. To examine the distribution of m^6^A peaks in partially spliced mRNAs (Fig. 1B,C), we focused on pre-mRNAs with abundant intronic RNA reads (i.e., there were two or more times more intronic RNA reads than exonic RNA reads). Using data for all pre-mRNAs, including those in which internal exons were completely or largely spliced (no or few introns), we obtained the same result: m^6^As in CA-RNAs are >93% in exons (see Fig. 1B).

### Distribution of m^6^A peak regions around start codons, stop codons, and the start of last exons

To unambiguously assign m^6^A peak regions to mRNAs according to gene annotations, we used a subset of GENCODE annotations (version 19 for human hg19 and version M10 for mouse mm10) by taking only one transcript isoform for each mRNA: We used the isoform with the longest mRNA length (alternatively, we used the isoform with the most distal 3′ end, which generated essentially the same result). We then removed overlapping transcripts from the set to avoid any ambiguity in determining which transcript the m^6^A peak region was from.

We considered m^6^A peak regions that were within 1-kb mRNA distance from stop codons and the fact that mRNAs had different lengths. We generated 100 intervals, each with a 10-nt size for 1 kb upstream of and downstream from the stop codons. We computed the “m^6^A peak region density” (i.e., “m^6^A peak density”) for each interval as follows: We scanned through all mRNAs of interest that contained this interval and examined whether an m^6^A peak region existed in this interval. We then enumerated those cases that contained m^6^A peak regions and divided this value by the total number of mRNAs that contained this interval. For the plot of m^6^A peak region density around the start of last exons and start codons, we performed the same analysis except anchoring at the start of the last exons and start codons.

### Detection of pre-mRNA reads that have both intron and m^6^A-containing exon sequences

To identify pre-mRNA reads that have both intron and m^6^A-containing exon sequences, we focused on precisely mapped m^6^A sites that were within 80 nt of splice sites (the length of our m^6^A sequencing reads was ∼80 nt). We required the read length in the intronic region to be at least 4 nt for these pre-mRNA reads. Furthermore, we also required the number of pre-mRNA reads in each case for CA-RNA be more than two as the reliable evidence of existence. In addition, these pre-mRNA reads should not exist in nucleoplasm and cytoplasm RNAs. We identified >200 internal exons to have m^6^A-containing exon–intron junction fragments (six total examples in Fig. 3B,C and Supplemental Fig. 5; full list in Supplemental Table 1).

### Investigating m^6^A locations relative to splice sites

To clearly show the distribution of m^6^A peaks relative to splice sites, we focused on internal exons with exon length at least 200 nt so that the 100-nt exon regions from 5′ splice sites and 3′ splice sites would not overlap (Fig. 4A,C). The internal exons that are at least 200 nt long contained ∼80% of all internal exon m^6^As. “Relative m^6^A peak density” for a fixed position relative to a splice site (Fig. 4A,C) was calculated as the m^6^A peak density at that position scaled in proportion to the average m^6^A peak density in exonic regions at least 100 nt away from the splice sites. Figure 4B shows that ∼7% of exonic m^6^As are within 50 nt of splice sites for internal exons in Figure 4A (i.e., at least 200 nt long). If we consider all m^6^A-containing internal exons, including those exons >100 nt long in which all m^6^As are automatically within 50 nt distance from splice sites, 20% of exonic m^6^As are within 50 nt of splice sites.

### Determination of m^6^A peaks that are higher in CA-RNA

To determine m^6^A peaks that are higher in CA-RNA, for each m^6^A peak region, we enumerated reads of m^6^A immunoprecipitation and the input for CA-RNA and nucleoplasm RNA to evaluate the statistical significance (Fisher's exact test). Benjamini-Hochberg was implemented to adjust the *P*-value to the FDR for multiple testing. The requirement that an m^6^A peak region is higher in CA-RNA included (1) that the reads of mRNAs in m^6^A peak regions were adequate for m^6^A peak region detection in both CA-RNA and nucleoplasmic mRNA (reads per kilobase per million mapped reads [RPKM] ≥1) and (2) that the m^6^A peak regions that are higher in CA-RNA were determined by requiring FDR ≤0.05 and an at least twofold higher peak region enrichment in CA-RNA compared with nucleoplasmic mRNA. At a lower cutoff (e.g., ≥1.5 fold), the same conclusion held: Most m^6^A peaks are modified with the same level between CA-RNA and nucleoplasmic mRNA. Comparison of individual m^6^A peak signal strength in nucleoplasmic RNA and cytoplasmic RNA for the same m^6^A peak was performed in the same way as the comparison between CA-RNA and nucleoplasmic RNA.

### Determination of mRNA *T*_1/2_

We determined the *T*_1/2_s of individual polyA^+^ mRNAs in mouse ESCs and Mettl3 knockout by sequencing after five time points (0, 1, 2, 4, and 8 h) of actinomycin (final concentration of 5 µg/mL; Sigma, no. A9415) treatment in three biological replicates. The *T*_1/2_ was determined as *ln*(2)/*k*, where *k* is the decay rate constant. The individual mRNA abundance levels at different time points after actinomycin D treatment were fitted to a first-order exponential decay curve to calculate the decay rate constant (*k*). A single *T*_1/2_ was calculated for each mRNA by using all three replicate values at each time point. For downstream detailed analysis, we considered only the *T*_1/2_s for individual mRNAs with reliable statistics (i.e., FDR <0.05, fitting the exponential decay model by *t* distribution) (Wang et al. 2002; Neff et al. 2012). We used the published mRNA *T*_1/2_ data for HeLa cells (Tani et al. 2012) to overlay HeLa cell m^6^A data with mRNA *T*_1/2_ (Fig. 5A). Using the mRNA *T*_1/2_ data in human hepatoma cancer cells from an independent group (Yang et al. 2003), we obtained similar associations of more m^6^As in mRNAs and shorter *T*_1/2_s (Supplemental Fig. 10).

The m^6^A minigene for mRNA half-life validation was constructed based on a common retroviral GFP vector (Addgene, no. 1764); puromycin was the selection marker for successful DNA integration. The mRNA regions containing m^6^A sites were cloned in-frame into the minigene at multiple clone sites that were at the end of the coding region of GFP. Synonymous point mutations were carefully made to disrupt the m^6^A RAC core motif while not changing the underlying protein-coding sequence.

The detailed sequences for the Ppp1r8 and Sox2 constructs (wild type and mutant) are in the Supplemental Material.

### Determination of m^6^A peak regions lost in knockout of Mettl3 in mouse ESCs

Global quantification of m^6^A by mass spectrometry was practiced according to a protocol described previously (Ke et al. 2015). To determine m^6^A peak regions that were lost due to Mettl3 knockout in mouse ESCs, we practiced the analysis as reported previously (Ke et al. 2015). For each m^6^A peak region, we enumerated reads of m^6^A immunoprecipitation and the input for wild-type and knockout mouse ESCs to evaluate the statistical significance (Fisher's exact test). Benjamini-Hochberg was implemented to adjust the *P*-value to the FDR for multiple testing. The requirement that an m^6^A peak region is considered lost due to Mettl3 knockout demanded (1) that the mRNAs containing these m^6^A peak regions should be adequately expressed in both wild-type and knockout mouse ESCs (RPKM ≥1), (2) that the expression of mRNAs in m^6^A peak regions was adequate for m^6^A peak region detection in both knockout and wild type (RPKM ≥1), and (3) an FDR ≤0.05 and an at least twofold decrease of peak region enrichment in knockout compared with wild type.

## Supplementary Material

Supplemental Material
