# Supplementary material for: m6A mRNA modifications are deposited in nascent pre-mRNA and are not required for splicing but do specify cytoplasmic turnover
Source: Genes Dev. 2017 May 15;31(10):990–1006. doi: 10.1101/gad.301036.117 (PMC5495127; doi:10.1101/gad.301036.117)
Supplement: Supplemental Material [file supp_31_10_990__index.html]

Supplemental Material 

# m6A mRNA modifications are deposited in nascent pre-mRNA and are not required for splicing but do specify cytoplasmic turnover

## Supplemental Material

- SupplementalMaterialandMethods.pdf
