# Supplementary material for: m6A mRNA modifications are deposited in nascent pre-mRNA and are not required for splicing but do specify cytoplasmic turnover
Source: Genes Dev. 2017 May 15;31(10):990–1006. doi: 10.1101/gad.301036.117 (PMC5495127; doi:10.1101/gad.301036.117)
Supplement: Supplemental Material [file supp_31.10.990_SupplementalMaterialandMethods.pdf]

Supplemental Material For Ke et al. *Genes & Development* 2017

m^{6}A mRNA modifications are deposited in nascent pre-mRNA and are not required for splicing but do specify cytoplasmic turnover

Shengdong Ke[1], Amy Pandya-Jones[2], Yuhki Saito[1], John J. Fak[1], Cathrine Broberg Vågbø[3], Shay Geula[4], Jacob H. Hanna[4], Douglas L. Black[2], James E. Darnell, Jr.[5,6], Robert B. Darnell[1,6]

[6]Correspondences:
Email: darnelr@rockefeller.edu
Email: darnell@rockefeller.edu

A. Legend to Supplemental Figures (Figure S1 to S12) and Supplemental Tables (Table S1 to S5)
B. Supplemental Figure S1 to S12
C. Supplemental Table S1 to S5
D. Supplemental Methods and Materials
E. A protocol for m^{6}A-CLIP (Ke et al. *Genes & Development* 2015) with a detailed arrangement of experiments into different days

Legend to Supplemental Figures and Tables

Supplemental Fig 1. Many exons are not yet spliced in CA-RNAs according to intron splicing completion index (see also Fig1A which uses internal exon splicing completion index).

Supplemental Fig 2. . Both exonic and intronic m^6A peaks in all three RNA fractions identified RRACU consensus motif. Intronic m^6A peaks in CA-RNA contain both its unique intronic m^6A peaks and those conserved in other fractions. These nucleoplasmic and cytoplasmic "intronic m^6As" would not be likely in real introns. They are probably exonic m^6As, same as the rest 98% of m^6As (current reference annotations are yet to be complete, and the existence of these "intronic m^6As" in Cytoplasmic polyA+ RNAs rules out the ce-RNA possibility). Data analysis in Figure 4C and Supplemental Figure 5 has comprehensively summarized all m^6As statistically (including both exonic m^6As and these so called "intronic m^6As"). The actual number of 4% CA-RNA only intron m^6A is 937 (the number for the 3% "intron m^6As" conserved in other fractions is 657). The great preference of m^6A to exons is evident but imperfect as there are still few intronic m^6As in CA-RNA.

Supplemental Fig 3. m^6A peaks have the same distribution in RNAs from the three cell fractions (anchoring at stop codon). Black line represents mRNAs with stop codon in last exon; red line represents mRNAs with stop codon not in last exon. The shade region (grey and light pink) is the standard error of the mean (S.E.M.). (Not only distribution is the same but also the m^6A peak strength for individual m6A peaks, see Fig. 2)

Supplemental Fig 4. Exon m^6A addition to nascent pre-mRNA is independent of its

distance to polyA sites.

A) Internal exons close to polyA sites in CA-RNA are less completely spliced (See details in Supplemental Materials and Methods). ***, $p < 10^{-15}$, Wilcox ranked test.

B) A similar frequency (~4%) of m^6A occurs to internal exons of CA-RNA regardless of the distance to the polyA site (n.s. stands for not significant).

Supplemental Fig 5. m^6A can be added to exons before splicing. These are additional examples to the two shown in Fig 3A and B. "m^6A-CLIP site" shows a precise m^6A site (black box) identified by m^6A-CLIP. "IP reads" lists the cDNA reads of RNA fragments that were precipitated by m^6A specific antibody and contain both the m^6A site and the unspliced intronic region.

Supplemental Fig 6. CA-RNA higher m^6As are relatively more favored in internal exons, most of which are constitutive exons.

Supplemental Fig 7. The majority of m^6As do not locate close to splice site (alternative exons). Density of m^6A at increasing distance from 3' or 5' splice sites in CA-RNA (orange lines), nucleoplasm (dark blue) and cytoplasm (light blue). "relative m^6A peak density" for a fixed position to splice site is calculated as the scaled m^6A peak density at that position scaled proportional to the average m^6A peak density in exonic regions >100nt away from splice sites (black line). To clearly show the distribution of m^6A peaks to splice sites, we focus on internal exons with exon length at least 200nt so that the 100nt exon regions from 5'SS and 3'SS do not overlap. As the major form of alternative splicing, alternative cassette exons are studied here. Error bar is the S.E.M.. A) Alternative cassette exons in HeLa cell (the center exon is required to have m^6A, total exon number: 1072); B) Alternative cassette exons in mouse ES cell (the center exon is

required to have m^6A, total exon number: 313). C) Alternative cassette exons using Yang group's raw data could still not reproduce the sharp enrichment of m^6A at splice sites. (See also Supplemental Figure 8)

Supplemental Fig 8. The majority of m^6As do not locate close to splice site, reanalysis of the raw sequencing data from different labs. A) Our own m^6A mapping data, and the top enriched motif in m^6A peaks is RRACU; B) Reanalysis of the raw sequencing data of Zhao et al 2014 (Yang Lab), and the top enriched motif in m^6A peaks is not RRACU; C) Reanalysis of the raw sequencing data of Schwart et al 2014 (Regev lab), and the top enriched motif in m6A peaks is RRACU. D) Reanalysis of the raw sequencing data of Meyer et al 2012 (Jaffrey lab), and the top enriched motif in m6A peaks is RRACU.

Supplemental Fig 9. Internal exons containing m^6As splices mostly the same upon different level of global m^6A loss by compromising Mettl3 expression level, using raw RNAseq data of several published papers that reported certain splicing changes upon compromising Mettl3 expression level. Internal constitutive exons with m^6A are defined as the tri-exon structure with constitutive exon being the center exon, and we require <u>at least one of the three exons</u> should have m^6A. (Significant changes were defined as delta PSI >=0.1 and FDR<5%). Internal alternative cassette exons with m6A are defined as the tri-exon structure with alternative cassette exon being the center exon, and we require at least one of the three exons should have m^6A. (Significant changes were defined as delta PSI>=0.1 and FDR<5%). Other alternative splicing types showed even less changes including alternative 5' splice site, alternative 3' splice site and intron retention. A) using the raw RNAseq data from Dominissini et al Nature 2012 (Dominissini et al., 2012); B) using raw RNAseq data from Zhao et al Cell Research 2014 (Zhao et al., 2014); C) using

raw RNAseq data from Guela et al Science 2015 (Geula et al., 2015); D) using raw RNAseq data from Liu et al Nature 2015 (Liu et al., 2015).

Supplemental Fig 10. mRNAs with shorter half-life have higher m^6A density. A) HeLa cell (anchoring at last exon start, using $T_{1/2}$ of Tani *et al* 2012 (Tani et al., 2012), see also Fig. 5A for anchoring at stop codon); B) HeLa cell (using $T_{1/2}$ from the other group, Yang *et al* 2002 (Yang et al., 2003)); C) Mouse ES cell (anchoring at last exon start, see also Fig. 5B for anchoring at stop codon)

Supplemental Fig 11. Identifying Mettl3-dependent m^6A peaks (i.e. WT higher) by comparison of WT mRNA to Mettl3 KO RNA for m^6A signal strength (no changes, grey; WT higher, red; KO higher, dark blue; Significant changes require fold of change>=2 and FDR<5%, Fisher's exact test)

Supplemental Fig 12. Co-IP between m6A methyltransferase (METTL3 and METTL14), U1 snRNP and U2AF proteins involved in exon definition (U1A and U2AF65). As control, RBFOX2 can co-IP hnRNP M but not Pol II (Damianov et al., 2016). Endogenous protein co-IP was performed on a high molecular weight (HMW) nuclear fraction that was released from chromatin and preserved some protein-protein interaction intact (Damianov et al., 2016). Detailed antibody information is provided in Supplemental Material and Methods.

Supplemental Table 1. List of m^6A sites that are nearby splice sites and yield m^6A-containing exon-intron junction fragments in nascent RNA

Supplemental Table 2. List of several SR proteins binding site information (could be bound by SR proteins and act as ESEs, motifs are highly degenerate)

Supplemental Table 3. List of Gene Ontologies (GOs) that are significantly enriched in short $T_{1/2}$ mRNAs with multiple m^6As (>=2), and most GOs are associated with regulatory functions.

Supplemental Table 4. List of Gene Ontologies (GOs) that are significantly enriched in long $T_{1/2}$ mRNAs with no m^6A, and most GOs are associated with house keeping functions.

Supplemental Table 5. List of Gene Ontologies (GOs) that are significantly enriched in short $T_{1/2}$ mRNAs with no m^6A, and most GOs are associated with nucleosome related functions.

Reference:

Damianov A, Ying Y, Lin CH, Lee JA, Tran D, Vashisht AA, Bahrami-Samani E, Xing Y, Martin KC, Wohlschlegel JA, Black DL. 2016. Rbfox Proteins Regulate Splicing as Part of a Large Multiprotein Complex LASR. *Cell* **165**: 606–619.

Dominissini D, Moshitch-Moshkovitz S, Schwartz S, Salmon-Divon M, Ungar L, Osenberg S, Cesarkas K, Jacob-Hirsch J, Amariglio N, Kupiec M, Sorek R, Rechavi G. 2012. Topology of the human and mouse m6A RNA methylomes revealed by m6A-seq. *Nature* **485**: 201–206.

Geula S, Moshitch-Moshkovitz S, Dominissini D, Mansour AA, Kol N, Salmon-Divon M, Hershkovitz V, Peer E, Mor N, Manor YS, Ben-Haim MS, Eyal E, Yunger S, Pinto Y, Jaitin DA, Viukov S, Rais Y, Krupalnik V, Chomsky E, Zerbib M, Maza I, Rechavi Y, Massarwa R, Hanna S, Amit I, Levanon EY, Amariglio N, Stern-Ginossar N, Novershtern N, Rechavi G, Hanna JH. 2015. Stem cells. m6A mRNA methylation facilitates resolution of naive pluripotency toward differentiation. *Science* **347**: 1002–1006.

Liu N, Dai Q, Zheng G, He C, Parisien M, Pan T. 2015. N(6)-methyladenosine-dependent RNA structural switches regulate RNA-protein interactions. *Nature* **518**: 560–564.

Tani H, Mizutani R, Salam KA, Tano K, Ijiri K, Wakamatsu A, Isogai T, Suzuki Y, Akimitsu N. 2012. Genome-wide determination of RNA stability reveals hundreds of short-lived noncoding transcripts in mammals. *Genome Res* **22**: 947–956.
Yang E, van Nimwegen E, Zavolan M, Rajewsky N, Schroeder M, Magnasco M, Darnell JEJ. 2003. Decay rates of human mRNAs: correlation with functional characteristics and sequence attributes. *Genome Res* **13**: 1863–1872.
Zhao X, Yang Y, Sun BF, Shi Y, Yang X, Xiao W, Hao YJ, Ping XL, Chen YS, Wang WJ, Jin KX, Wang X, Huang CM, Fu Y, Ge XM, Song SH, Jeong HS, Yanagisawa H, Niu Y, Jia GF, Wu W, Tong WM, Okamoto A, He C, Rendtlew Danielsen JM, Wang XJ, Yang YG. 2014. FTO-dependent demethylation of N6-methyladenosine regulates mRNA splicing and is required for adipogenesis. *Cell Res* **24**: 1403–1419.

FigS1

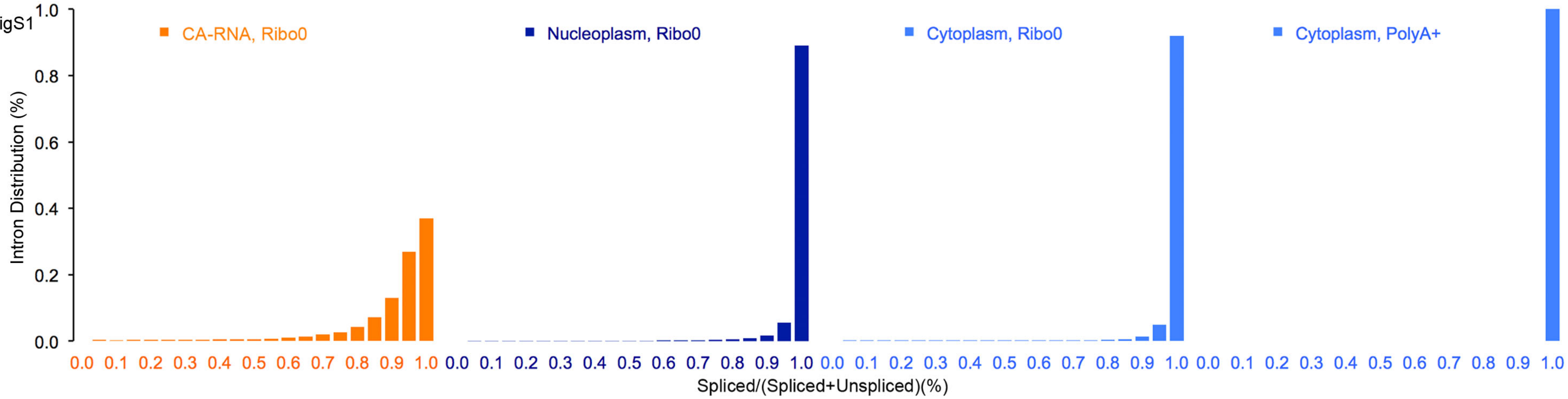

1.0
0.8
0.6
0.4
0.2
0.0
Intron Distribution (%)
CA-RNA, Ribo0
Nucleoplasm, Ribo0
Cytoplasm, Ribo0
Cytoplasm, PolyA+
0.0 0.1 0.2 0.3 0.4 0.5 0.6 0.7 0.8 0.9 1.0 0.0 0.1 0.2 0.3 0.4 0.5 0.6 0.7 0.8 0.9 1.0 0.0 0.1 0.2 0.3 0.4 0.5 0.6 0.7 0.8 0.9 1.0 0.0 0.1 0.2 0.3 0.4 0.5 0.6 0.7 0.8 0.9 1.0
Spliced/(Spliced+Unspliced)(%)

FigS2

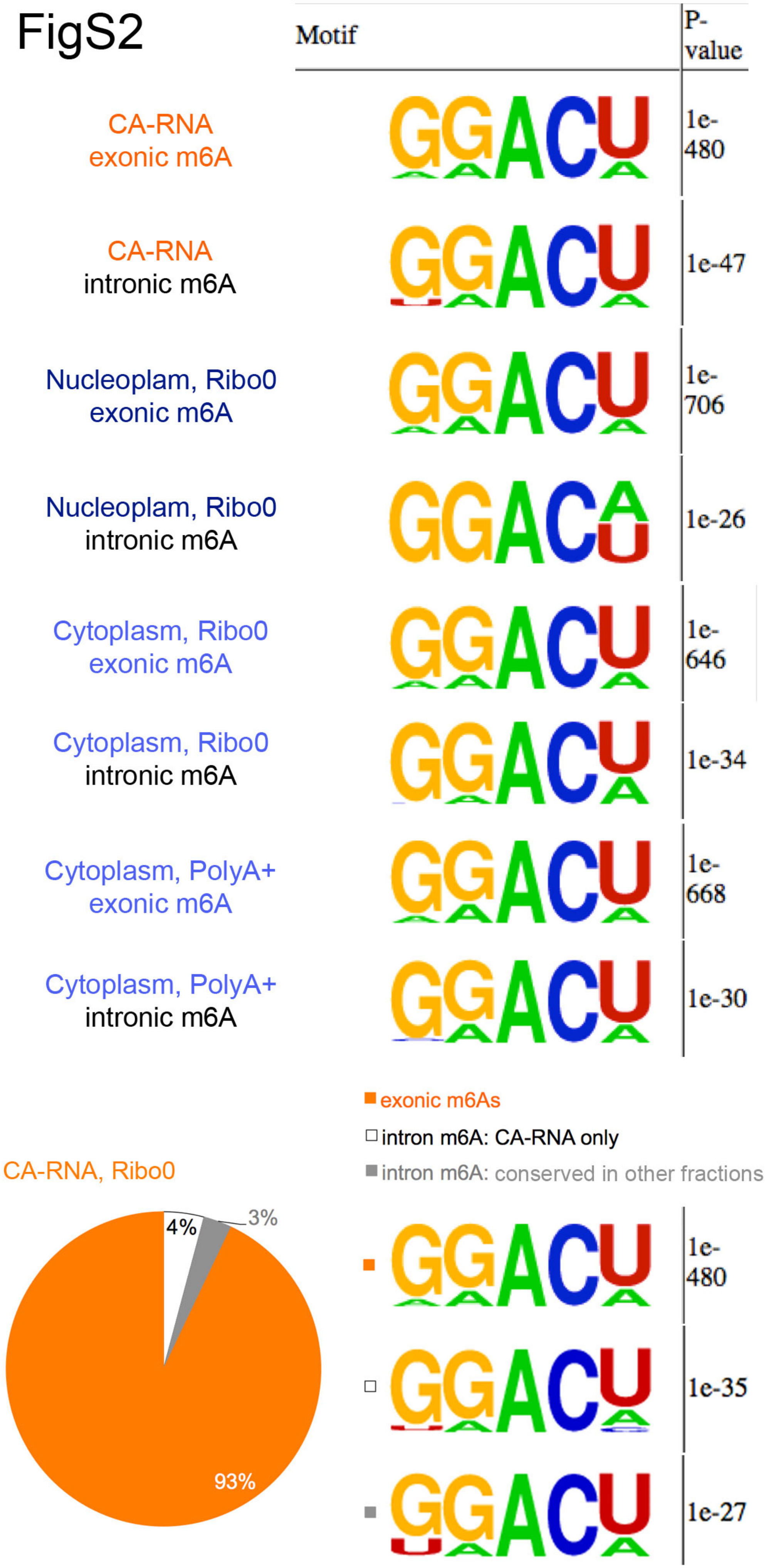

Motif
P-value
CA-RNA exonic m6A
1e-480
CA-RNA intronic m6A
1e-47
Nucleoplam, Ribo0 exonic m6A
1e-706
Nucleoplam, Ribo0 intronic m6A
1e-26
Cytoplasm, Ribo0 exonic m6A
1e-646
Cytoplasm, Ribo0 intronic m6A
1e-34
Cytoplasm, PolyA+ exonic m6A
1e-668
Cytoplasm, PolyA+ intronic m6A
1e-30
exonic m6As
intron m6A: CA-RNA only
intron m6A: conserved in other fractions
CA-RNA, Ribo0
4%
3%
93%
1e-480
1e-35
1e-27

FigS3

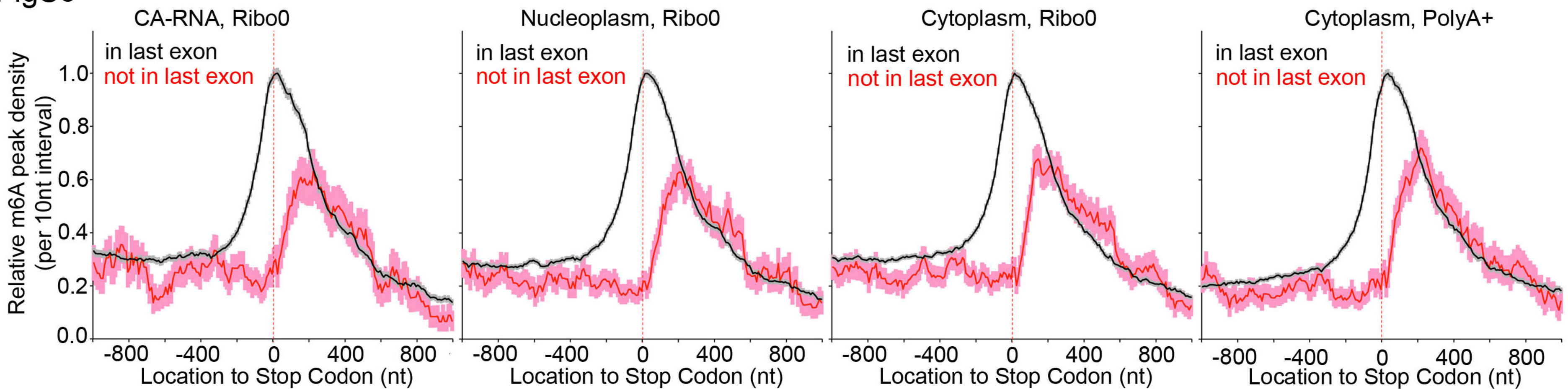

CA-RNA, Ribo0
Nucleoplasm, Ribo0
Cytoplasm, Ribo0
Cytoplasm, PolyA+
in last exon
not in last exon
Relative m6A peak density
(per 10nt interval)
1.0
0.8
0.6
0.4
0.2
0.0
-800
-400
0
400
800
Location to Stop Codon (nt)

FigS4

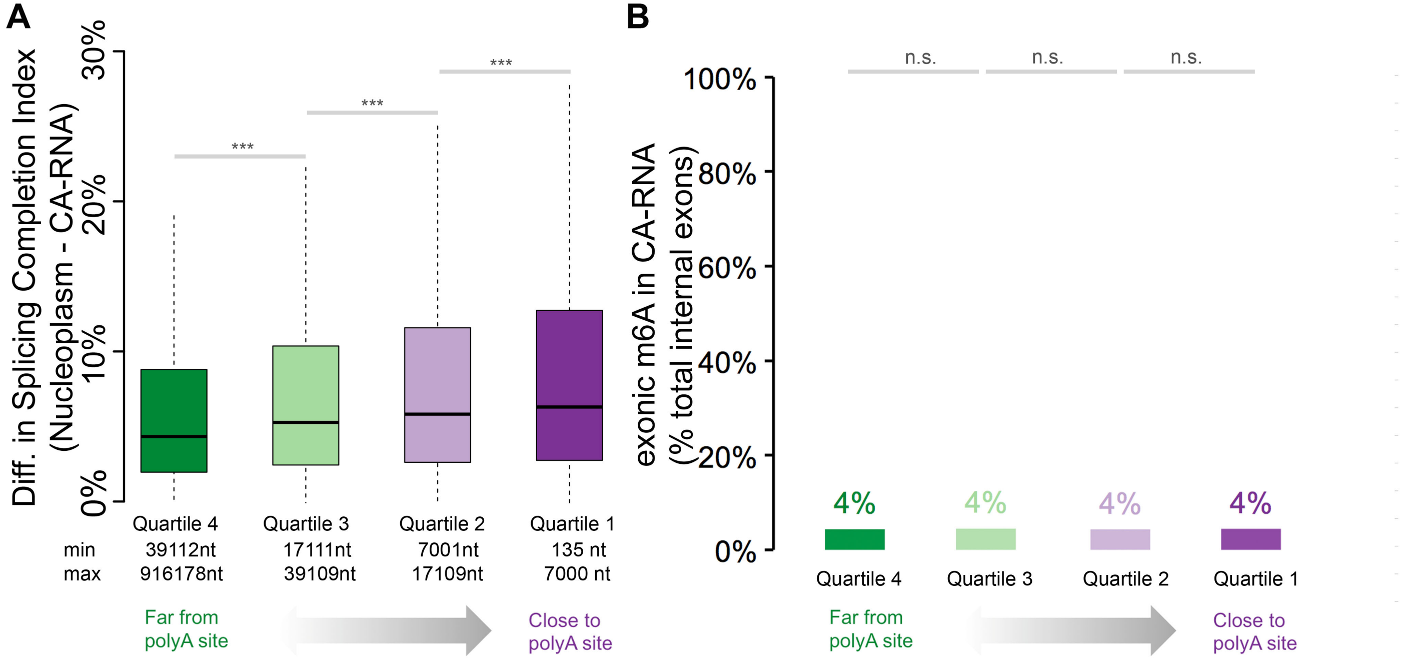
A
Diff. in Splicing Completion Index
(Nucleoplasm - CA-RNA)
0%
10%
20%
30%
***
***
***
Quartile 4
Quartile 3
Quartile 2
Quartile 1
min
max
39112nt
916178nt
17111nt
39109nt
7001nt
17109nt
135 nt
7000 nt
Far from polyA site
Close to polyA site
B
exonic m6A in CA-RNA
(% total internal exons)
0%
20%
40%
60%
80%
100%
n.s.
n.s.
n.s.
4%
4%
4%
4%
Quartile 4
Quartile 3
Quartile 2
Quartile 1
Far from polyA site
Close to polyA site

FigS5

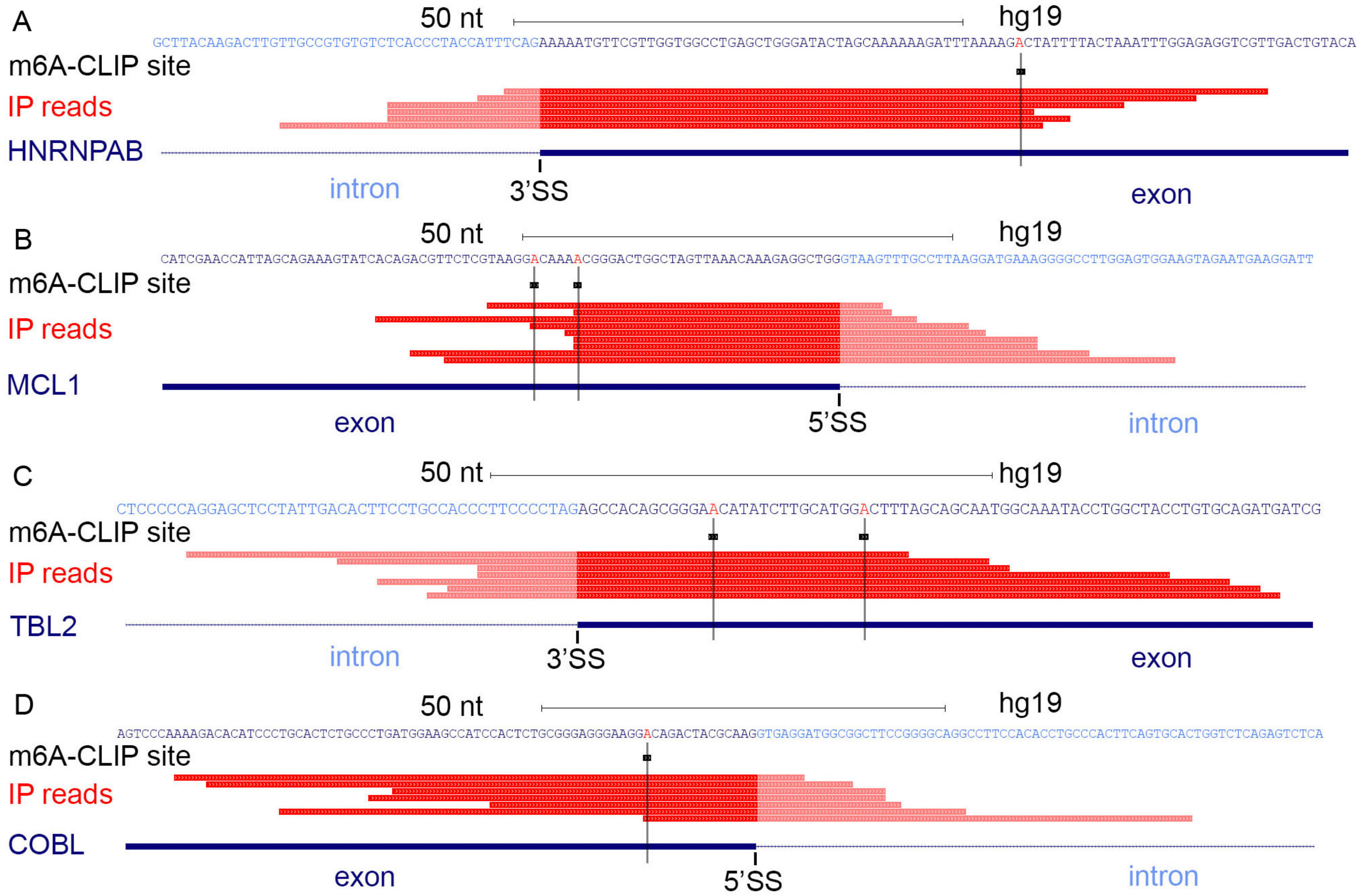

A
50 nt
hg19
m6A-CLIP site
IP reads
HNRNPAB
intron
3'SS
exon
B
50 nt
hg19
m6A-CLIP site
IP reads
MCL1
exon
5'SS
intron
C
50 nt
hg19
m6A-CLIP site
IP reads
TBL2
intron
3'SS
exon
D
50 nt
hg19
m6A-CLIP site
IP reads
COBL
exon
5'SS
intron

FigS6

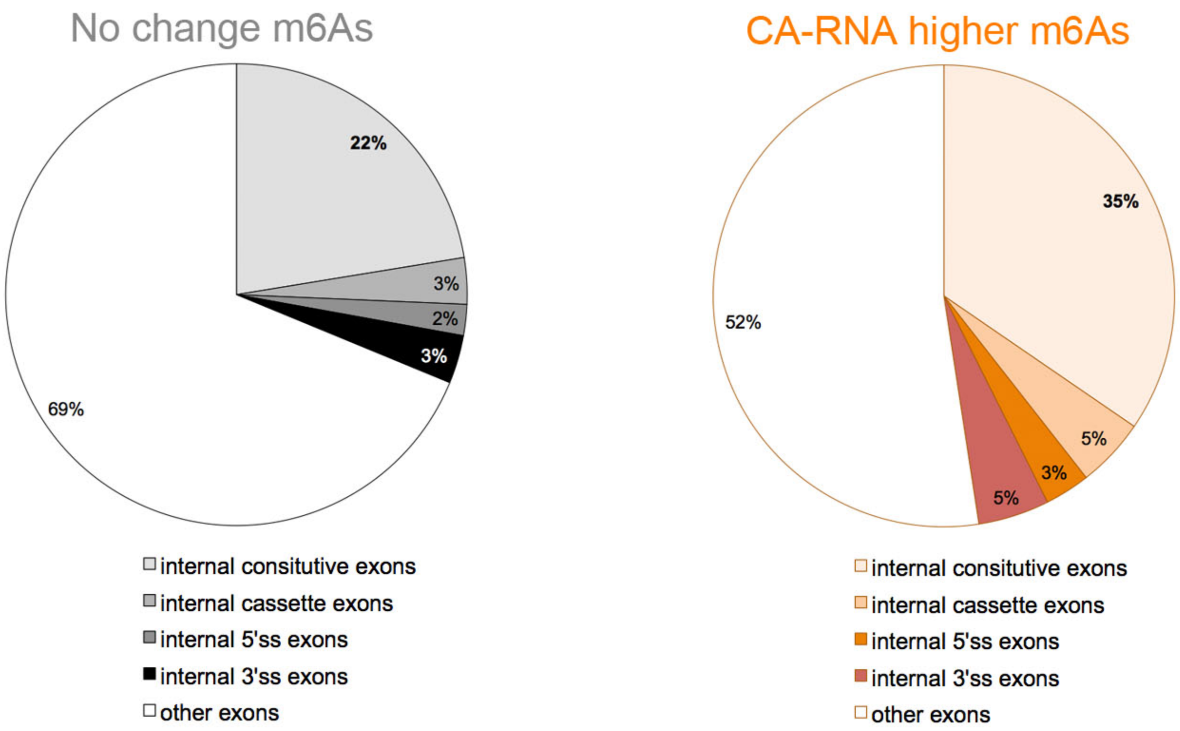

No change m6As
22%
3%
2%
3%
69%
internal consitutive exons
internal cassette exons
internal 5'ss exons
internal 3'ss exons
other exons
CA-RNA higher m6As
35%
52%
5%
3%
5%
internal consitutive exons
internal cassette exons
internal 5'ss exons
internal 3'ss exons
other exons

FigS7

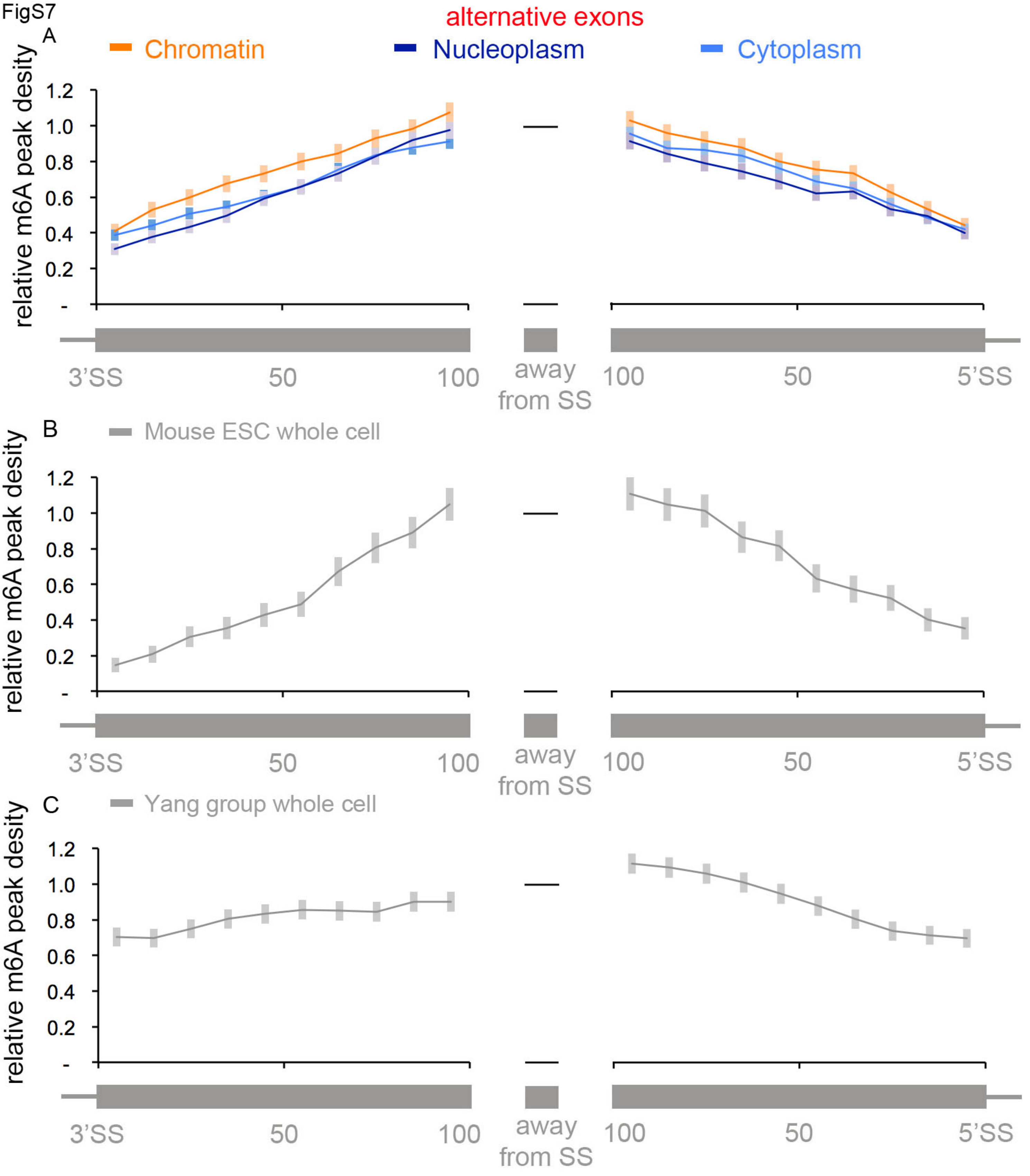
alternative exons
A
Chromatin
Nucleoplasm
Cytoplasm
relative m6A peak desity
1.2
1.0
0.8
0.6
0.4
0.2
-
3'SS
50
100
away from SS
100
50
5'SS
B
Mouse ESC whole cell
relative m6A peak desity
3'SS
50
100
away from SS
100
50
5'SS
C
Yang group whole cell
relative m6A peak desity
3'SS
50
100
away from SS
100
50
5'SS

FigS8

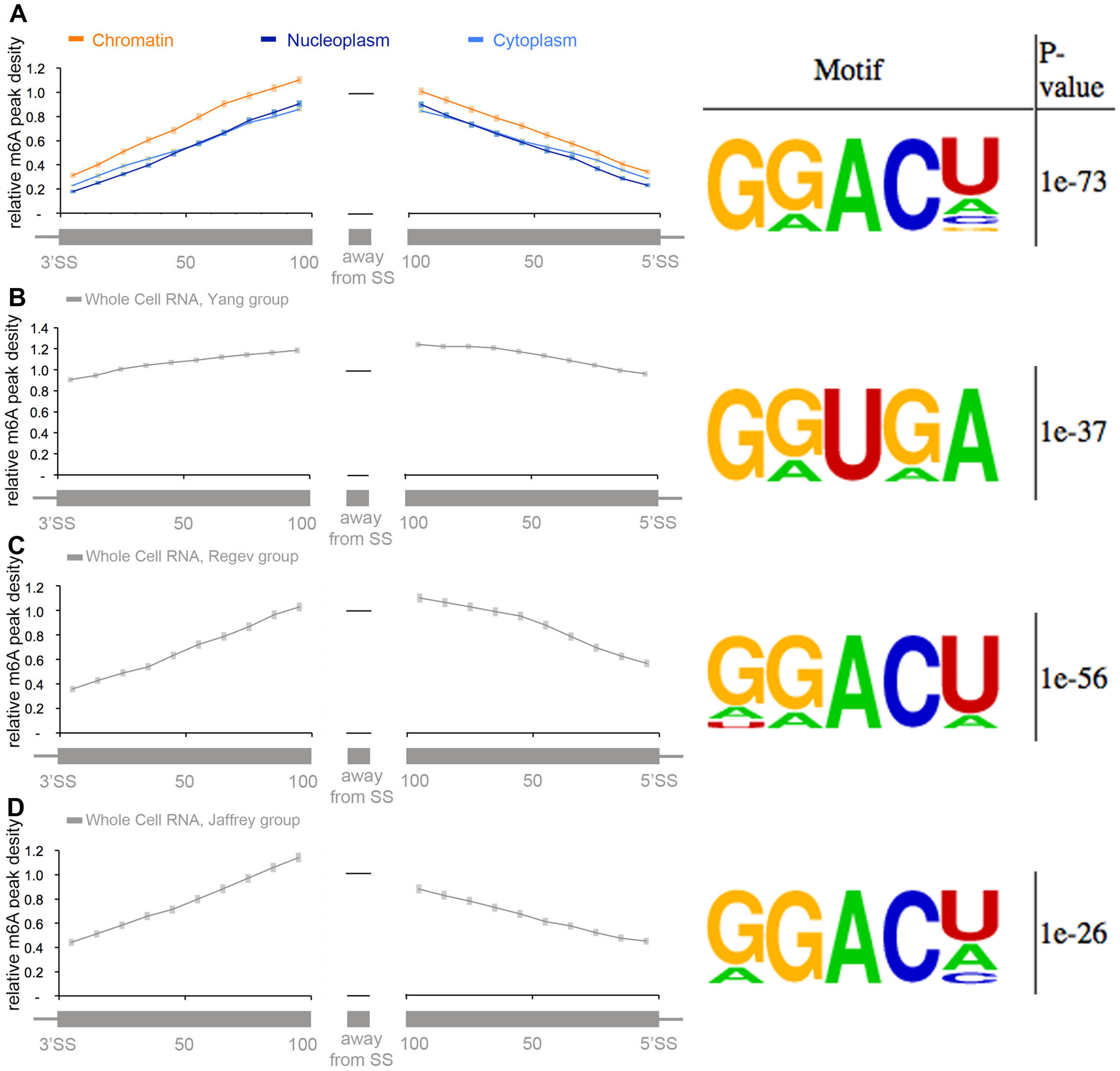
A
Chromatin
Nucleoplasm
Cytoplasm
relative m6A peak desity
3'SS
50
100
away from SS
100
50
5'SS
Motif
P-value
1e-73
B
Whole Cell RNA, Yang group
1e-37
C
Whole Cell RNA, Regev group
1e-56
D
Whole Cell RNA, Jaffrey group
1e-26

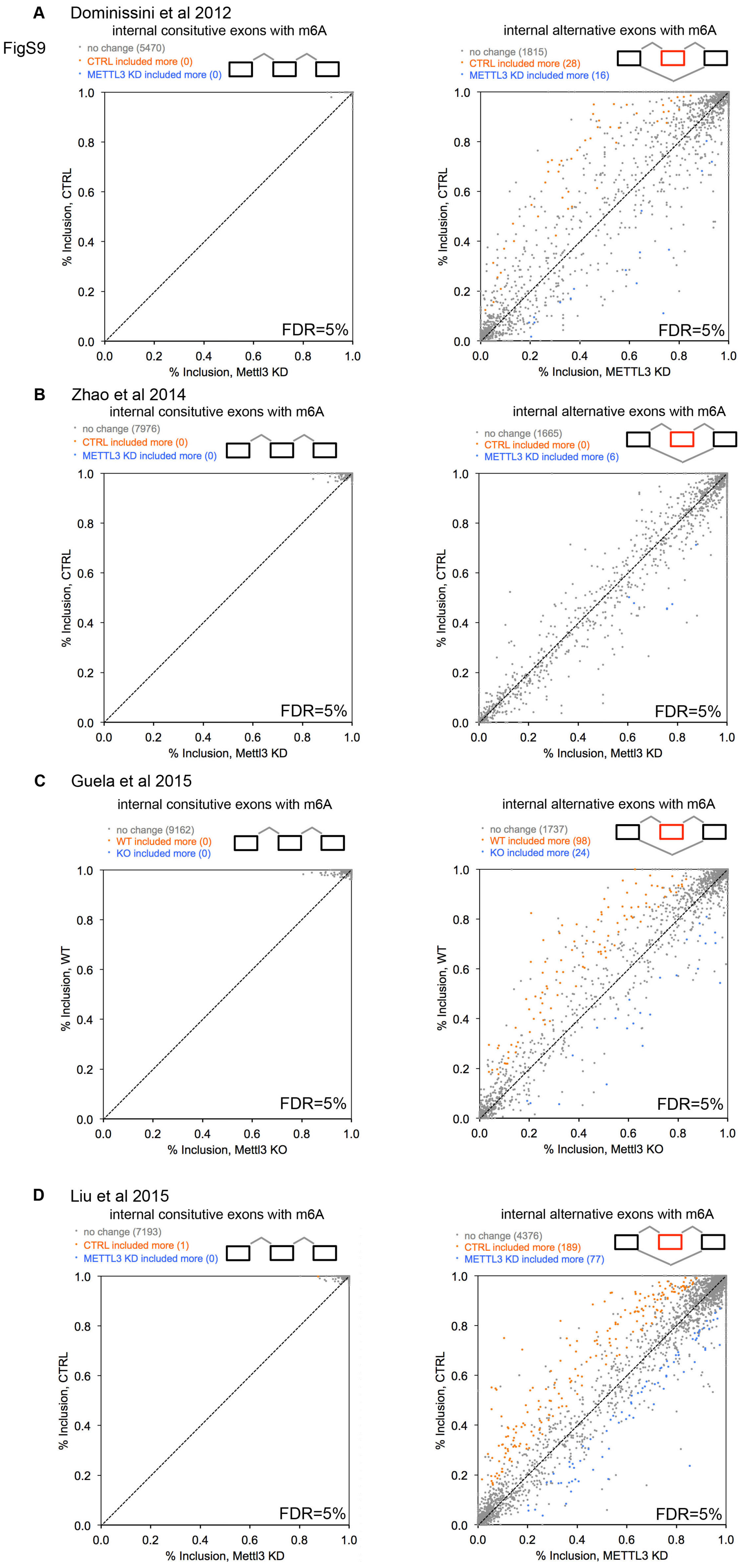
FigS9
A
Dominissini et al 2012
internal consitutive exons with m6A
no change (5470)
CTRL included more (0)
METTL3 KD included more (0)
% Inclusion, CTRL
% Inclusion, Mettl3 KD
FDR=5%
internal alternative exons with m6A
no change (1815)
CTRL included more (28)
METTL3 KD included more (16)
% Inclusion, CTRL
% Inclusion, METTL3 KD
FDR=5%
B
Zhao et al 2014
internal consitutive exons with m6A
no change (7976)
CTRL included more (0)
METTL3 KD included more (0)
% Inclusion, CTRL
% Inclusion, Mettl3 KD
FDR=5%
internal alternative exons with m6A
no change (1665)
CTRL included more (0)
METTL3 KD included more (6)
% Inclusion, CTRL
% Inclusion, Mettl3 KD
FDR=5%
C
Guela et al 2015
internal consitutive exons with m6A
no change (9162)
WT included more (0)
KO included more (0)
% Inclusion, WT
% Inclusion, Mettl3 KO
FDR=5%
internal alternative exons with m6A
no change (1737)
WT included more (98)
KO included more (24)
% Inclusion, WT
% Inclusion, Mettl3 KO
FDR=5%
D
Liu et al 2015
internal consitutive exons with m6A
no change (7193)
CTRL included more (1)
METTL3 KD included more (0)
% Inclusion, CTRL
% Inclusion, Mettl3 KD
FDR=5%
internal alternative exons with m6A
no change (4376)
CTRL included more (189)
METTL3 KD included more (77)
% Inclusion, CTRL
% Inclusion, METTL3 KD
FDR=5%

FigS10

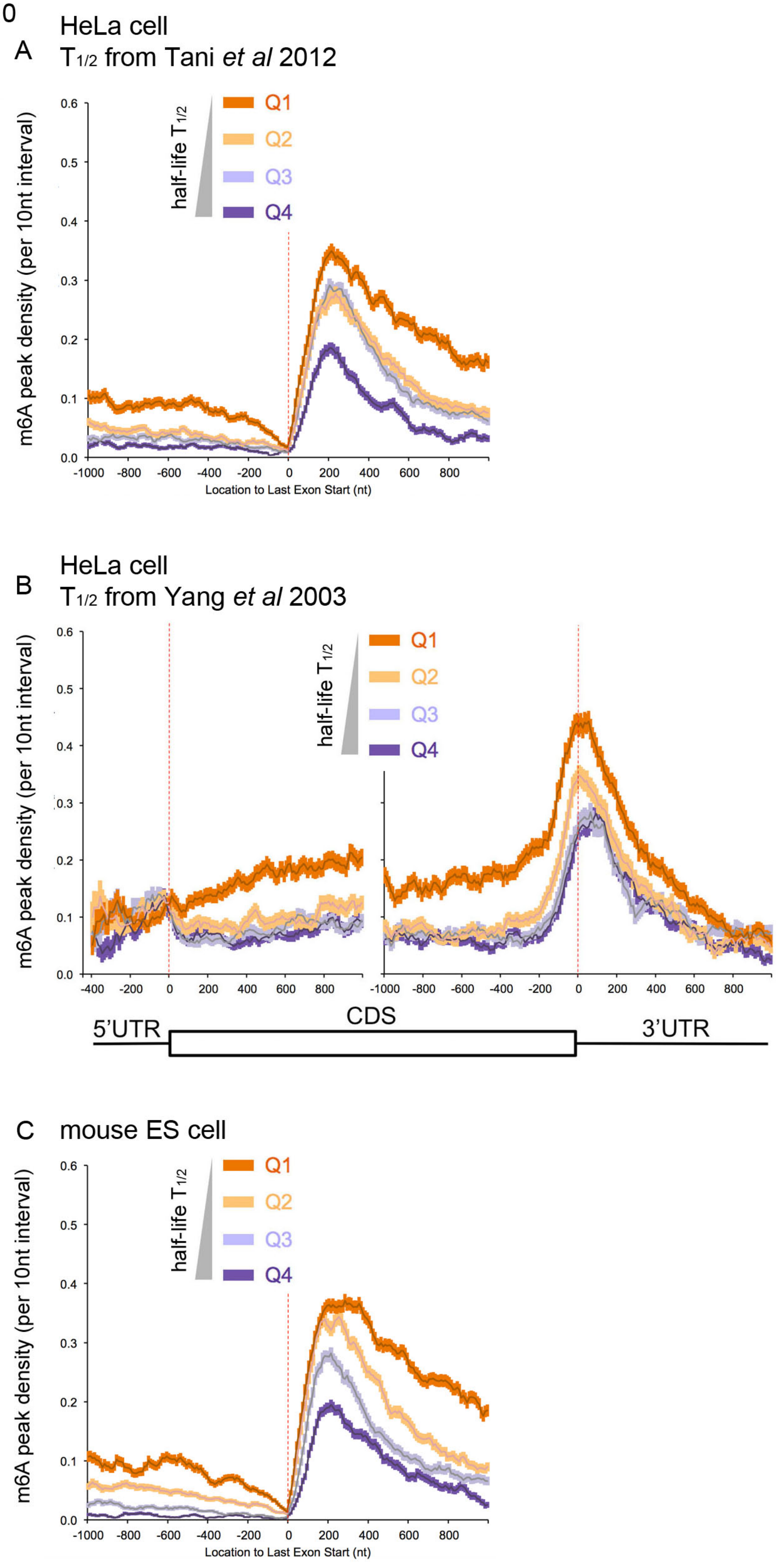
A
HeLa cell
T1/2 from Tani et al 2012
m6A peak density (per 10nt interval)
half-life T1/2
Q1
Q2
Q3
Q4
0.0
0.1
0.2
0.3
0.4
0.5
0.6
-1000
-800
-600
-400
-200
0
200
400
600
800
Location to Last Exon Start (nt)
B
HeLa cell
T1/2 from Yang et al 2003
m6A peak density (per 10nt interval)
half-life T1/2
Q1
Q2
Q3
Q4
-400
-200
0
200
400
600
800
-1000
-800
-600
-400
-200
0
200
400
600
800
5'UTR
CDS
3'UTR
C
mouse ES cell
m6A peak density (per 10nt interval)
half-life T1/2
Q1
Q2
Q3
Q4
Location to Last Exon Start (nt)

FigS11

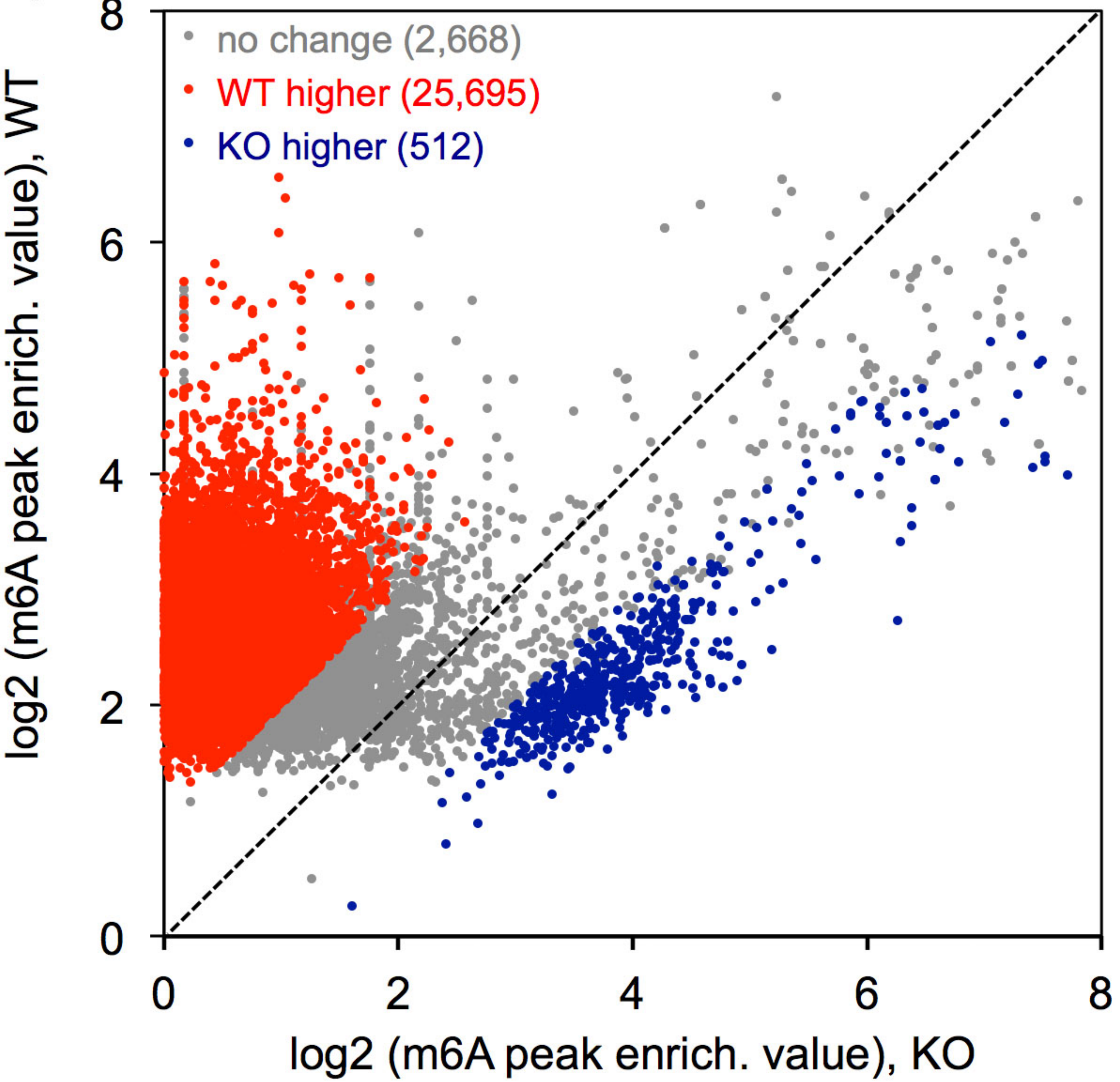
no change (2,668)
WT higher (25,695)
KO higher (512)
log2 (m6A peak enrich. value), WT
log2 (m6A peak enrich. value), KO
0
2
4
6
8

FigS12

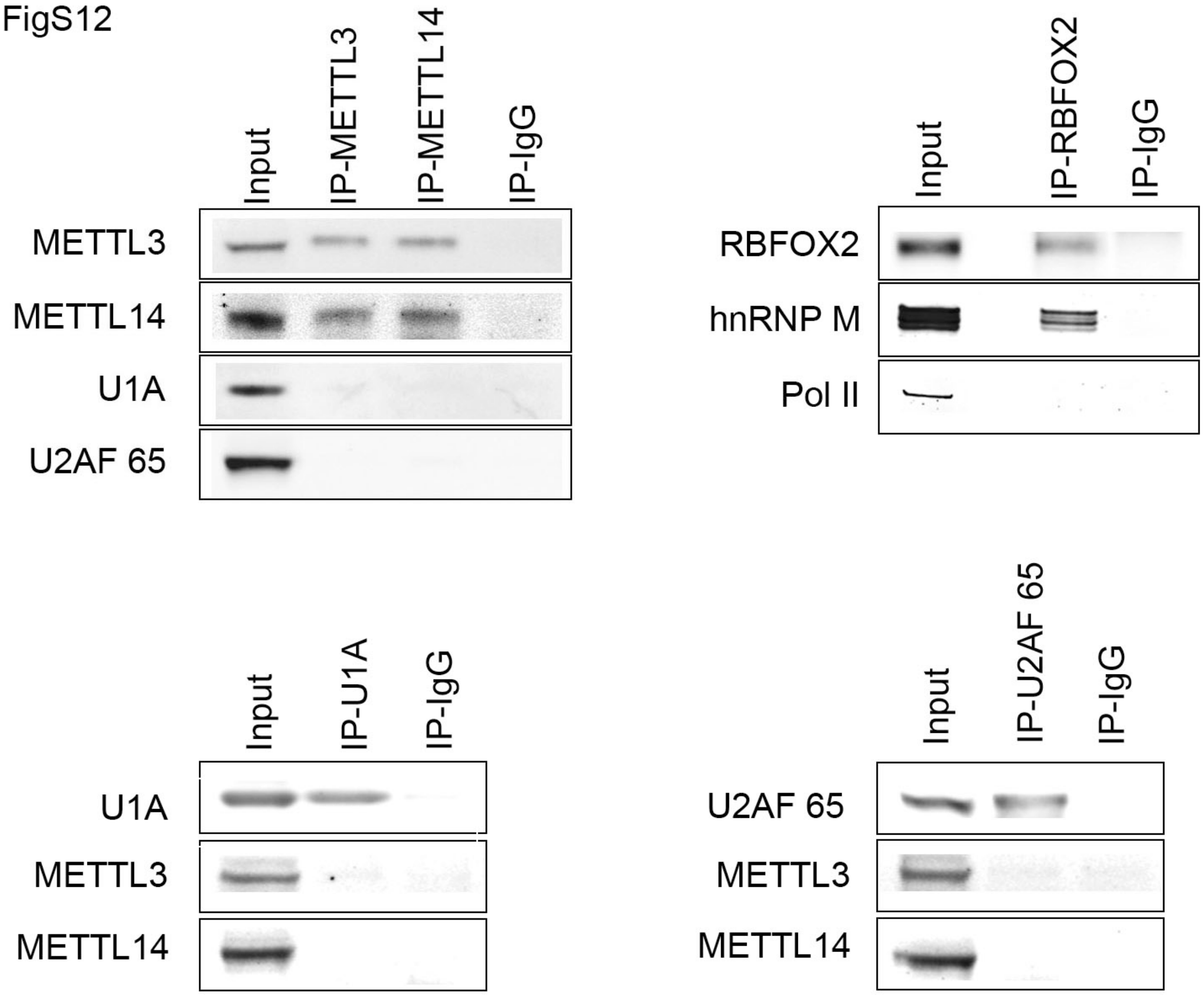

Input
IP-METTL3
IP-METTL14
IP-IgG
METTL3
METTL14
U1A
U2AF 65
Input
IP-RBFOX2
IP-IgG
RBFOX2
hnRNP M
Pol II
Input
IP-U1A
IP-IgG
U1A
METTL3
METTL14
Input
IP-U2AF 65
IP-IgG
U2AF 65
METTL3
METTL14

Supplemental Table 1. List of m6A sites that are nearby splice sites and yield m6A-containing exon-intron junction fragments in nascent RNA

	m6A site location	internal exon location	number of pre-mRNA reads containing m6A
1	chr12:+:53689670-53689671	chr12:+:53689622-53689725	23
2	chr22:+:45127624-45127625	chr22:+:45127609-45127701	20
3	chr11:+:12696956-12696957	chr11:+:12696955-12697108	18
4	chr17:-:39967420-39967421	chr17:-:39967383-39967536	18
5	chr14:+:20937680-20937681	chr14:+:20937485-20937694	14
6	chr17:+:17071212-17071213	chr17:+:17067422-17071229	13
7	chr2:+:232576639-232576640	chr2:+:232576596-232576693	13
8	chr2:+:232576639-232576640	chr2:+:232576599-232576693	13
9	chr17:-:39967420-39967421	chr17:-:39967383-39967536	12
10	chr2:+:85133207-85133208	chr2:+:85133126-85133241	12
11	chr19:+:11216252-11216253	chr19:+:11215895-11216276	11
12	chr11:-:10827564-10827565	chr11:-:10827453-10827594	11
13	chr12:-:115118716-115118717	chr12:-:115118683-115118951	11
14	chr2:+:232576639-232576640	chr2:+:232576596-232576693	10
15	chr2:+:232576639-232576640	chr2:+:232576599-232576693	10
16	chr5:-:40834341-40834342	chr5:-:40834282-40834367	10
17	chr5:+:177632031-177632032	chr5:+:177631815-177632047	10
18	chr19:+:11216243-11216244	chr19:+:11215895-11216276	9
19	chr1:-:150550749-150550750	chr1:-:150550719-150550967	9
20	chr11:-:2972531-2972532	chr11:-:2972488-2972545	9
21	chr11:+:65408308-65408309	chr11:+:65408301-65409071	9
22	chr18:-:692840-692841	chr18:-:692769-693175	9
23	chr11:-:61732894-61732895	chr11:-:61732840-61733049	8
24	chr11:-:61732894-61732895	chr11:-:61732840-61732987	8
25	chr17:-:74719899-74719900	chr17:-:74719853-74720140	8
26	chr16:+:731467-731468	chr16:+:731437-731603	8
27	chr12:+:104325378-104325379	chr12:+:104325298-104325401	8
28	chr7:-:72988438-72988439	chr7:-:72988267-72988452	7
29	chr7:-:72988423-72988424	chr7:-:72988267-72988452	7
30	chr7:-:51095421-51095422	chr7:-:51095408-51097288	7
31	chr14:-:81743267-81743268	chr14:-:81743244-81745083	7
32	chr8:-:62566217-62566218	chr8:-:62566126-62566219	7
33	chr1:-:153926080-153926081	chr1:-:153926013-153926082	7
34	chr6:-:74229645-74229646	chr6:-:74229605-74229779	7
35	chr11:-:122929365-122929366	chr11:-:122929339-122929538	7
36	chr2:+:85133201-85133202	chr2:+:85133126-85133241	7
37	chr9:-:130211579-130211580	chr9:-:130211559-130211641	7
38	chr9:+:131453502-131453503	chr9:+:131453448-131453506	7
39	chr7:-:99690932-99690933	chr7:-:99690914-99691024	6
40	chr8:-:144696982-144696983	chr8:-:144696956-144697085	6
41	chr8:+:42924789-42924790	chr8:+:42924697-42924802	6
42	chr8:+:146062888-146062889	chr8:+:146062775-146062892	6
43	chr8:+:146062888-146062889	chr8:+:146062778-146062892	6
44	chr19:-:3981367-3981368	chr19:-:3981336-3981450	6
45	chr17:-:26960603-26960604	chr17:-:26960566-26960769	6
46	chr17:-:62500423-62500424	chr17:-:62500339-62500436	6
47	chr16:+:2946752-2946753	chr16:+:2946352-2946772	6
48	chr12:+:123467063-123467064	chr12:+:123467015-123467085	6
49	chr12:+:123467055-123467056	chr12:+:123467015-123467085	6
50	chr12:+:104332117-104332118	chr12:+:104332117-104332237	6
51	chr15:+:44084518-44084519	chr15:+:44084173-44084581	6
52	chr15:+:44085250-44085251	chr15:+:44085172-44085281	6
53	chr4:-:1836593-1836594	chr4:-:1836571-1836709	6
54	chr2:-:27356182-27356183	chr2:-:27355984-27356205	6
55	chr2:-:242194985-242194986	chr2:-:242194788-242194995	6
56	chr2:+:54853351-54853352	chr2:+:54853068-54853371	6

57	chr9:-:130211590-130211591	chr9:-:130211559-130211641	6
58	chr9:+:131453473-131453474	chr9:+:131453448-131453506	6
59	chr9:+:135555176-135555177	chr9:+:135553363-135555190	6
60	chr9:+:131453473-131453474	chr9:+:131453448-131453506	6
61	chr13:-:107211857-107211858	chr13:-:107211779-107212005	6
62	chr5:+:177632895-177632896	chr5:+:177632842-177633011	6
63	chr14:-:68159674-68159675	chr14:-:68159650-68159769	5
64	chr14:+:102510803-102510804	chr14:+:102510610-102510828	5
65	chr8:+:146062888-146062889	chr8:+:146062775-146062892	5
66	chr8:+:146062888-146062889	chr8:+:146062778-146062892	5
67	chr19:-:3981367-3981368	chr19:-:3981336-3981450	5
68	chr19:+:13876787-13876788	chr19:+:13876767-13876943	5
69	chr19:+:11560216-11560217	chr19:+:11560080-11560243	5
70	chr19:+:13869791-13869792	chr19:+:13869681-13869824	5
71	chr19:+:11560207-11560208	chr19:+:11560080-11560243	5
72	chr1:-:150550768-150550769	chr1:-:150550719-150550967	5
73	chr1:+:63836705-63836706	chr1:+:63836441-63836730	5
74	chr1:+:156025166-156025167	chr1:+:156025053-156025216	5
75	chr1:+:150316939-150316940	chr1:+:150316909-150317023	5
76	chr6:+:33170190-33170191	chr6:+:33170039-33170204	5
77	chr11:-:118888710-118888711	chr11:-:118888667-118888763	5
78	chr11:+:75111836-75111837	chr11:+:75111737-75111868	5
79	chr17:-:17720904-17720905	chr17:-:17720861-17721230	5
80	chr17:+:73664698-73664699	chr17:+:73664595-73664705	5
81	chr3:-:101401631-101401632	chr3:-:101401614-101401751	5
82	chr3:-:101401625-101401626	chr3:-:101401614-101401751	5
83	chr3:+:52721269-52721270	chr3:+:52721261-52721399	5
84	chr12:-:109530707-109530708	chr12:-:109530311-109530742	5
85	chr15:+:44084538-44084539	chr15:+:44084173-44084581	5
86	chr2:-:232326270-232326271	chr2:-:232326250-232326728	5
87	chr2:-:232326264-232326265	chr2:-:232326250-232326728	5
88	chr9:-:123550501-123550502	chr9:-:123550046-123550556	5
89	chr9:-:123550522-123550523	chr9:-:123550046-123550556	5
90	chr10:-:101373500-101373501	chr10:-:101373452-101373681	5
91	chr7:-:151859336-151859337	chr7:-:151859201-151860911	4
92	chr22:-:36900168-36900169	chr22:-:36900144-36900414	4
93	chr8:+:145533517-145533518	chr8:+:145533457-145533582	4
94	chr8:+:145745644-145745645	chr8:+:145745621-145746206	4
95	chr19:-:3981413-3981414	chr19:-:3981336-3981450	4
96	chr19:+:13051355-13051356	chr19:+:13051354-13051468	4
97	chr19:+:13051382-13051383	chr19:+:13051354-13051468	4
98	chr19:+:13051382-13051383	chr19:+:13051354-13051468	4
99	chr1:-:94047917-94047918	chr1:-:94047857-94048510	4
100	chr6:-:52132745-52132746	chr6:-:52132662-52132766	4
101	chr6:+:33170383-33170384	chr6:+:33170336-33170477	4
102	chr11:-:10825848-10825849	chr11:-:10825833-10825965	4
103	chr11:-:61732877-61732878	chr11:-:61732840-61733049	4
104	chr11:-:61732877-61732878	chr11:-:61732840-61732987	4
105	chr11:-:10825854-10825855	chr11:-:10825833-10825965	4
106	chr11:+:71187117-71187118	chr11:+:71187078-71188484	4
107	chr11:+:73957120-73957121	chr11:+:73957120-73957250	4
108	chr11:+:67018227-67018228	chr11:+:67017556-67018269	4
109	chr17:-:26888616-26888617	chr17:-:26888439-26888647	4
110	chr17:-:3592021-3592022	chr17:-:3591919-3592053	4
111	chr17:-:40474307-40474308	chr17:-:40474299-40474512	4
112	chr17:-:40474307-40474308	chr17:-:40474302-40474512	4
113	chr17:-:26888645-26888646	chr17:-:26888439-26888647	4
114	chr17:-:73126895-73126896	chr17:-:73126847-73126962	4
115	chr16:-:88787146-88787147	chr16:-:88787023-88787156	4
116	chr12:-:109530687-109530688	chr12:-:109530311-109530742	4
117	chr12:+:104721293-104721294	chr12:+:104721292-104721449	4

118	chr12:+:104332228-104332229	chr12:+:104332117-104332237	4
119	chr12:+:112183967-112183968	chr12:+:112183947-112184088	4
120	chr12:+:98925604-98925605	chr12:+:98925457-98925616	4
121	chr12:+:52629137-52629138	chr12:+:52628938-52629150	4
122	chr15:+:91500839-91500840	chr15:+:91500833-91500955	4
123	chr15:+:44084530-44084531	chr15:+:44084173-44084581	4
124	chr2:+:234363488-234363489	chr2:+:234363408-234363519	4
125	chr2:+:220099603-220099604	chr2:+:220099547-220100034	4
126	chr2:+:220099612-220099613	chr2:+:220099547-220100034	4
127	chr2:+:54883074-54883075	chr2:+:54883050-54883135	4
128	chr9:-:19376512-19376513	chr9:-:19376491-19376649	4
129	chr10:-:97373613-97373614	chr10:-:97373498-97373620	4
130	chr5:+:36153096-36153097	chr5:+:36152872-36153144	4
131	chr7:+:115890518-115890519	chr7:+:115890214-115890550	3
132	chr7:+:142961238-142961239	chr7:+:142961178-142961260	3
133	chr20:-:57571831-57571832	chr20:-:57571693-57571856	3
134	chr20:-:57571839-57571840	chr20:-:57571693-57571856	3
135	chr22:-:39713520-39713521	chr22:-:39713465-39713634	3
136	chr22:+:42018035-42018036	chr22:+:42017993-42018090	3
137	chr14:+:102510791-102510792	chr14:+:102510610-102510828	3
138	chr14:+:102510761-102510762	chr14:+:102510610-102510828	3
139	chr8:-:99057121-99057122	chr8:-:99057054-99057316	3
140	chr8:-:62596744-62596745	chr8:-:62596597-62596747	3
141	chr8:+:145745675-145745676	chr8:+:145745621-145746206	3
142	chr8:+:143783017-143783018	chr8:+:143783017-143783131	3
143	chr8:+:145745649-145745650	chr8:+:145745621-145746206	3
144	chr19:-:3981405-3981406	chr19:-:3981336-3981450	3
145	chr19:-:3783821-3783822	chr19:-:3783811-3784031	3
146	chr19:+:42373781-42373782	chr19:+:42373768-42373823	3
147	chr19:+:42373792-42373793	chr19:+:42373768-42373823	3
148	chr1:-:3703455-3703456	chr1:-:3703412-3703874	3
149	chr1:-:156713997-156713998	chr1:-:156713954-156714140	3
150	chr1:+:156701844-156701845	chr1:+:156701782-156701907	3
151	chr1:+:156701844-156701845	chr1:+:156701782-156702265	3
152	chr1:+:16202707-16202708	chr1:+:16202696-16203173	3
153	chr1:+:46033690-46033691	chr1:+:46033653-46033849	3
154	chr1:+:16202726-16202727	chr1:+:16202696-16203173	3
155	chr1:+:203652503-203652504	chr1:+:203651869-203652526	3
156	chr6:-:52132714-52132715	chr6:-:52132662-52132766	3
157	chr6:-:52132714-52132715	chr6:-:52132662-52132766	3
158	chr6:-:112017623-112017624	chr6:-:112017479-112017659	3
159	chr6:-:18264099-18264100	chr6:-:18264073-18264227	3
160	chr6:+:32940633-32940634	chr6:+:32939371-32940704	3
161	chr11:-:62445303-62445304	chr11:-:62445252-62445304	3
162	chr11:-:118888710-118888711	chr11:-:118888634-118888763	3
163	chr11:-:104839267-104839268	chr11:-:104839245-104839455	3
164	chr11:-:47445654-47445655	chr11:-:47445596-47445734	3
165	chr11:-:62334448-62334449	chr11:-:62334277-62334482	3
166	chr11:-:47493811-47493812	chr11:-:47493742-47493909	3
167	chr11:+:18425322-18425323	chr11:+:18425240-18425358	3
168	chr11:+:63960729-63960730	chr11:+:63960549-63960759	3
169	chr11:+:12697043-12697044	chr11:+:12696955-12697108	3
170	chr11:+:65347407-65347408	chr11:+:65347406-65347460	3
171	chr17:-:48227327-48227328	chr17:-:48226507-48227383	3
172	chr17:-:26918749-26918750	chr17:-:26918715-26918890	3
173	chr17:-:18156722-18156723	chr17:-:18156614-18156772	3
174	chr17:+:40689468-40689469	chr17:+:40689415-40689563	3
175	chr17:+:73702099-73702100	chr17:+:73702087-73702172	3
176	chr21:+:33036113-33036114	chr21:+:33036102-33036199	3
177	chr16:+:85698630-85698631	chr16:+:85698620-85698734	3
178	chr3:-:49160167-49160168	chr3:-:49160136-49160485	3

179	chr3:-:156870915-156870916	chr3:-:156870824-156870945	3
180	chr3:+:184042879-184042880	chr3:+:184042665-184042902	3
181	chr3:+:184042798-184042799	chr3:+:184042665-184042902	3
182	chr3:+:128973514-128973515	chr3:+:128973510-128973586	3
183	chr12:-:109530707-109530708	chr12:-:109530311-109530742	3
184	chr12:-:6711578-6711579	chr12:-:6711541-6711663	3
185	chr12:-:109530678-109530679	chr12:-:109530311-109530742	3
186	chr12:+:6878769-6878770	chr12:+:6878768-6878840	3
187	chr12:+:112578976-112578977	chr12:+:112578622-112579028	3
188	chr12:+:98925592-98925593	chr12:+:98925457-98925616	3
189	chr12:+:104333312-104333313	chr12:+:104333286-104333403	3
190	chrX:+:48435466-48435467	chrX:+:48435413-48435497	3
191	chr4:-:187517707-187517708	chr4:-:187517693-187518325	3
192	chr4:+:1936969-1936970	chr4:+:1936870-1936989	3
193	chr2:-:232326300-232326301	chr2:-:232326250-232326728	3
194	chr2:-:242438798-242438799	chr2:-:242438671-242438829	3
195	chr2:-:242438783-242438784	chr2:-:242438671-242438829	3
196	chr2:-:215645309-215645310	chr2:-:215645283-215646233	3
197	chr2:+:234363485-234363486	chr2:+:234363408-234363519	3
198	chr2:+:233321309-233321310	chr2:+:233321289-233321405	3
199	chr2:+:54873330-54873331	chr2:+:54873330-54873612	3
200	chr9:-:139757419-139757420	chr9:-:139757357-139757451	3
201	chr9:-:139757389-139757390	chr9:-:139757357-139757451	3
202	chr13:-:45914874-45914875	chr13:-:45914846-45914920	3
203	chr13:-:45914874-45914875	chr13:-:45914846-45914955	3
204	chr13:-:79945109-79945110	chr13:-:79945079-79945297	3
205	chr5:-:176939631-176939632	chr5:-:176939496-176939646	3
206	chr5:-:37318114-37318115	chr5:-:37318089-37318187	3
207	chr5:-:176941934-176941935	chr5:-:176941916-176942070	3
208	chr5:-:134367161-134367162	chr5:-:134366965-134367198	3
209	chr5:-:176939635-176939636	chr5:-:176939496-176939646	3
210	chr5:+:14690185-14690186	chr5:+:14690147-14690417	3
211	chr5:+:14690265-14690266	chr5:+:14690147-14690417	3

Supplemental Table 2. List of several SR proteins binding site information (could be bound by SR proteins and act as ESEs, motifs are highly degenerate)

Name	Consensus Motif	Reference
SRSF1 (SF2/ASF)	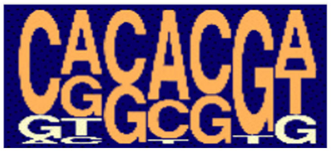	based on functional SELEX ESEfinder 3.0 http://rulai.cshl.edu/
SRSF2 (SC35)	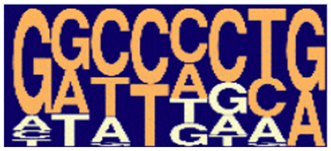	based on functional SELEX ESEfinder 3.0 http://rulai.cshl.edu/
SRSF5 (SRp40)	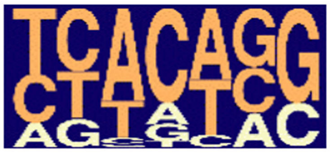	based on functional SELEX ESEfinder 3.0 http://rulai.cshl.edu/
SRSF6 (SRp55)	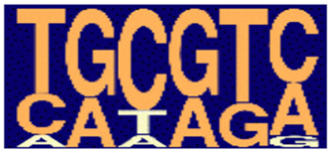	based on functional SELEX ESEfinder 3.0 http://rulai.cshl.edu/
SRSF3	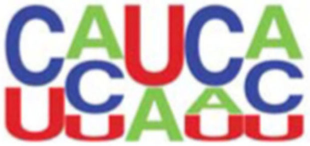	iCLIP Genome Biol. 2012; 13(3): R17
SRSF4	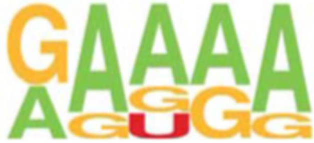	iCLIP Genome Biol. 2012; 13(3): R17
SRSF7	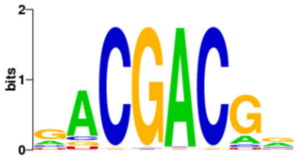	RNAcompete Nature 2013; 499, 172–177
SRSF9	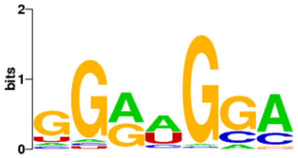	RNAcompete Nature 2013; 499, 172–177
SRSF10	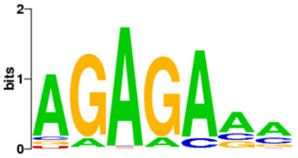	RNAcompete Nature 2013; 499, 172–177

*There are exon splicing enhancers (ESEs) and exon splicing silencers (ESSs) as well as intron splicing enhancers (ISEs) and intron splicing silencer sites (ISSs) that have no rigid space or sequence definitions (Liu et al., 2000; Cartegni et al., 2003; Black, 2003; Ke et al., 2008; Ke and Chasin, 2010). Such motifs are widely credited with providing ~50% of the information required for the choice of splice sites (Zhang et al., 2003; Chasin, 2007). While there is no obvious limitation to where these motifs that can affect splicing occur within RNAs, some are reported to lie near exon-intron boundaries (Fairbrother et al., 2002; Zhang and Chasin, 2004; Ke et al., 2011).

Black, D. L. (2003). Mechanisms of alternative pre-messenger RNA splicing. Annu Rev Biochem 72, 291-336.
Cartegni, L., Wang, J., Zhu, Z., Zhang, M. Q., and Krainer, A. R. (2003). ESEfinder: A web resource to identify exonic splicing enhancers. Nucleic Acids Res 31, 3568-3571.
Chasin, L. A. (2007). Searching for splicing motifs. Adv Exp Med Biol *623*, 85-106.
Fairbrother, W. G., Yeh, R. F., Sharp, P. A., and Burge, C. B. (2002). Predictive identification of exonic splicing enhancers in human genes. Science 297, 1007-1013.
Ke, S., and Chasin, L. A. (2010). Intronic motif pairs cooperate across exons to promote pre-mRNA splicing. Genome Biol 11, R84.
Ke, S., Shang, S., Kalachikov, S. M., Morozova, I., Yu, L., Russo, J. J., Ju, J., and Chasin, L. A. (2011). Quantitative evaluation of all hexamers as exonic splicing elements. Genome Res 21, 1360-1374.
Ke, S., Zhang, X. H., and Chasin, L. A. (2008). Positive selection acting on splicing motifs reflects compensatory evolution. Genome Res 18, 533-543.
Liu, H. X., Chew, S. L., Cartegni, L., Zhang, M. Q., and Krainer, A. R. (2000). Exonic splicing enhancer motif recognized by human SC35 under splicing conditions. Mol Cell Biol 20, 1063-1071.
Zhang, X. H., and Chasin, L. A. (2004). Computational definition of sequence motifs governing constitutive exon splicing. Genes Dev 18, 1241-1250.
Zhang, X. H., Heller, K. A., Hefter, I., Leslie, C. S., and Chasin, L. A. (2003). Sequence information for the splicing of human pre-mRNA identified by support vector machine classification. Genome Res 13, 2637-2650.

Supplemental Table 3. List of Gene Ontologies (GOs) that are significantly enriched in short T1/2 mRNAs with multiple m6As (>=2), and most GOs are associated with regulatory functions.

	GO.ID	Term	Annoatated	Significant	Expected	p.value	FDR
1	GO:0006351	transcription, DNA-templated	1856	572	347.83	6.39E-48	3.53E-44
2	GO:0006355	regulation of transcription, DNA-templat...	1798	560	336.96	2.52E-48	3.53E-44
3	GO:1903506	regulation of nucleic acid-templated tra...	1807	561	338.65	6.49E-48	3.53E-44
4	GO:2001141	regulation of RNA biosynthetic process	1809	561	339.02	9.81E-48	4.00E-44
5	GO:0097659	nucleic acid-templated transcription	1865	573	349.52	1.60E-47	5.24E-44
6	GO:0032774	RNA biosynthetic process	1880	574	352.33	1.33E-46	3.62E-43
7	GO:0051252	regulation of RNA metabolic process	1885	567	353.26	1.71E-43	4.00E-40
8	GO:0019219	regulation of nucleobase-containing comp...	2042	593	382.69	1.95E-40	3.99E-37
9	GO:2000112	regulation of cellular macromolecule bio...	2029	587	380.25	2.63E-39	4.76E-36
10	GO:0034654	nucleobase-containing compound biosynthe...	2048	588	383.81	3.18E-38	5.19E-35
11	GO:0001071	nucleic acid binding transcription facto...	495	213	92.72	3.93E-38	5.35E-35
12	GO:0003700	transcription factor activity, sequence-...	495	213	92.72	3.93E-38	5.35E-35
13	GO:0010556	regulation of macromolecule biosynthetic...	2064	590	386.81	1.00E-37	1.26E-34
14	GO:0031326	regulation of cellular biosynthetic proc...	2101	597	393.74	2.10E-37	2.45E-34
15	GO:0010468	regulation of gene expression	2142	605	401.43	3.80E-37	4.14E-34
16	GO:0009889	regulation of biosynthetic process	2125	601	398.24	5.08E-37	5.18E-34
17	GO:0019438	aromatic compound biosynthetic process	2078	589	389.43	2.30E-36	2.21E-33
18	GO:0018130	heterocycle biosynthetic process	2088	590	391.31	5.54E-36	5.03E-33
19	GO:0051171	regulation of nitrogen compound metaboli...	2196	611	411.55	2.51E-35	2.15E-32
20	GO:1901362	organic cyclic compound biosynthetic pro...	2128	593	398.8	3.66E-34	2.99E-31
21	GO:0060255	regulation of macromolecule metabolic pr...	2850	731	534.11	6.66E-31	5.18E-28
22	GO:0016070	RNA metabolic process	2420	641	453.53	4.16E-30	3.09E-27
23	GO:0080090	regulation of primary metabolic process	2842	726	532.61	6.40E-30	4.55E-27
24	GO:0034645	cellular macromolecule biosynthetic proc...	2590	673	485.39	2.74E-29	1.86E-26
25	GO:0031323	regulation of cellular metabolic process	2878	729	539.36	9.80E-29	6.40E-26
26	GO:0019222	regulation of metabolic process	3233	798	605.89	1.70E-28	1.07E-25
27	GO:0090304	nucleic acid metabolic process	2753	703	515.93	1.87E-28	1.13E-25
28	GO:0009059	macromolecule biosynthetic process	2631	676	493.07	8.79E-28	5.13E-25
29	GO:0046872	metal ion binding	1903	517	356.45	1.74E-25	9.79E-23
30	GO:0043169	cation binding	1923	517	360.2	2.78E-24	1.51E-21
31	GO:0044271	cellular nitrogen compound biosynthetic ...	2459	619	460.84	6.26E-22	3.30E-19
32	GO:0003677	DNA binding	1102	328	206.42	6.73E-22	3.44E-19
33	GO:0006139	nucleobase-containing compound metabolic...	2989	720	560.16	9.15E-21	4.53E-18
34	GO:0010467	gene expression	2826	686	529.62	2.47E-20	1.19E-17
35	GO:0044260	cellular macromolecule metabolic process	4328	970	811.1	6.69E-20	3.12E-17
36	GO:0006725	cellular aromatic compound metabolic pro...	3050	726	571.59	2.16E-19	9.78E-17
37	GO:0046483	heterocycle metabolic process	3054	723	572.34	1.51E-18	6.65E-16
38	GO:0044249	cellular biosynthetic process	2976	707	557.73	2.19E-18	9.40E-16
39	GO:0003676	nucleic acid binding	2371	583	444.12	1.48E-17	6.19E-15
40	GO:1901360	organic cyclic compound metabolic proces...	3126	732	585.84	1.86E-17	7.58E-15
41	GO:0050794	regulation of cellular process	4316	955	808.85	4.43E-17	1.77E-14
42	GO:1901576	organic substance biosynthetic process	3016	708	565.22	6.46E-17	2.51E-14
43	GO:0043170	macromolecule metabolic process	4526	991	848.21	1.39E-16	5.29E-14
44	GO:0009058	biosynthetic process	3049	712	571.41	2.08E-16	7.73E-14
45	GO:0050789	regulation of biological process	4540	989	850.83	1.21E-15	4.38E-13
46	GO:0043565	sequence-specific DNA binding	446	147	83.54	1.12E-13	3.99E-11
47	GO:0065007	biological regulation	4708	1007	882.32	3.27E-13	1.14E-10
48	GO:0000975	regulatory region DNA binding	386	129	72.3	1.33E-12	4.45E-10
49	GO:0044212	transcription regulatory region DNA bind...	386	129	72.3	1.33E-12	4.45E-10
50	GO:0001067	regulatory region nucleic acid binding	387	129	72.49	1.65E-12	5.38E-10
51	GO:0000976	transcription regulatory region sequence...	299	105	56.01	6.32E-12	1.98E-09
52	GO:0006357	regulation of transcription from RNA pol...	917	251	171.85	6.29E-12	1.98E-09
53	GO:0000981	RNA polymerase II transcription factor a...	258	94	48.33	7.89E-12	2.43E-09
54	GO:0001227	transcriptional repressor activity, RNA ...	80	42	14.98	9.70E-12	2.93E-09
55	GO:0006366	transcription from RNA polymerase II pro...	957	258	179.35	1.79E-11	5.32E-09
56	GO:1990837	sequence-specific double-stranded DNA bi...	317	107	59.38	6.26E-11	1.82E-08

57	GO:0034641	cellular nitrogen compound metabolic pro...	3394	748	636.06	8.74E-11	2.51E-08
58	GO:0003690	double-stranded DNA binding	363	117	67.99	2.12E-10	5.96E-08
59	GO:0000122	negative regulation of transcription fro...	403	126	75.53	3.87E-10	1.07E-07
60	GO:0006807	nitrogen compound metabolic process	3492	762	654.43	4.84E-10	1.32E-07
61	GO:0000977	RNA polymerase II regulatory region sequ...	259	89	48.51	9.72E-10	2.60E-07
62	GO:0045893	positive regulation of transcription, DN...	734	201	137.56	1.14E-09	2.95E-07
63	GO:1903508	positive regulation of nucleic acid-temp...	734	201	137.56	1.14E-09	2.95E-07
64	GO:1902680	positive regulation of RNA biosynthetic ...	736	201	137.93	1.46E-09	3.73E-07
65	GO:0043167	ion binding	2939	652	550.51	1.96E-09	4.94E-07
66	GO:0001012	RNA polymerase II regulatory region DNA ...	263	89	49.26	2.33E-09	5.78E-07
67	GO:0051254	positive regulation of RNA metabolic pro...	766	206	143.55	3.64E-09	8.88E-07
68	GO:1902679	negative regulation of RNA biosynthetic ...	642	177	120.32	7.48E-09	1.80E-06
69	GO:1903507	negative regulation of nucleic acid-temp...	635	174	119	1.66E-08	3.93E-06
70	GO:1901363	heterocyclic compound binding	3330	720	623.75	2.30E-08	5.37E-06
71	GO:0097159	organic cyclic compound binding	3352	724	627.87	2.46E-08	5.66E-06
72	GO:0045892	negative regulation of transcription, DN...	625	171	117.13	2.52E-08	5.72E-06
73	GO:0044237	cellular metabolic process	5010	1030	938.91	5.19E-08	1.16E-05
74	GO:0001078	transcriptional repressor activity, RNA ...	55	28	10.3	6.80E-08	1.50E-05
75	GO:0051253	negative regulation of RNA metabolic pro...	675	180	126.5	7.89E-08	1.72E-05
76	GO:0045935	positive regulation of nucleobase-contai...	854	218	160.05	1.33E-07	2.85E-05
77	GO:0000987	core promoter proximal region sequence-s...	167	59	31.28	2.29E-07	4.79E-05
78	GO:0001159	core promoter proximal region DNA bindin...	167	59	31.28	2.29E-07	4.79E-05
79	GO:0010558	negative regulation of macromolecule bio...	767	197	143.74	3.68E-07	7.61E-05
80	GO:0010628	positive regulation of gene expression	860	217	161.17	3.75E-07	7.66E-05
81	GO:0000982	transcription factor activity, RNA polym...	154	55	28.85	3.91E-07	7.89E-05
82	GO:0045934	negative regulation of nucleobase-contai...	745	192	139.62	4.09E-07	8.15E-05
83	GO:2000113	negative regulation of cellular macromol...	742	191	139.06	4.82E-07	9.49E-05
84	GO:0010629	negative regulation of gene expression	799	203	149.74	5.60E-07	0.0001088
85	GO:0031327	negative regulation of cellular biosynth...	784	199	146.93	7.92E-07	0.0001521
86	GO:0010605	negative regulation of macromolecule met...	1179	282	220.95	9.45E-07	0.0001795
87	GO:0010557	positive regulation of macromolecule bio...	830	208	155.55	1.16E-06	0.0002184
88	GO:0000978	RNA polymerase II core promoter proximal...	159	55	29.78	1.26E-06	0.0002345
89	GO:0044238	primary metabolic process	5002	1018	937.42	1.33E-06	0.0002444
90	GO:0009887	organ morphogenesis	353	102	66.16	1.42E-06	0.0002584
91	GO:0009890	negative regulation of biosynthetic proc...	794	199	148.8	2.04E-06	0.0003622
92	GO:0009891	positive regulation of biosynthetic proc...	869	215	162.86	2.03E-06	0.0003622
93	GO:0031328	positive regulation of cellular biosynth...	857	212	160.61	2.45E-06	0.0004303
94	GO:0023057	negative regulation of signaling	515	137	96.52	3.72E-06	0.0006404
95	GO:0051172	negative regulation of nitrogen compound...	824	204	154.42	3.69E-06	0.0006404
96	GO:0051173	positive regulation of nitrogen compound...	902	220	169.04	4.55E-06	0.0007736
97	GO:0071704	organic substance metabolic process	5135	1037	962.34	5.78E-06	0.0009727
98	GO:0010648	negative regulation of cell communicatio...	525	138	98.39	6.80E-06	0.0011329
99	GO:0009968	negative regulation of signal transducti...	481	128	90.14	7.75E-06	0.0012787
100	GO:0031324	negative regulation of cellular metaboli...	1178	276	220.77	7.83E-06	0.0012795
101	GO:0010604	positive regulation of macromolecule met...	1353	309	253.56	1.97E-05	0.0031853
102	GO:0009966	regulation of signal transduction	1090	255	204.27	2.12E-05	0.0033933
103	GO:0004930	G-protein coupled receptor activity	33	17	6.18	2.17E-05	0.0034451
104	GO:0031325	positive regulation of cellular metaboli...	1361	310	255.06	2.40E-05	0.0037662
105	GO:0009892	negative regulation of metabolic process	1290	295	241.76	2.88E-05	0.0044856
106	GO:0045944	positive regulation of transcription fro...	524	134	98.2	4.00E-05	0.0061706
107	GO:0031664	regulation of lipopolysaccharide-mediate...	6	6	1.12	4.30E-05	0.0065619
108	GO:0009888	tissue development	745	181	139.62	4.53E-05	0.0068508
109	GO:0000188	inactivation of MAPK activity	10	8	1.87	4.70E-05	0.0070019
110	GO:0099600	transmembrane receptor activity	117	40	21.92	4.71E-05	0.0070019
111	GO:0060037	pharyngeal system development	8	7	1.5	5.37E-05	0.0079088
112	GO:0038023	signaling receptor activity	146	47	27.35	6.07E-05	0.0088585
113	GO:0051216	cartilage development	62	25	11.62	6.16E-05	0.0089095
114	GO:0004888	transmembrane signaling receptor activit...	111	38	20.79	7.00E-05	0.0100258
115	GO:0006469	negative regulation of protein kinase ac...	120	40	22.49	9.05E-05	0.0128557
116	GO:0001933	negative regulation of protein phosphory...	207	61	38.79	0.000102	0.0143674
117	GO:0009893	positive regulation of metabolic process	1662	365	311.47	0.0001151	0.0160669

118	GO:0003002	regionalization	134	43	25.11	0.0001349	0.0186715
119	GO:0048705	skeletal system morphogenesis	87	31	16.3	0.0001396	0.0191598
120	GO:0007167	enzyme linked receptor protein signaling...	337	90	63.16	0.000155	0.0211012
121	GO:0045595	regulation of cell differentiation	642	156	120.32	0.0001567	0.0211607
122	GO:0007166	cell surface receptor signaling pathway	844	198	158.17	0.0001729	0.0231543
123	GO:0061035	regulation of cartilage development	22	12	4.12	0.0001797	0.0238729
124	GO:0045444	fat cell differentiation	100	34	18.74	0.000194	0.0253583
125	GO:0048585	negative regulation of response to stimu...	588	144	110.2	0.000194	0.0253583
126	GO:0046332	SMAD binding	45	19	8.43	0.000226	0.0290717
127	GO:0061448	connective tissue development	89	31	16.68	0.0002242	0.0290717
128	GO:0071499	cellular response to laminar fluid shear...	5	5	0.94	0.0002299	0.0293473
129	GO:0004872	receptor activity	196	57	36.71	0.0002471	0.0308109
130	GO:0048562	embryonic organ morphogenesis	117	38	21.93	0.0002463	0.0308109
131	GO:0060089	molecular transducer activity	196	57	36.71	0.0002471	0.0308109
132	GO:0007165	signal transduction	1895	408	355.14	0.0002524	0.0312412
133	GO:0048608	reproductive structure development	222	63	41.6	0.0002568	0.0315466
134	GO:0006464	cellular protein modification process	1901	409	356.26	0.0002638	0.0317231
135	GO:0023051	regulation of signaling	1198	269	224.51	0.000266	0.0317231
136	GO:0036211	protein modification process	1901	409	356.26	0.0002638	0.0317231
137	GO:1902531	regulation of intracellular signal trans...	682	163	127.81	0.000266	0.0317231
138	GO:0048523	negative regulation of cellular process	1933	415	362.26	0.0002828	0.0334788
139	GO:0007389	pattern specification process	168	50	31.48	0.000323	0.0379561
140	GO:0031399	regulation of protein modification proce...	806	188	151.05	0.000353	0.0411858
141	GO:0043412	macromolecule modification	2000	427	374.82	0.0003706	0.0429426
142	GO:0006468	protein phosphorylation	823	191	154.24	0.0004173	0.0480058
143	GO:0010646	regulation of cell communication	1236	275	231.64	0.0004243	0.0484739
144	GO:0061458	reproductive system development	226	63	42.35	0.0004367	0.0495458
145	GO:0003006	developmental process involved in reprod...	306	81	57.35	0.0004418	0.0497729

Supplemental Table 4. List of Gene Ontologies (GOs) that are significantly enriched in long T1/2 mRNAs with no m6A, and most GOs are associated with house keeping functions.

	GO.ID	Term	Annoatated	Significant	Expected	p.value	FDR
1	GO:0043230	extracellular organelle	1271	295	138.51	2.59E-44	2.12E-40
2	GO:1903561	extracellular vesicle	1270	295	138.4	2.18E-44	2.12E-40
3	GO:0065010	extracellular membrane-bounded organelle	1269	294	138.29	5.85E-44	2.39E-40
4	GO:0070062	extracellular exosome	1268	294	138.18	4.92E-44	2.39E-40
5	GO:0031982	vesicle	1614	334	175.88	2.03E-39	5.53E-36
6	GO:0031988	membrane-bounded vesicle	1556	326	169.56	1.89E-39	5.53E-36
7	GO:0044421	extracellular region part	1469	312	160.08	1.20E-38	2.79E-35
8	GO:0005576	extracellular region	1545	318	168.36	1.46E-36	2.98E-33
9	GO:0044445	cytosolic part	128	69	13.95	1.08E-33	1.97E-30
10	GO:0022626	cytosolic ribosome	74	51	8.06	1.54E-32	2.52E-29
11	GO:0044444	cytoplasmic part	3621	539	394.59	7.36E-25	1.09E-21
12	GO:0044391	ribosomal subunit	128	59	13.95	2.88E-24	3.93E-21
13	GO:1901564	organonitrogen compound metabolic proces...	966	204	104.13	4.80E-24	6.04E-21
14	GO:0098798	mitochondrial protein complex	97	50	10.57	1.68E-23	1.96E-20
15	GO:0032991	macromolecular complex	2703	429	294.56	3.49E-23	3.80E-20
16	GO:0005743	mitochondrial inner membrane	238	82	25.94	5.86E-23	5.98E-20
17	GO:0005737	cytoplasm	5171	693	563.5	7.37E-23	7.08E-20
18	GO:0022625	cytosolic large ribosomal subunit	44	32	4.79	5.48E-22	4.81E-19
19	GO:0098800	inner mitochondrial membrane protein com...	71	41	7.74	5.60E-22	4.81E-19
20	GO:0003735	structural constituent of ribosome	148	60	15.87	1.78E-21	1.45E-18
21	GO:0005740	mitochondrial envelope	356	102	38.79	2.30E-21	1.79E-18
22	GO:0019866	organelle inner membrane	268	85	29.2	4.76E-21	3.54E-18
23	GO:0031966	mitochondrial membrane	329	96	35.85	9.29E-21	6.60E-18
24	GO:0005840	ribosome	174	65	18.96	1.60E-20	1.09E-17
25	GO:0044455	mitochondrial membrane part	107	49	11.66	3.47E-20	2.27E-17
26	GO:0070469	respiratory chain	51	33	5.56	4.00E-20	2.51E-17
27	GO:0005829	cytosol	1016	200	110.72	4.61E-19	2.79E-16
28	GO:0005198	structural molecule activity	265	79	28.42	3.17E-18	1.85E-15
29	GO:0005746	mitochondrial respiratory chain	45	29	4.9	9.24E-18	5.20E-15
30	GO:0098803	respiratory chain complex	43	28	4.69	2.28E-17	1.20E-14
31	GO:1901566	organonitrogen compound biosynthetic pro...	691	147	74.48	2.26E-17	1.20E-14
32	GO:0005739	mitochondrion	1029	196	112.13	5.26E-17	2.68E-14
33	GO:0044429	mitochondrial part	522	121	56.88	6.14E-17	3.04E-14
34	GO:0030529	intracellular ribonucleoprotein complex	546	124	59.5	1.33E-16	6.22E-14
35	GO:1990904	ribonucleoprotein complex	546	124	59.5	1.33E-16	6.22E-14
36	GO:0043209	myelin sheath	109	45	11.88	1.70E-16	7.73E-14
37	GO:0015934	large ribosomal subunit	79	37	8.61	6.11E-16	2.70E-13
38	GO:0005747	mitochondrial respiratory chain complex ...	33	23	3.6	1.77E-15	7.22E-13
39	GO:0030964	NADH dehydrogenase complex	33	23	3.6	1.77E-15	7.22E-13
40	GO:0045271	respiratory chain complex I	33	23	3.6	1.77E-15	7.22E-13
41	GO:0006518	peptide metabolic process	479	109	51.63	4.68E-15	1.86E-12
42	GO:1902600	hydrogen ion transmembrane transport	47	27	5.07	6.13E-15	2.38E-12
43	GO:0098796	membrane protein complex	409	98	44.57	8.39E-15	3.19E-12
44	GO:0043043	peptide biosynthetic process	435	100	46.89	3.62E-14	1.35E-11
45	GO:0031967	organelle envelope	589	124	64.19	5.90E-14	2.10E-11
46	GO:0043603	cellular amide metabolic process	542	116	58.42	5.89E-14	2.10E-11
47	GO:0031975	envelope	590	124	64.29	6.73E-14	2.34E-11
48	GO:0006412	translation	427	98	46.03	7.50E-14	2.55E-11
49	GO:0015078	hydrogen ion transmembrane transporter a...	44	25	4.72	8.02E-14	2.67E-11
50	GO:0016469	proton-transporting two-sector ATPase co...	29	20	3.16	1.76E-13	5.75E-11
51	GO:1990204	oxidoreductase complex	63	30	6.87	2.08E-13	6.66E-11
52	GO:0022627	cytosolic small ribosomal subunit	24	18	2.62	2.86E-13	8.97E-11
53	GO:0043234	protein complex	2206	333	240.4	4.34E-13	1.34E-10
54	GO:0043604	amide biosynthetic process	464	101	50.01	1.03E-12	3.11E-10
55	GO:0006818	hydrogen transport	59	28	6.36	1.13E-12	3.29E-10
56	GO:0015992	proton transport	59	28	6.36	1.13E-12	3.29E-10
57	GO:0009141	nucleoside triphosphate metabolic proces...	118	40	12.72	1.12E-11	3.22E-09
58	GO:0045259	proton-transporting ATP synthase complex	15	13	1.63	2.40E-11	6.77E-09
59	GO:0015985	energy coupled proton transport, down el...	13	12	1.4	2.70E-11	7.34E-09
60	GO:0015986	ATP synthesis coupled proton transport	13	12	1.4	2.70E-11	7.34E-09
61	GO:0043226	organelle	6377	769	694.92	3.01E-11	8.05E-09
62	GO:0006754	ATP biosynthetic process	23	16	2.48	3.45E-11	9.10E-09
63	GO:0055114	oxidation-reduction process	434	92	46.78	5.49E-11	1.42E-08
64	GO:0055086	nucleobase-containing small molecule met...	270	66	29.1	6.56E-11	1.67E-08
65	GO:0009142	nucleoside triphosphate biosynthetic pro...	40	21	4.31	6.83E-11	1.72E-08
66	GO:0009145	purine nucleoside triphosphate biosynthe...	30	18	3.23	8.05E-11	1.96E-08
67	GO:0009206	purine ribonucleoside triphosphate biosy...	30	18	3.23	8.05E-11	1.96E-08

68	GO:0009205	purine ribonucleoside triphosphate metab...	101	35	10.89	1.10E-10	2.65E-08
69	GO:0009144	purine nucleoside triphosphate metabolic...	106	36	11.43	1.15E-10	2.68E-08
70	GO:0009199	ribonucleoside triphosphate metabolic pr...	106	36	11.43	1.15E-10	2.68E-08
71	GO:0042278	purine nucleoside metabolic process	147	44	15.85	1.16E-10	2.68E-08
72	GO:0009201	ribonucleoside triphosphate biosynthetic...	34	19	3.66	1.33E-10	3.01E-08
73	GO:0003723	RNA binding	1177	192	126.24	1.47E-10	3.28E-08
74	GO:0006753	nucleoside phosphate metabolic process	239	60	25.76	1.60E-10	3.43E-08
75	GO:0009126	purine nucleoside monophosphate metaboli...	112	37	12.07	1.58E-10	3.43E-08
76	GO:0009167	purine ribonucleoside monophosphate meta...	112	37	12.07	1.58E-10	3.43E-08
77	GO:0033177	proton-transporting two-sector ATPase co...	14	12	1.53	1.94E-10	4.11E-08
78	GO:0046128	purine ribonucleoside metabolic process	145	43	15.63	2.64E-10	5.54E-08
79	GO:0046034	ATP metabolic process	91	32	9.81	4.55E-10	9.42E-08
80	GO:0009117	nucleotide metabolic process	235	58	25.33	6.64E-10	1.36E-07
81	GO:0072521	purine-containing compound metabolic pro...	194	51	20.91	6.96E-10	1.40E-07
82	GO:0009123	nucleoside monophosphate metabolic proce...	129	39	13.9	9.75E-10	1.94E-07
83	GO:0003954	NADH dehydrogenase activity	18	13	1.93	1.17E-09	2.31E-07
84	GO:0009161	ribonucleoside monophosphate metabolic p...	120	37	12.93	1.43E-09	2.78E-07
85	GO:0005753	mitochondrial proton-transporting ATP sy...	13	11	1.42	1.54E-09	2.97E-07
86	GO:0043227	membrane-bounded organelle	6039	730	658.09	1.60E-09	3.04E-07
87	GO:0044281	small molecule metabolic process	791	138	85.26	1.68E-09	3.15E-07
88	GO:0009127	purine nucleoside monophosphate biosynth...	42	20	4.53	1.85E-09	3.39E-07
89	GO:0009168	purine ribonucleoside monophosphate bios...	42	20	4.53	1.85E-09	3.39E-07
90	GO:0045263	proton-transporting ATP synthase complex...	9	9	0.98	2.09E-09	3.79E-07
91	GO:0006163	purine nucleotide metabolic process	171	46	18.43	2.18E-09	3.91E-07
92	GO:0015935	small ribosomal subunit	50	22	5.45	2.31E-09	4.05E-07
93	GO:0044424	intracellular part	6703	792	730.45	2.31E-09	4.05E-07
94	GO:0030055	cell-substrate junction	228	56	24.85	2.42E-09	4.20E-07
95	GO:0009119	ribonucleoside metabolic process	161	44	17.35	2.87E-09	4.93E-07
96	GO:0009116	nucleoside metabolic process	168	45	18.11	3.78E-09	6.44E-07
97	GO:0005924	cell-substrate adherens junction	225	55	24.52	4.06E-09	6.76E-07
98	GO:0005925	focal adhesion	225	55	24.52	4.06E-09	6.76E-07
99	GO:0009150	purine ribonucleotide metabolic process	164	44	17.68	5.35E-09	8.83E-07
100	GO:1901657	glycosyl compound metabolic process	176	46	18.97	5.93E-09	9.69E-07
101	GO:0005912	adherens junction	259	60	28.22	7.12E-09	1.15E-06
102	GO:0008137	NADH dehydrogenase (ubiquinone) activity	17	12	1.82	8.03E-09	1.27E-06
103	GO:0050136	NADH dehydrogenase (quinone) activity	17	12	1.82	8.03E-09	1.27E-06
104	GO:0044422	organelle part	4326	551	471.42	8.65E-09	1.36E-06
105	GO:0009259	ribonucleotide metabolic process	173	45	18.65	1.02E-08	1.59E-06
106	GO:0070161	anchoring junction	265	60	28.88	1.77E-08	2.73E-06
107	GO:0042451	purine nucleoside biosynthetic process	56	22	6.04	2.40E-08	3.62E-06
108	GO:0046129	purine ribonucleoside biosynthetic proce...	56	22	6.04	2.40E-08	3.62E-06
109	GO:0016491	oxidoreductase activity	312	66	33.46	3.10E-08	4.65E-06
110	GO:0019693	ribose phosphate metabolic process	179	45	19.29	3.15E-08	4.68E-06
111	GO:0044446	intracellular organelle part	4272	542	465.54	3.22E-08	4.73E-06
112	GO:0009124	nucleoside monophosphate biosynthetic pr...	57	22	6.14	3.51E-08	5.12E-06
113	GO:0009165	nucleotide biosynthetic process	113	33	12.18	4.91E-08	7.03E-06
114	GO:1901293	nucleoside phosphate biosynthetic proces...	113	33	12.18	4.91E-08	7.03E-06
115	GO:0000502	proteasome complex	62	23	6.76	5.07E-08	7.20E-06
116	GO:0009156	ribonucleoside monophosphate biosyntheti...	50	20	5.39	7.29E-08	1.03E-05
117	GO:0016020	membrane	3513	456	382.82	1.13E-07	1.57E-05
118	GO:0006164	purine nucleotide biosynthetic process	76	25	8.19	1.66E-07	2.29E-05
119	GO:0045333	cellular respiration	76	25	8.19	1.66E-07	2.29E-05
120	GO:0031090	organelle membrane	1447	215	157.68	1.69E-07	2.30E-05
121	GO:0044822	poly(A) RNA binding	923	147	99	1.73E-07	2.34E-05
122	GO:0000276	mitochondrial proton-transporting ATP sy...	7	7	0.76	1.79E-07	2.39E-05
123	GO:0019752	carboxylic acid metabolic process	381	74	41.07	2.22E-07	2.95E-05
124	GO:0015077	monovalent inorganic cation transmembran...	88	27	9.44	2.44E-07	3.22E-05
125	GO:0006091	generation of precursor metabolites and ...	149	38	16.06	2.52E-07	3.28E-05
126	GO:0016655	oxidoreductase activity, acting on NAD(P...	21	12	2.25	2.53E-07	3.28E-05
127	GO:0009152	purine ribonucleotide biosynthetic proce...	73	24	7.87	2.97E-07	3.82E-05
128	GO:0036442	hydrogen-exporting ATPase activity	15	10	1.61	3.48E-07	4.45E-05
129	GO:0009163	nucleoside biosynthetic process	69	23	7.44	3.96E-07	4.98E-05
130	GO:0042455	ribonucleoside biosynthetic process	69	23	7.44	3.96E-07	4.98E-05
131	GO:1901659	glycosyl compound biosynthetic process	70	23	7.55	5.31E-07	6.62E-05
132	GO:0009260	ribonucleotide biosynthetic process	81	25	8.73	6.57E-07	8.14E-05
133	GO:0015988	energy coupled proton transmembrane tran...	16	10	1.72	8.79E-07	0.0001072
134	GO:0015991	ATP hydrolysis coupled proton transport	16	10	1.72	8.79E-07	0.0001072
135	GO:0046390	ribose phosphate biosynthetic process	83	25	8.95	1.10E-06	0.0001315
136	GO:0072522	purine-containing compound biosynthetic ...	83	25	8.95	1.10E-06	0.0001315
137	GO:0022900	electron transport chain	35	15	3.77	1.11E-06	0.0001327
138	GO:0015980	energy derivation by oxidation of organi...	112	30	12.07	1.53E-06	0.0001815

139	GO:0043436	oxoacid metabolic process	407	75	43.87	1.57E-06	0.0001842
140	GO:0019637	organophosphate metabolic process	429	78	46.24	1.65E-06	0.0001929
141	GO:0019829	cation-transporting ATPase activity	28	13	3	1.83E-06	0.0002124
142	GO:0044769	ATPase activity, coupled to transmembran...	14	9	1.5	2.20E-06	0.0002527
143	GO:0006082	organic acid metabolic process	411	75	44.3	2.30E-06	0.0002626
144	GO:0042625	ATPase coupled ion transmembrane transpo...	29	13	3.11	3.00E-06	0.0003402
145	GO:0051287	NAD binding	30	13	3.22	4.78E-06	0.000538
146	GO:0007338	single fertilization	30	13	3.23	5.04E-06	0.0005613
147	GO:0016614	oxidoreductase activity, acting on CH-OH...	58	19	6.22	5.05E-06	0.0005613
148	GO:0016616	oxidoreductase activity, acting on the C...	49	17	5.26	6.60E-06	0.0007284
149	GO:0090662	ATP hydrolysis coupled transmembrane tra...	19	10	2.05	7.48E-06	0.0008204
150	GO:0022904	respiratory electron transport chain	31	13	3.34	7.83E-06	0.0008533
151	GO:0005622	intracellular	6951	800	757.48	8.77E-06	0.0009491
152	GO:0044712	single-organism catabolic process	412	73	44.41	9.41E-06	0.0010113
153	GO:0016651	oxidoreductase activity, acting on NAD(P...	41	15	4.4	1.10E-05	0.0011729
154	GO:0098662	inorganic cation transmembrane transport	179	39	19.29	1.17E-05	0.0012406
155	GO:0098660	inorganic ion transmembrane transport	199	42	21.45	1.25E-05	0.0013172
156	GO:0044765	single-organism transport	1486	207	160.18	1.54E-05	0.0016125
157	GO:0006457	protein folding	108	27	11.64	2.02E-05	0.0021063
158	GO:0043229	intracellular organelle	6073	712	661.8	2.10E-05	0.0021719
159	GO:0034622	cellular macromolecular complex assembly	468	79	50.45	2.50E-05	0.0025687
160	GO:0015672	monovalent inorganic cation transport	159	35	17.14	2.59E-05	0.0026191
161	GO:0022624	proteasome accessory complex	21	10	2.29	2.57E-05	0.0026191
162	GO:0034220	ion transmembrane transport	280	53	30.18	2.60E-05	0.0026191
163	GO:0009055	electron carrier activity	39	14	4.18	2.77E-05	0.0027768
164	GO:0045184	establishment of protein localization	997	146	107.47	3.23E-05	0.0032167
165	GO:0008540	proteasome regulatory particle, base sub...	11	7	1.2	3.97E-05	0.0039259
166	GO:0022892	substrate-specific transporter activity	343	61	36.79	4.03E-05	0.0039642
167	GO:0065003	macromolecular complex assembly	794	120	85.59	4.50E-05	0.0044066
168	GO:0005215	transporter activity	405	69	43.44	5.30E-05	0.005152
169	GO:0015031	protein transport	926	136	99.81	5.48E-05	0.005301
170	GO:0001731	formation of translation preinitiation c...	15	8	1.62	5.68E-05	0.0053957
171	GO:0022890	inorganic cation transmembrane transport...	146	32	15.66	5.67E-05	0.0053957
172	GO:0044710	single-organism metabolic process	2293	297	247.16	5.68E-05	0.0053957
173	GO:0098655	cation transmembrane transport	198	40	21.34	5.74E-05	0.0054245
174	GO:1901575	organic substance catabolic process	864	128	93.13	6.29E-05	0.0059036
175	GO:0022891	substrate-specific transmembrane transpo...	262	49	28.1	6.36E-05	0.0059381
176	GO:0015075	ion transmembrane transporter activity	235	45	25.21	7.04E-05	0.0065359
177	GO:0009566	fertilization	47	15	5.07	7.42E-05	0.0068483
178	GO:0008553	hydrogen-exporting ATPase activity, phos...	6	5	0.64	7.68E-05	0.0070113
179	GO:1902578	single-organism localization	1608	217	173.33	7.67E-05	0.0070113
180	GO:0046961	proton-transporting ATPase activity, rot...	12	7	1.29	7.78E-05	0.0070589
181	GO:0007339	binding of sperm to zona pellucida	12	7	1.29	8.03E-05	0.0072073
182	GO:0035036	sperm-egg recognition	12	7	1.29	8.03E-05	0.0072073
183	GO:0002199	zona pellucida receptor complex	6	5	0.65	8.30E-05	0.0073328
184	GO:0005832	chaperonin-containing T-complex	6	5	0.65	8.30E-05	0.0073328
185	GO:0016272	prefoldin complex	6	5	0.65	8.30E-05	0.0073328
186	GO:0055085	transmembrane transport	402	68	43.33	8.73E-05	0.0076644
187	GO:0006812	cation transport	307	55	33.09	8.92E-05	0.0077955
188	GO:0006810	transport	1838	243	198.12	0.0001001	0.0086991
189	GO:0009988	cell-cell recognition	16	8	1.72	0.0001029	0.0088957
190	GO:0042773	ATP synthesis coupled electron transport	20	9	2.16	0.0001053	0.0090507
191	GO:0046907	intracellular transport	923	134	99.49	0.0001087	0.0092984
192	GO:0009056	catabolic process	1014	145	109.3	0.0001149	0.0097721
193	GO:0051021	GDP-dissociation inhibitor binding	4	4	0.43	0.0001316	0.011078
194	GO:0051022	Rho GDP-dissociation inhibitor binding	4	4	0.43	0.0001316	0.011078
195	GO:0051234	establishment of localization	1930	252	208.04	0.0001674	0.0140215
196	GO:0033176	proton-transporting V-type ATPase comple...	13	7	1.42	0.0001693	0.0141069
197	GO:0051536	iron-sulfur cluster binding	46	14	4.93	0.000215	0.0177424
198	GO:0051540	metal cluster binding	46	14	4.93	0.000215	0.0177424
199	GO:0005623	cell	7227	819	787.55	0.0002309	0.0189543
200	GO:0031597	cytosolic proteasome complex	10	6	1.09	0.0002349	0.0191881
201	GO:0044085	cellular component biogenesis	1305	178	140.67	0.0002364	0.0192117
202	GO:0005615	extracellular space	279	50	30.4	0.0002402	0.0194275
203	GO:0042273	ribosomal large subunit biogenesis	36	12	3.88	0.0002448	0.0197007
204	GO:0007005	mitochondrion organization	356	60	38.37	0.0002511	0.0201038
205	GO:1902582	single-organism intracellular transport	818	119	88.17	0.0002547	0.020296
206	GO:0016471	vacuolar proton-transporting V-type ATPa...	7	5	0.76	0.0002645	0.0209715
207	GO:0006811	ion transport	448	72	48.29	0.0002771	0.02176
208	GO:0051082	unfolded protein binding	47	14	5.04	0.0002765	0.02176
209	GO:0022618	ribonucleoprotein complex assembly	138	29	14.88	0.0002922	0.0228402

210	GO:0003743	translation initiation factor activity	42	13	4.5	0.0002987	0.0230153
211	GO:0044464	cell part	7212	817	785.92	0.0002979	0.0230153
212	GO:0090407	organophosphate biosynthetic process	220	41	23.71	0.0002969	0.0230153
213	GO:0005839	proteasome core complex	14	7	1.53	0.0003068	0.0234178
214	GO:0006886	intracellular protein transport	558	86	60.15	0.000306	0.0234178
215	GO:0033036	macromolecule localization	1363	184	146.92	0.0003165	0.0240506
216	GO:0008324	cation transmembrane transporter activit...	173	34	18.56	0.0003194	0.0241551
217	GO:0006119	oxidative phosphorylation	32	11	3.45	0.0003267	0.0245951
218	GO:0048471	perinuclear region of cytoplasm	327	56	35.63	0.0003505	0.026263
219	GO:0055080	cation homeostasis	180	35	19.4	0.0003553	0.0265033
220	GO:0007006	mitochondrial membrane organization	59	16	6.36	0.0003628	0.0269416
221	GO:0051649	establishment of localization in cell	1140	157	122.88	0.0003797	0.0280697
222	GO:0016787	hydrolase activity	1171	160	125.6	0.000388	0.0285511
223	GO:0008104	protein localization	1225	167	132.04	0.0003967	0.0290594
224	GO:0006839	mitochondrial transport	134	28	14.44	0.0004073	0.0297033
225	GO:0009060	aerobic respiration	33	11	3.56	0.000443	0.0320183
226	GO:0098771	inorganic ion homeostasis	182	35	19.62	0.0004412	0.0320183
227	GO:0000027	ribosomal large subunit assembly	19	8	2.05	0.0004496	0.0323554
228	GO:0022857	transmembrane transporter activity	291	50	31.21	0.0004617	0.0330808
229	GO:0004298	threonine-type endopeptidase activity	15	7	1.61	0.0004726	0.033569
230	GO:0070003	threonine-type peptidase activity	15	7	1.61	0.0004726	0.033569
231	GO:0022607	cellular component assembly	1146	157	123.53	0.0004875	0.0344767
232	GO:0090150	establishment of protein localization to...	136	28	14.66	0.0005241	0.0369015
233	GO:0051289	protein homotetramerization	34	11	3.66	0.0005919	0.0415003
234	GO:0036402	proteasome-activating ATPase activity	5	4	0.54	0.0006016	0.0418185
235	GO:0046933	proton-transporting ATP synthase activit...	5	4	0.54	0.0006016	0.0418185
236	GO:0007008	outer mitochondrial membrane organizatio...	8	5	0.86	0.0006096	0.0421971
237	GO:0061615	glycolytic process through fructose-6-ph...	5	4	0.54	0.0006133	0.042271
238	GO:0005766	primary lysosome	8	5	0.87	0.0006418	0.0438709
239	GO:0042582	azurophil granule	8	5	0.87	0.0006418	0.0438709
240	GO:0006520	cellular amino acid metabolic process	165	32	17.79	0.0006564	0.04468
241	GO:0015399	primary active transmembrane transporter...	51	14	5.47	0.0006957	0.04696
242	GO:0015405	P-P-bond-hydrolysis-driven transmembrane...	51	14	5.47	0.0006957	0.04696

Supplemental Table 5. List of Gene Ontologies (GOs) that are significantly enriched in short T1/2 mRNAs with no m6A, and most GOs are associated with nucleosome related functions.

	GO.ID	Term	Annoatated	Significant	Expected	p.value	FDR
1	GO:0000786	nucleosome	38	16	1.27	1.75E-14	2.87E-10
2	GO:0044815	DNA packaging complex	43	16	1.43	1.80E-13	1.47E-09
3	GO:0000788	nuclear nucleosome	13	8	0.43	1.54E-09	8.41E-06
4	GO:0006334	nucleosome assembly	54	13	1.82	1.74E-08	7.12E-05
5	GO:0032993	protein-DNA complex	92	16	3.07	4.91E-08	0.0001603
6	GO:0005179	hormone activity	9	6	0.3	9.85E-08	0.0002681
7	GO:0031497	chromatin assembly	66	13	2.22	2.24E-07	0.0005223
8	GO:0006323	DNA packaging	93	15	3.13	4.10E-07	0.0008376
9	GO:0034728	nucleosome organization	71	13	2.39	5.47E-07	0.0009925
10	GO:0006333	chromatin assembly or disassembly	81	13	2.73	2.61E-06	0.0042676
11	GO:0019731	antibacterial humoral response	9	5	0.3	4.72E-06	0.0070048
12	GO:0006335	DNA replication-dependent nucleosome ass...	16	6	0.54	8.33E-06	0.010472
13	GO:0034723	DNA replication-dependent nucleosome org...	16	6	0.54	8.33E-06	0.010472
14	GO:0019730	antimicrobial humoral response	11	5	0.37	1.64E-05	0.0190864
15	GO:0051290	protein heterotetramerization	18	6	0.61	1.82E-05	0.019873
16	GO:0065004	protein-DNA complex assembly	99	13	3.33	2.50E-05	0.0255527
17	GO:0002227	innate immune response in mucosa	7	4	0.24	4.07E-05	0.0391057
18	GO:0006342	chromatin silencing	64	10	2.16	4.84E-05	0.0439317

Supplemental Materials and Methods

Fractionation into chromatin-associated (CA), nucleoplasm and cytoplasm RNA groups

Hela cells were grown to 90% confluency in DMEM (Life Technologies) supplemented with 10% Fetal Bovine Serum (Omega), 2mM L-Glutamine (Life Technologies), 1U/mL Penacillin/Streptomycin (Life Technologies), 1x Non-essential Amino Acids (Life Technologies) and 0.1mM Beta-Mercaptoethanol (Sigma). Fractionations were performed as described previously(Pandya-Jones and Black, 2009). Briefly, cell pellets from a 90% confluent 10cm plate were gently scraped and collected in PBS at room temperature (4000rpm, 1min to pellet). Pellets were lysed in 200uL ice-cold Cytoplasmic Lysis Buffer for 5min on ice. Cellular lysates were passed through 500uL of an ice-cold Sucrose Cushion at 10000rpm, 4°C for 10min. The ~700uL cytoplasmic fraction was removed and used for protein analysis or further processed to obtain RNA samples (Pandya-Jones and Black, 2009). Pelleted nuclei were gently rinsed with 200uL ice-cold PBS and then re-suspended in 100uL ice-cold Glycerol Buffer prior to being lysed with 100uL ice-cold Nuclear Lysis Buffer. After vortexing for 2x2 seconds, nuclear lysates were incubated on ice for 2 minutes and then pelleted at 4°C, 14000rpm for 2 minutes. The soluble nuclear fraction was removed and used for protein analysis or further processed to obtain RNA samples (Pandya-Jones and Black, 2009). The chromatin pellet was gently rinsed with 200uL ice-cold PBS and then digested in 50uL 1xDNase I Buffer with 2U Turbo DNase (Thermo Fisher) at 37°C for 10 minutes. Protein samples were removed before dissolving the digested chromatin in 1mL of TRIzol (Life Technologies)

for RNA isolation. All centrifugations were conducted in eppendorf 5424 centrifuges either at room temperature or 4°C. All solutions were supplemented with 1x Complete Protease Inhibitors (Roche life sciences). In total, we prepared three biological replicates of cell fractionation into chromatin, nucleoplasm and cytoplasm. Both RNA-seq and m^6A mapping analysis are highly reproducible among biological replicates, and we presented the figure that integrates the result of three biological replicates for Figure 1.

List of antibodies that were used in this study: anti-m^6A, SYSY (Synaptic Systems) # 202003; anti-METTL3, Proteintech Group #15073-1-AP; anti-METTL14, Sigma-Aldrich #HPA038002; normal rabbit IgG, Santa Cruz Biotechnology#sc-2027; anti-U1A, Abcam#ab55751; anti-U2AF65, Sigma-Aldrich#U4758; anti-RPFOX2, Bethyl Laboratories#A300-864A; anti-hnRNP M, Novus Biologicals#NB200-314; anti-Pol II, Santa Cruz Biotechnology#sc-899.

We also included in the supplemental method a protocol for m^6A-CLIP (Ke et al., 2015) with a detailed day-to-day arrangement of experiments.

Quantification of m^6A using LC-MS/MS

The m^6A mass spectrometry quantification (Figure 6A) was performed similarly to the method that we described previously (Ke et al., 2015). In details, RNA was hydrolyzed to ribonucleosides by 20 U benzonase (Santa Cruz Biotech), 0.2 U nuclease P1, and 0.1 U alkaline phosphatase (Sigma) in 10 mM ammonium acetate pH 6.0 and 1 mM magnesium chloride at 40 °C for 40 min, added 3 volumes of acetonitrile and centrifuged (16,000 g,

30 min, 4 °C). The supernatants were dried and dissolved in 50 µl water for LC-MS/MS analysis of m^6A and unmodified ribonucleosides. Chromatographic separation was performed using an Agilent 1290 Infinity II UHPLC system with an ZORBAX RRHD Eclipse Plus C18 150 x 2.1 mm ID (1.8 µm) column protected with an ZORBAX RRHD Eclipse Plus C18 5 x 2.1 mm ID (1.8 µm) guard column (Agilent). The mobile phase consisted of water and methanol (both added 0.1 % formic acid) run at 0.25 ml/min, for m^6A starting with 5% methanol for 0.5 min, followed by a 4 min gradient of 5-90 % methanol, and 4 min re-equilibration with 5 % methanol. A portion of each sample was diluted for the analysis of unmodified ribonucleosides which was chromatographed isocratically with 20 % methanol. Mass spectrometric detection was performed using an Agilent 6495 Triple Quadrupole system operating in positive electrospray ionization mode, monitoring the mass transitions 282.1/150.1 (m6A), 268.1/136.1 (A), 244.1/112.1 (C), 284.1/152.1 (G), and 245.1/113.1 (U).

Determination of m^6A peak regions in exon vs. intron

To rigorously classify m^6A peaks into exonic vs. intronic regions, we used the complete set of transcript annotations according to GENCODE (version 19 of human hg19). If an m^6A peak was located to any known exonic region, it was classified as an exonic m^6A peak. If it was only located to an intronic region of transcripts, it was classified as an intronic m^6A peak. Using alternative annotations (e.g. Ensembl 85/version 25 for human GRCh38) generates essentially the same result. To examine the distribution of m^6A peaks in partially spliced mRNAs (Figure 1B and 1C), we focused on pre-mRNAs with abundant intronic RNA reads (i.e. intronic RNA reads were two times or more than

exonic RNA reads). Using data for all pre-mRNAs including those in which internal exons were completely or largely spliced (no or few introns), we obtained the same result: m^6As in CA-RNAs are >93% in exons (see Figure 1B).

Determining splicing completion for internal exons

The splicing completion for a specific internal exon (i.e. “spliced/(spliced+unspliced)(%)” in Figure 1A and “splicing completion index” in Figure 2A) is defined as the number of the spliced RNA molecules (S) divided by the number of total RNA molecules (spliced (S) plus unspliced (US))(Tilgner et al., 2012). S is calculated as the sum of two types of exon-exon junction reads: the exon-exon junction reads that spliced in this internal exon (0.5 x (a+b), a and b are the numbers of exon-exon junctions at each end of this internal exon, Figure 1) and that spliced out it (i.e. exon-exon junctions that connected the adjacent upstream exon to the adjacent downstream exon). US is calculated by enumerating the unspliced exon-intron junction reads (0.5 x (c+d), c and d are the numbers of exon-intron junctions at each end of this internal exon, Figure 1).

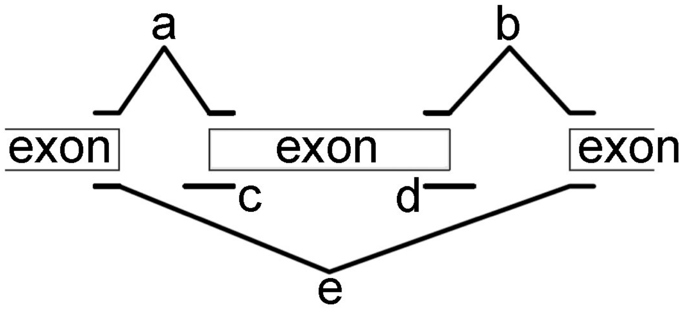

Figure 1. Definition of the splicing completion for a specific internal exon

The splicing completion for a specific intron (i.e. "spliced/(spliced+unspliced)(%)" in Supplemental Figure 1) is defined as the number of the spliced RNA molecules (S) divided by the number of total RNA molecules (spliced (S) plus unspliced (US)). In this case, S is the count of the exon-exon junction reads that have this intron spliced (a is the number of exon-exon junctions connecting the two ends of this intron, Figure 2) and US is the number of the unspliced exon-intron junction reads (0.5 x (b+c), b and c are the numbers of exon-intron junctions at each end of this intron, Figure 2). For all the exon-exon junction reads, we required at least 4nt overlap of the exon regions on both sides of the splice site. For exon-intron junction reads, we require at least 4nt overlap of the intron region on one end and of the exon region on the other end. To have reliable quantification, we focused on internal exons and introns with adequate read coverage by requiring at least 10 spliced and/or unspliced molecules. To remove possible contamination of intron retention events, we only considered internal exons and introns that were fully spliced in cytoplasm polyA+ RNA.

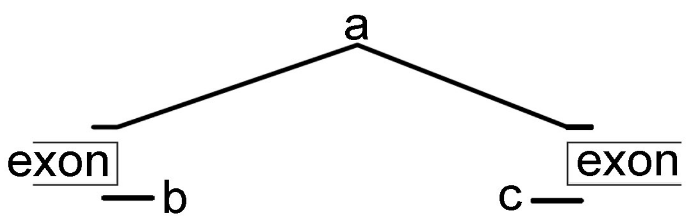

Figure 2. Definition of the splicing completion for a specific intron

Distribution of m^{6}A peak regions around start codons, stop codons and the start of last exons

To unambiguously assign m^6A peak regions to mRNAs according to gene annotations, we used a subset of GENCODE annotations (version 19 for human hg19 and version M10 for mouse mm10) by taking only one transcript isoform for each mRNA: we used the isoform with the longest mRNA length (alternatively we used the isoform with the most distal 3'end which generated essentially the same result). We then removed overlapping transcripts from the set to avoid any ambiguity in determining which transcript the m^6A peak region was from.

We considered m^6A peak regions that were within 1kb mRNA distance to stop codons and the fact that mRNAs had different lengths. We generated 100 intervals, each with a 10-nt size for 1 kb upstream of and downstream from the stop codons. We computed the "m^6A peak region density" (i.e. "m^6A peak density") for each interval as follows: we scanned through all mRNAs of interest that contained this interval and examined whether an m^6A peak region existed in this interval. We then enumerated those cases that contained m^6A peak regions and divided this value by the total number of mRNAs that contained this interval. For the plot of m^6A peak region density around the start of last exons and start codons, we performed the same analysis except anchoring at the start of the last exons and start codons.

Detection of pre-mRNA reads that have both intron and m^6A containing exon sequences

To identify pre-mRNA reads that have both intron and m^6A containing exon sequences, we focused on precisely mapped m^6A sites that are within 80nts of splice sites (the length

of our m^{6}A sequencing reads is ~80nt). We required the read length in intronic region to be at least 4nt for these pre-mRNA reads. Furthermore, we also required the number of pre-mRNA reads at each case for CA-RNA be more than 2 as the reliable evidence of existence. In addition, these pre-mRNA reads should not exist in nucleoplasm and cytoplasm RNAs. We identified over 200 internal exons to have m^{6}A-containing exon-intron junction fragments (the full list in Supplemental Table1 and 6 total examples in Figure 3B, 3C and Supplemental Figure 5).

Investigate m^{6}A locations to splice sites (SS)

To clearly show the distribution of m^{6}A peaks relative to splice sites, we focused on internal exons with exon length at least 200nt so that the 100nt exon regions from 5'SS and 3'SS would not overlap (Figure 4A and 4C). The internal exons that are at least 200nt long contain about 80% of all internal exon m^{6}As. "relative m^{6}A peak density" for a fixed position to a splice site (Figure 4A and 4C) was calculated as the m^{6}A peak density at that position scaled in proportion to the average m^{6}A peak density in exonic regions at least 100nt away from splice sites. The panel Figure 4B shows that about 7% exonic m^{6}As are within 50nts of splice sites for internal exons in Figure 4A (i.e. at least 200nt long). If we consider all m^{6}A-containing internal exons including those exons less than 100nt long in which all m^{6}A there are automatically within 50 nts distance to splice site, 20% exonic m^{6}As are within 50nt of splice sites.

Determination of m^{6}A peaks that are higher in CA-RNA

To determine m^{6}A peaks that are higher in CA-RNA, for each m^{6}A peak region, we

enumerated reads of m^6A-IP and the input for CA-RNA and Nucleoplasm RNA to evaluate the statistical significance (Fisher's exact test). Benjamini-Hochberg was implemented to adjust the P-value to the FDR for multiple testing. The requirement that an m^6A peak region is higher in CA-RNA included that (1) the reads of mRNAs in m^6A peak regions was adequate for m^6A peak region detection in both CA-RNA and nucleoplasmic mRNA (RPKM $\geq$ 1), and (2) the m^6A peak regions that are higher in CA-RNA were determined by requiring FDR $\leq$ 0.05 and an at least 2-fold higher of peak region enrichment in CA-RNA compared with nucleoplasmic mRNA. At a lower cutoff (e.g. 1.5 fold or higher), the same conclusion held, that most m^6A peaks are modified with the same level between CA-RNA and nucleoplasmic mRNA. Comparison of individual m^6A peak signal strength in nucleoplasmic RNA to cytoplasmic RNA for the same m^6A peak was performed in the same way as the comparison between CA-RNA and nucleoplasmic RNA.

Quantification of splicing inclusion

The splicing inclusion quantification was performed using Quantas (https://zhanglab.c2b2.columbia.edu/index.php/Quantas_Documentation). Significant changes in splicing are defined as delta PSI >=0.1 and FDR<5%, a statistical cutoff commonly used in splicing research for reliable splicing detection. Using another widely used splicing quantification software MISO (Katz et al., 2010) produced a similar result. We also analyzed the raw RNAseq data of previous publications that reported certain splicing changes upon comprising Mettl3 expression levels (Supplemental Fig 9, (Dominissini et al., 2012; Zhao et al., 2014; Geula et al., 2015) (Liu et al., 2015)), and

found the same result that exons splice mostly the same when their exonic m^6As were lost.

Determination of mRNA half-life ($T_{1/2}$)

We determined the $T_{1/2}$ of individual polyA+ mRNAs in mouse embryonic stem cells (ESC) and Mettl3 knockout (KO) by sequencing after 5 time points (0hr, 1hr, 2hrs, 4hrs and 8hrs) of actinomycin (final concentration of 5ug/mL, Sigma #A9415) treatment in three biological replicates. The $T_{1/2}$ was determined as ln(2)/k, k is the decay rate constant. The individual mRNA abundance levels at different time points after actinomycin D treatment were fitted to a first-order exponential decay curve to calculate the decay rate constant (k). A single $T_{1/2}$ was calculated for each mRNA by using all three replicate values at each time point. For downstream detailed analysis, we only considered the $T_{1/2}$ for individual mRNA with a reliable statistics (i.e. FDR<0.05, fitting the exponential decay model by t distribution) (Wang et al., 2002; Neff et al., 2012). We used the published mRNA $T_{1/2}$ data for HeLa cells (Tani et al., 2012) to overlay HeLa cell m^6A data with mRNA $T_{1/2}$ (Figure 5A). Using the mRNA $T_{1/2}$ data in human hepatoma cancer cells from an independent group(Yang et al., 2003), we obtained similar association of more m^6As in mRNAs and shorter $T_{1/2}$ (Supplemental Figure 10).

The m6A mini-gene for mRNA half-life validation was constructed based on a common retroviral GFP vector (Addgene#1764, puromycin as the selection marker for successful DNA integration). The mRNA regions containing m^6A sites were cloned in frame into the minigene at Multiple Clone Sites (MCS) that was at the end of the coding region of

GFP. Synonymous point mutations were carefully made to disrupt m^{6}A RAC core motif while not change the underlying protein coding sequence.

The detailed sequence for Ppp1r8 construct is (three precise m^{6}A sites by m^{6}A-CLIP (Ke et al., 2015) are marked in red, specific amino acid codons were marked out in coding region)

“AAGCGGATGGAGGGCTCTGGCTCTCTGGGCCTGGAGGAGTCGGGGAGCAGG CGCATGCAGAAC (AAC was mutated to AAT, Asn/N synonymous mutation) TTTGCCTTCAGTGGAGGA (GGA was mutated to GGT, Gly/G synonymous mutation) CTATATGGGGGCCTGCCCCCCACACACAGTGAAACGGGCTCCCAGCCGCATG GCATTCATGGGACAGCGCTCATTGGTGGCTTGCCCATGCCATACCCGAACCTC GCCCCTGATGTGGAC (GAC was mutated to GAT, Asp/D synonymous mutation) TTGACTCCTGTTGTGCCATCAGCAGTGGCCATAAATCCCACACCAAACCCTGC AGTCTATAACCCTGAGGCTGTGAATGAACCCAAGAAGAAGAAATACGCGAA GGAGGCTTGGCCGGGCAAGAAGCCCACACCTTCCTTACTGATT**TGA**TATTTTG GTTATGGAGAGGGTGG”;

The detailed sequence for Sox2 construct is (fifteen precise m^{6}A sites by m^{6}A-CLIP (Ke et al., 2015) are marked in red, specific amino acid codons were marked out in coding region)

“ATGGGCTCTGTGGTCAAGTCCGAGGCCAGCTCCAGCCCCCCCGTGGTTACCT CTTCCTCCCACTCCAGGGCGCCCTGCCAGGCCGGGGAC (GAC was mutated to GAT, Asp/D synonymous mutation) CTCCGGGAC (GAC was mutated to GAT, Asp/D synonymous mutation)

ATGATCAGCATGTACCTCCCCGGCGCCGAGGTGCCGGAGCCCGCTGCGCCCA

GTAGA (AGA was mutated to AGG, Arg/R synonymous mutation)

CTGCACATGGCCCAGCACTACCAGAGCGGCCCGGTGCCCGGCACGGCCATTA

AC (AAC was mutated to AAT, Asn/N synonymous mutation)

GGCACACTGCCCCTGTCGCACATG**TGA**GGGCTGGA (A mutated to T)

CTGCGAA (A mutated to T)

CTGGAGAAGGGGAGAGATTTTCAAAGAGATACAAGGGAATTGGGAGGGGTG

CAAAAAGAGGAGAGTAGGAAAAATCTGATAATGCTCAAAAGGAAAAAAAT

CTCCGCAGCGAAACGACAGCTGCGGAAAAAAA (A mutated to T)

CCACCAATCCCATCCAAATTAA (A mutated to T) CGCAAAAA (A mutated to T)

CCGTGATGCCGA (A mutated to T) CTAGAAAA (A mutated to T)

CTTTTATGAGAGATCTTGGGA (A mutated to T) CTTCTTTTGGGGGA (A mutated to T)

CTATTTTTGTACAGAGAAAACCTGAGGGCGGCGGGGAGGGCGGGGGAATCG

GA (A mutated to T) CCATGTATAGATCTGGAGGAAAAAAACTACGCAAAA (A mutated to T) CTTTTTTTTAAAGTTCTAGTGGTACGTTAGGCGCTTC"

Determination of m^{6}A peak regions lost in knockout (KO) of Mettl3 in mouse embryonic stem cells (ESC)

Global quantification of m6A by mass spectrometry was practiced according to a protocol described previously (Ke et al., 2015). To determine m^{6}A peak regions that were lost due to Mettl3 KO in mouse ESC (embryonic stem cells), we practiced the analysis as reported previously(Ke et al., 2015). For each m^{6}A peak region, we enumerated reads of

m^6A-IP and the input for wild-type (WT) and KO mous ESCs to evaluate the statistical significance (Fisher's exact test). Benjamini-Hochberg was implemented to adjust the P-value to the FDR for multiple testing. The requirement that an m^6A peak region is considered lost due to Mettl3 KO demanded that (1) the mRNAs containing these m^6A peak regions should be adequately expressed in both WT and KO mouse ESC (RPKM $\geq$ 1), (2) the expression of mRNAs in m^6A peak regions was adequate for m^6A peak region detection in both KO and WT (RPKM $\geq$ 1), and (3) the m^6A peak regions being lost due to Mettl3 KO were determined by requiring FDR $\leq$ 0.05 and an at least 2-fold decrease of peak region enrichment in KO compared with WT.

Reference

Dominissini D, Moshitch-Moshkovitz S, Schwartz S, Salmon-Divon M, Ungar L, Osenberg S, Cesarkas K, Jacob-Hirsch J, Amariglio N, Kupiec M, Sorek R, Rechavi G. 2012. Topology of the human and mouse m6A RNA methylomes revealed by m6A-seq. *Nature* **485**: 201–206.

Geula S, Moshitch-Moshkovitz S, Dominissini D, Mansour AA, Kol N, Salmon-Divon M, Hershkovitz V, Peer E, Mor N, Manor YS, Ben-Haim MS, Eyal E, Yunger S, Pinto Y, Jaitin DA, Viukov S, Rais Y, Krupalnik V, Chomsky E, Zerbib M, Maza I, Rechavi Y, Massarwa R, Hanna S, Amit I, Levanon EY, Amariglio N, Stern-Ginossar N, Novershtern N, Rechavi G, Hanna JH. 2015. Stem cells. m6A mRNA methylation facilitates resolution of naive pluripotency toward differentiation. *Science* **347**: 1002–1006.

Katz Y, Wang ET, Airoldi EM, Burge CB. 2010. Analysis and design of RNA sequencing experiments for identifying isoform regulation. *Nat Methods* **7**: 1009–1015.

Ke S, Alemu EA, Mertens C, Gantman EC, Fak JJ, Mele A, Haripal B, Zucker-Scharff I, Moore MJ, Park CY, Vagbo CB, Kussnierczyk A, Klungland A, Darnell JEJ, Darnell RB. 2015. A majority of m6A residues are in the last exons, allowing the potential for 3' UTR regulation. *Genes Dev* **29**: 2037–2053.

Liu N, Dai Q, Zheng G, He C, Parisien M, Pan T. 2015. N(6)-methyladenosine-dependent RNA structural switches regulate RNA-protein interactions. *Nature* **518**: 560–564.

Neff AT, Lee JY, Wilusz J, Tian B, Wilusz CJ. 2012. Global analysis reveals multiple pathways for unique regulation of mRNA decay in induced pluripotent stem cells. *Genome Res* **22**: 1457–1467.

Pandya-Jones A, Black DL. 2009. Co-transcriptional splicing of constitutive and alternative exons. *RNA* **15**: 1896–1908.
Tani H, Mizutani R, Salam KA, Tano K, Ijiri K, Wakamatsu A, Isogai T, Suzuki Y, Akimitsu N. 2012. Genome-wide determination of RNA stability reveals hundreds of short-lived noncoding transcripts in mammals. *Genome Res* **22**: 947–956.
Tilgner H, Knowles DG, Johnson R, Davis CA, Chakrabortty S, Djebali S, Curado J, Snyder M, Gingeras TR, Guigo R. 2012. Deep sequencing of subcellular RNA fractions shows splicing to be predominantly co-transcriptional in the human genome but inefficient for lncRNAs. *Genome Res* **22**: 1616–1625.
Wang Y, Liu CL, Storey JD, Tibshirani RJ, Herschlag D, Brown PO. 2002. Precision and functional specificity in mRNA decay. *Proc Natl Acad Sci U S A* **99**: 5860–5865.
Yang E, van Nimwegen E, Zavolan M, Rajewsky N, Schroeder M, Magnasco M, Darnell JEJ. 2003. Decay rates of human mRNAs: correlation with functional characteristics and sequence attributes. *Genome Res* **13**: 1863–1872.
Zhao X, Yang Y, Sun BF, Shi Y, Yang X, Xiao W, Hao YJ, Ping XL, Chen YS, Wang WJ, Jin KX, Wang X, Huang CM, Fu Y, Ge XM, Song SH, Jeong HS, Yanagisawa H, Niu Y, Jia GF, Wu W, Tong WM, Okamoto A, He C, Rendtlew Danielsen JM, Wang XJ, Yang YG. 2014. FTO-dependent demethylation of N6-methyladenosine regulates mRNA splicing and is required for adipogenesis. *Cell Res* **24**: 1403–1419.

A protocol for m^6A-CLIP (Ke et al., 2015) with a detailed arrangement of experiments into different days

O. RNA preparation (Day 1):

O1a: Protocol: Total RNAs were isolated from cells and tissues according to manufacturer's instructions using TRIzol reagent (Cat.# 15596, Life Technologies), then was suspended in RNA-free water, digested with RQ1 RNase-Free DNase (Cat. # M6101, Promega) for 20 min at 37°C and then subject to phenol/chloroform extraction and ethanol precipitation. The resulted total RNA pallet was re-suspended in RNA-free water. PolyA+ RNAs were selected using Dynabeads® mRNA Purification Kit (Cat.# 61006, Invitrogen) according to the manufacturer's protocol.

O1b: Recipes and steps:

RQ1 DNase treatment master mix (200ul reaction volume):

100ul	total RNA
20ul	10X RQ1 DNase Buffer
25ul	RQ1 DNase
5ul	RNasin® Ribonuclease Inhibitor
50ul	H2O

Incubate at 37°C, shake at 1200 rpm for 20 minutes

Phenol/Chloroform extraction master mix (after DNase treatment):

200ul	RQ1 DNase treatment post-reaction solution
200ul	Phenol
65ul	Chloroform

Vortex briefly, and incubate at 37°C, shake at 1200 rpm for 20 minutes, vortex briefly again, spin down @15000rpm for 5 minutes

Transfer the supernatant to the **Ethanol precipitation mixture:**

~200ul	supernatant
4ul	glycogen (if necessary)
25ul	NaOAc
800ul	Ethanol (100%)

Incubate at -80C or -20C for 1 hour or more.

Then spin at 14000rpm for 20 minutes 4C to have the pallet, wash twice with 75% ethanol, dry for 5 minutes, and re-suspend in water. For the downstream polyA+ RNA selection, we stick to the protocol provided by the vendor (https://www.thermofisher.com/us/en/home/references/protocols/nucleic-acid-purification-and-analysis/mrna-protocols/dynabeads-mrna-purification-kit.html#prot2)

A. M6A-IP: (Day 2~5)

A1a: Protocol: The PolyA+ RNA was fragmented to 20~80nt nucleotide fragments by alkaline hydrolysis (1mM EDTA, 6mM Na_2CO_3, 44mM NaHCO3, pH~9.3) at 95°C for 18 minutes, then the RNA fragments precipitated following standard ethanol precipitation. The fragmented polyA+ RNAs (3ug) were subject to PAGE selection for 20nt~80nt size.

A1b: Recipes and steps:

The fragmentation buffer is home-made and stored as 2X concentration (frozen, open for use only once to make sure freshness)

RNA fragmentation master mix (40ul reaction volume, incubate in PCR tube)
20ul 2X RNA fragmentation buffer
(2mM EDTA, 12mM Na_2CO_3, 88mM NaHCO3, pH~9.3)
20ul PolyA+ RNA

Incubate at 95°C for 18 minutes

Standard ethanol precipitation master mix
40ul RNA fragmentation solutions
8ul glycogen
10ul NaOAc
42ul H2O
400ul EtOH

Incubate at -80C or -20C for 1 hour or more.

A2a Overview: Dynabeads Protein A beads (250ul, Cat. # 10008D, Life Technologies) was pretreated with BSA (Cat. # B9000S, NEB) for 45 minutes at room temperature to

reduce background. Anti-m^6A rabbit polyclonal antibody (25ug, Cat. # 202003, Synaptic Systems) was then conjugated to Dynabeads Protein at room temperature for 1 hour.

A2b: Recipes and steps:

250μl Protein-A Dynabeads per sample

Wash 3 times with 1mL 1x Ab binding buffer (1X PBS, pH 7.4; 0.02% Tween-20)

Dynabeads Protein A beads BSA incubation mix: (total volume: ~256ul)
Protein-A Dynabeads
250ul 1x Ab binding buffer
6.25ul BSA (Cat. # B9000S, NEB)

After rotating at room temperature for 45 minutes, the beads is washed three times with 1mL 1x Ab Binding Buffer

Anti-m^6A rabbit polyclonal antibody incubation mix: (total volume: ~280ul)
Protein-A Dynabeads
25ul Anti-m^6A rabbit polyclonal antibody (25 ug)
6.25ul BSA (Cat. # B9000S, NEB)
250ul 1xAb binding Buffer

Rotate at RT for at least 45 minutes - 1 hour, then wash beads with 1mL 1xPXL buffer (1x PBS, 0.1% SDS, 0.5% Sodium Deoxycholate and 0.5% NP-40) three times before IP.

A3a Overview: The polyA+ RNA fragments (3ug) were incubated with the antibody in 1x PXL buffer (1x PBS, 0.1% SDS, 0.5% Sodium Deoxycholate and 0.5% NP-40) supplemented with 2% RNasin® Plus RNase Inhibitor (Cat. # N2615, Promega) for 2 hours at 4°C.

A3b: Recipes and steps:

m^6A-IP mixture: (300ul total liquid volume)
10ul RNA fragments
6ul RNasin® Plus RNase Inhibitor (Cat. # N2615, Promega)
150ul 2x PXL
134ul H_2O

Rotate at 4C for 2 hours.

A4a Overview: After incubation, the bead and antibody was washed 5 times by 4 different wash buffers: first twice by ice cold Nelson Low Salt Buffer (15mM Tris pH7.5 and 5mM EDTA), once by ice cold Nelson High Salt Buffer (15mM Tris pH7.5, 5mM EDTA, 2.5mM EGTA, 1% Triton X-100, 1% Sodium Deoxycholate, 0.1% SDS and 1M NaCl), once by ice cold Nelson Stringent Wash Buffer (15mM Tris pH7.5, 5mM EDTA, 2.5mM EGTA, 1% Triton X-100, 1% Sodium Deoxycholate, 0.1% SDS, 120mM NaCl and 25mM KCl) and last wash by ice cold NT-2 Buffer (50mM Tris pH7.4, 150mM NaCl, 1mM $MgCl_2$ and 0.05% NP-40).

A4b: Recipes and steps:

Rotating at 4C for 2 hours

➔ Wash beads twice with 1mL ice-cold Nelson Low Salt buffer (15mM Tris pH7.5 and 5mM EDTA)

➔ Once with 1mL ice-cold Nelson High Salt Buffer (15mM Tris pH7.5, 5mM EDTA, 2.5mM EGTA, 1% Triton X-100, 1% Sodium Deoxycholate, 0.1% SDS and 1M NaCl),

➔ Once by 1mL ice-cold Nelson Stringent Wash Buffer (15mM Tris pH7.5, 5mM EDTA, 2.5mM EGTA, 1% Triton X-100, 1% Sodium Deoxycholate, 0.1% SDS, 120mM NaCl and 25mM KCl)

➔ Last wash by 1mL ice-cold NT-2 Buffer (50mM Tris pH7.4, 150mM NaCl, 1mM $MgCl_2$ and 0.05% NP-40).

A5a: Overview: After the stringent wash series, the bead and antibody was split into two halves: one half performed m^6A-CLIP leg, and the other half had its bounded RNA eluted with 0.5mg/mL N6-methyladenosine sodium salt (Cat. # M2780, Sigma-Aldrich) at 4°C for 1 hour (m^6A-IP leg). The eluted RNA was ethanol precipitated and re-suspended in RNase-free water for downstream cDNA library preparation.

A5b: Recipes and steps:

M6A elution solution mix: (total volume 200ul)

20ul	N6-methyladenosine sodium salt (5mg/mL)
40ul	5 X IP buffer (1.5X SSPE; 5mM EDTA; 0.25% Tween-20)
8ul	RNasin® Plus RNase Inhibitor (Cat. # N2615, Promega)
132ul	H2O

Rotate at 4C for 1 hour, collect the supernatant solution, and perform standard ethanol precipitation.

A6a: Overview: The eluted RNA from the m6A-IP step and the input RNA fragments were first treated with T4 PNK to remove its 3'phosphor group and then ligated to 68 pmol preadenylated DNA linker (L32 from IDT) with T4 RNA ligase 2 and truncated KQ (New England Biolabs, catalog no. M0373L) overnight at 16°C. This ligation mixture was subject to 8% PAGE purification to harvest the ligated product.

A6b: Recipes and steps:

T4 PNK reaction mixture (total volume: 40ul)

10ul	RNA fragments
4ul	10x PNK buffer
4ul	T4 PNK enzyme
2ul	RNasin® Plus RNase Inhibitor (Cat. # N2615, Promega)
20ul	H2O

Incubate at 37C for 20minutes, and then receive standard ethanol precipitation to remove the PNK enzymes and its buffer.

Linker ligation reaction mixture (total volume: 30ul)

10ul	RNA fragments
3ul	10x ligation buffer
1ul	RNasin® Plus RNase Inhibitor (Cat. # N2615, Promega)
2ul	T4 RNA Ligase 2, truncated KQ (Cat. # M0373L, NEB)
1.25ul	L32 preadenylated DNA linker (68pmol)
6ul	50% PEG
6.75ul	H2O

Incubate at 16C overnight, and then receive standard ethanol precipitation to remove the ligase and its buffer. The resulted pallet is subject to 8% PAGE purification to harvest the ligated product.

B. M6A-CLIP: (Day 6~8)

B1a: Protocol: The remaining half of m^6A antibody was incubated with 1x IP buffer (0.3x SSPE, 1mM EDTA and 0.05% Tween-20), subject to UV crosslink (UV Stratalinker 2400, Stratagene) and then washed again 5 times by 4 different wash buffers: twice by Nelson Low Salt Buffer, once by Nelson High Salt Buffer, once by Nelson Strigent Wash Buffer and once by NT-2 Buffer to further reduce background.

B1b: Recipes and steps:

M6A-CLIP UV incubation mixture: (total volume 200ul)

40ul	5xIP buffer (1.5X SSPE; 5mM EDTA; 0.25% Tween-20)
4ul	RNasin® Plus RNase Inhibitor (Cat. # N2615, Promega)
156ul	H2O

The sample is transferred to a 6-well cell culture dish on ice and UV crosslink twice with 200 mJ cm^2 intensity per crosslink.
After UV crosslink, the beads is washed 5 times by 4 different wash buffers:
➔ 1mL ice-cold Nelson Low Salt Buffer twice
➔ 1mL ice-cold Nelson High Salt Buffer once
➔ 1mL ice-cold Nelson Stringent Wash Buffer once
➔ 1mL ice-cold NT-2 Buffer once

B2a: Protocol: After this wash series, the m^6A antibody received on-bead PNK treatment (T4 PNK, Cat. # M0201L, NEB) to remove 3'phospho group of its UV-crosslinked RNAs at 37°C for 20 minutes.

B2b: Recipes and steps:

The PNK reaction mixture: (total volume 40ul)

4ul	10x PNK buffer
4ul	T4 PNK enzyme
2ul	RNasin® Plus RNase Inhibitor (Cat. # N2615, Promega)
30ul	H2O

The mixture is incubated at 37C for 20 mins. (Shake at 1200rpm for 15" every 1'30")

B2a: Protocol: After 3 washes with 1x PNK buffer (50mM Tris-HCl pH 7.4, 10mM $MgCl_2$ and 0.5% NP-40) to remove remaining PNK enzyme, the m^6A antibody was

subject on-bead linker ligation with T4 RNA Ligase 2, truncated KQ (Cat. # M0373L, NEB) and 68 pmol pre-adenylated DNA linker (L32, ordered from IDT) overnight at 16°C.

B2b: Recipes and steps:

The T4 RNA Ligase mixture: (total volume 80ul)
45ul H2O
8ul 10x Buffer
2ul RNasin® Plus RNase Inhibitor (Cat. # N2615, Promega)
5ul L32 pre-adenylated DNA linker (68 pmol in total)
4ul T4 RNA Ligase 2, truncated KQ (Cat. # M0373L, NEB)
16ul 50% PEG

The mixture is incubated at 16C overnight (Shake at 1200rpm for 15" every 1'30")

B3a: Protocol: The ligated product were subject to ^{32}P-gamma-ATP PNK hot labeling, and then the m^6A antibody and its crosslinked RNA targets were eluted off beads by incubating with 6ul 1M DTT (Cat. # D0632, Sigma-Aldrich), 24ul 1x PNK buffer and 30ul Invitrogen 4x LDS sample buffer (Cat. # NP0007, Invitrogen) at 70 °C for 10 minutes.

B3b: Recipes and steps:

The ^{32}P-gamma-ATP PNK hot labeling mixture: (total volume 40ul)
4ul 10x PNK buffer
2ul T4 PNK enzyme
2ul 32P-gamma-ATP
30ul H2O
2ul RNasin® Plus RNase Inhibitor (Cat. # N2615, Promega)

Incubate at 37C for 10mins (Shake at 1200rpm for 15" every 1'30")

Then wash the beads 3 times with ice-cold 1xPNK buffer (1mL each time) to remove residual ^{32}P-gamma-ATP.

Elution mixture (total volume: 60ul)
6ul 1M DTT (Cat. # D0632, Sigma-Aldrich)

24ul 1x PNK buffer
30ul Invitrogen 4x LDS sample buffer (Cat. # NP0007, Invitrogen)

Incubate at 70C for 10minutes

B4a: Protocol: The eluted product received SDS-PAGE and was then transfer to a nitrocellulose membrane (Cat. # 162-0112, BIO-RAD) to harvest RNAs UV-crosslinked to m^6A antibody. The membrane bound with RNAs was cut out and digested of the protein by proteinase K (Cat. # 03115828001, Roche). The freed RNA (already ligated to L32 linker previously) was purified by phenol/chloroform extraction and ethanol precipitation, and then re-suspended in RNA-free water ready for downstream BrdU-CLIP library preparation.

B4b: Recipes and steps:

The 4mg/mL proteinase K solution: (total volume 240ul)
40ul 20mg/mL proteinase K solution stock
200ul 1xPK buffer (100mM Tris-HCl pH 7.5; 50mM NaCl; 10mM EDTA)

PK-7M Urea solution: (total volume ~500ul)
0.21g Urea
500ul 1xPK buffer
(Urea is hard to dissolve, prepare early to make sure the timely usage)

Add 200ul out of 240ul **The 4mg/mL proteinase K solution** to the cut RNA-bound membrane and incubate at 37C for 20 minutes (shaking at 1000rpm constantly); then add 200ul **PK-7M urea solution**, incubate for another 20 minutes at 37C (shaking at 1000rpm).

(In hood) add 400ul Phenol and 130ul Chloroform; vortex to mix and incubate @37C for 20 minutes (shanking at 1000rpm)
Vortex briefly, spin down @15000rpm for 5 minutes,

Transfer the supernatant to the standard ethanol precipitation mixture:

Incubate at -80C or -20C for 1 hour or more. Then spin at 14000rpm for 20 minutes 4C to have the pallet, wash twice with 75% ethanol, dry for 5 minutes, and re-suspend in water for downstream BrdU-CLIP.

C. BrdU-CLIP library preparation: (Day 9~10, this part is shared with standard CLIP experiment, in collaboration with Aldo Mele)

C1. Bead Prep: Denhardt's Blocking

Ab Binding Buffer:
1X PBS, pH 7.4
0.02% Tween-20

50μl Protein-G Dynabeads per sample (25μl per cDNA purification step), include -RT and/or -Template

Wash 3 times with Ab binding buffer

Add 225μl Ab binding buffer, 25μl 50X Denhardt's Solution (Sigma, D2532 or Invitrogen, 750018); total volume is 5X original bead volume

Rotate at RT for at least 45 minutes - 1 hour

C2. RT Reaction:

Add 8μl water to RNA pellet (tap to resuspend, quick spin down). Denature at 65°C for 5 minutes (in microfuge tube), place tube on ice (to avoid loss of RNA, do not over-dry pellet and do not pipette until after denaturing step)

Transfer to PCR tube (on ice)

Mix I:

4μl 5X RT Buffer
1μl dATP } 8.2mM (Invitrogen, 10297-018)
1μl dCTP }
1μl dGTP }
1μl Br-dUTP (8.2mM; Sigma, B0631)
1μl RT Primer (25μM)
9μl total

Add 9μl of Mix I

3 minutes at 75°C, ramp down to 55°C and hold

Mix II:

1μl DTT (0.1M)
1μl RNAsin Plus (Promega, N261)

1μl Superscript III
3μl total

Add 3μl of Mix II (pre-warm to 55°C in PCR block before adding)

30 minutes at 55°C, 5 minutes at 85°C, 4°C hold

C3. Bead Prep: Ab Binding

1X IP Buffer:

0.3X SSPE
1mM EDTA
0.05% Tween-20

Wash 3 times with Ab binding buffer

Add 20μl Ab binding buffer, 5μl 50X Denhardt's Solution and 25μl (5μg) αBrdU antibody (Millipore, MAB3222; Abcam, ab8955)

Rotate at RT for at least 45 minutes

Wash 3 times with 1X IP Buffer

Following RT reaction, add 1μl (at 2U/μl) RNAse H (Invitrogen 18021-071 or NEB M0297L)

Incubate for 20 minutes at 37°C, hold at 4°C

Add 10μl water (to bring volume above 25μl needed for G-25 column)

Spin through G-25 column to remove free BrdUTP (discard G-25 column as solid radioactive waste)

C4. cDNA Purification: Immunoprecipitation I

2X IP Buffer:

0.6X SSPE
2mM EDTA
0.1% Tween-20

Nelson Low Salt Buffer:

15mM Tris pH 7.5
5mM EDTA

Nelson Stringent Buffer:

15mM Tris-HCl pH7.5
5mM EDTA
2.5mM EGTA
1% Triton X-100
1% NaDOC
0.1% SDS
120mM NaCl
25mM KCl

Measure volume, add water up to 40µl and add 10µl 50X Denhardt's Solution and 50µl 2X IP Buffer for a total volume of 100µl (Denhardt's and 2X IP Buffer can be added to the G-25 column collection tube prior to spinning samples through, volume can then be adjusted up to 100µl)

5 minutes at 70°C, equilibrate to RT

Add to prepared tube of beads (25µl original slurry volume, store remaining beads for second purification at 4°C O/N), rotate at RT for 45 minutes

(all washes 3-5 minutes rotating at RT, 1ml)

Wash 1 time with 1X IP Buffer (5X Denhardt's)
2 times with Nelson Low Salt Buffer (1X Denhardt's)
2 times with Nelson Stringent Buffer (1X Denhardt's)
2 times with 1X IP Buffer

C5. Heat Elution:

Elution Buffer:

50µl 2X IP Buffer
40µl Water
90µl

Add 90µl elution buffer to each tube of beads

Incubate at 98°C for 1 minute in thermomixer (1200 rpm) to elute cDNA from beads and place on magnet

Collect eluate and add 10µl 50X Denhardt's to each for a total of 100µl

Store overnight at 4°C

C6. cDNA Purification: Immunoprecipitation II

<u>CircLigase Wash Buffer</u>:

33mM Tris-Acetate
66mM KCl
(pH 7.8)

5 minutes at 70°C, equilibrate to RT

Add to prepared tube of beads, rotate at RT for 45 minutes

(all washes 3-5 minutes rotating at RT, 1ml)

Wash 1 time with 1X IP Buffer (5X Denhardt's)
2 times with Nelson Low Salt Buffer (1X Denhardt's)
2 times with Nelson Stringent Buffer (1X Denhardt's)
2 times with CircLigase wash buffer

C7. Circularization: CircLigase

<u>Phusion Wash Buffer</u>:

50mM Tris
(pH 8.0)

<u>Reaction Mix</u>:

2μl	CircLigase 10X Reaction Buffer
4μl	Betaine (5M)
1μl	$MnCl_2$ (50mM)
1μl	CircLigase ssDNA Ligase II (100U) (Epicentre, CL9021K)
12μl	Water
20μl total	

Incubate 1 hour at 60°C in thermomixer (interval: shake at 1300rpm every 30" for 15")

(all washes 3-5 minutes rotating at RT, 1ml)

Wash 2 times with Nelson Low Salt Buffer
2 times with Nelson Stringent Buffer
2 times with Phusion wash buffer

C8. PCR: Phusion Polymerase, SYBR Green

Mix I:

10μl 5X Phusion HF Buffer
1μl 10mM dNTPs
37μl Water
48μl total

Mix II:

0.5μl P5 (20μM)
0.5μl P3 (20μM)
0.5μl Phusion DNA Polymerase (NEB, M0530)
1.5μl total

Add 48μl Mix I to beads

Incubate at 98°C for 1 minute in thermomixer (1200 rpm) to elute cDNA from beads and place on magnet

Collect eluate and place in PCR tube with optically clear cap

Add 1.5μl Mix II, 0.5μl 50X SYBR Green I (dilute 10,000X stock to 50X in Phusion wash buffer) to mix and place in real-time PCR machine

Cycle:

98°C 30”

98°C 10”
58°C 15”
72°C 20”

Remove reaction tube when RFU signal reaches ~250-500 (usually results in 2.5-5nM)

Purify PCR product using Agencourt AMPure XP beads (Beckman Coulter) according to manufacturers instructions

Quantitate using Tapestation, pool samples according to tapestation results.

MiSeq:
Dilute to 2nM, sequence on MiSeq at 5pM final concentration using standard Read 1 primer already on cartridge.

HiSeq:

Dilute to 2-10nM Submit for HiSeq sequencing using standard Read 1 sequencing primer listed below.

DP5_PE: (Allowing standard PE primer)
AATGATACGGCGACCACCGAGATCTACACTCTTTCCC*TAC**ACGACGCTCTTCCGATCT***

The 1st Sequencing primer (READ1)
ACACTCTTTCCC*TAC**ACGACGCTCTTCCGATCT***

SP3-PE
CAAGCAGAAGACGGCATACGAGATCTCGGCATTCCTG**CCGCTGGAAGTGACTGAC**AC

The 2nd Sequencing primer (SSP2)
TCGGCATTCCTGCCGCTGGAAGTGACTGACAC

L32
/5rApp/GTGTCAGTCACTTCCAGCGG/3ddc/ (RS of **CCGCTGGaaGTGaCTGaC**aC)

>LiK_PEDDD-1 (50nt)
/5Phos/DDDCGATNNNNNNNAGATCGGAAGAGCGTCGT/iSp18/CACTCA/iSp18/**CCGCTGGaaGTGaCTGaC**-3
>LiK_PEDDD-2 (50nt)
/5Phos/DDDTAGCNNNNNNNAGATCGGAAGAGCGTCGT/iSp18/CACTCA/iSp18/**CCGCTGGaaGTGaCTGaC**-3
>LiK_PEDDD-3 (50nt)
/5Phos/DDDATCGNNNNNNNAGATCGGAAGAGCGTCGT/iSp18/CACTCA/iSp18/**CCGCTGGaaGTGaCTGaC**-3
>LiK_PEDDD-4 (50nt)
/5Phos/DDDGCTANNNNNNNAGATCGGAAGAGCGTCGT/iSp18/CACTCA/iSp18/**CCGCTGGaaGTGaCTGaC**-3
>LiK_PEDDD-5 (50nt)
/5Phos/DDDCTAGNNNNNNNAGATCGGAAGAGCGTCGT/iSp18/CACTCA/iSp18/**CCGCTGGaaGTGaCTGaC**-3
>LiK_PEDDD-6 (50nt)
/5Phos/DDDGATCNNNNNNNAGATCGGAAGAGCGTCGT/iSp18/CACTCA/iSp18/**CCGCTGGaaGTGaCTGaC**-3
>LiK_PEDDD-7 (50nt)
/5Phos/DDDAGCTNNNNNNNAGATCGGAAGAGCGTCGT/iSp18/CACTCA/iSp18/**CCGCTGGaaGTGaCTGaC**-3
>LiK_PEDDD-8 (50nt)
/5Phos/DDDTCGANNNNNNNAGATCGGAAGAGCGTCGT/iSp18/CACTCA/iSp18/**CCGCTGGaaGTGaCTGaC**-3
